# Genetic interactions and pleiotropy in metabolic diseases: Insights from a comprehensive GWAS analysis

**DOI:** 10.1111/jcmm.70045

**Published:** 2024-09-05

**Authors:** Jing Shen, Julong Pan, Gang Yu, Hui Cai, Hua Xu, Hanfei Yan, Yu Feng

**Affiliations:** ^1^ The Affiliated Jiangsu Shengze Hospital of Nanjing Medical University Suzhou China; ^2^ The University of New South Wales Sydney New South Wales Australia; ^3^ The University of Melbourne Melbourne Victoria Australia

**Keywords:** genome‐wide association studies, metabolic diseases, pleiotropy, *PTPN22*, single nucleotide variants

## Abstract

This study offers insights into the genetic and biological connections between nine common metabolic diseases using data from genome‐wide association studies. Our goal is to unravel the genetic interactions and biological pathways of these complex diseases, enhancing our understanding of their genetic architecture. We employed a range of advanced analytical techniques to explore the genetic correlations and shared genetic variants of these diseases. These methods include Linked Disequilibrium Score Regression, High‐Definition Likelihood (HDL), genetic analysis combining multiplicity and annotation (GPA), two‐sample Mendelian randomization analyses, analysis under the multiplicity‐complex null hypothesis (PLACO), and Functional mapping and annotation of genetic associations (FUMA). Additionally, Bayesian co‐localization analyses were used to examine associations of specific loci across traits. Our study discovered significant genomic correlations and shared loci, indicating complex genetic interactions among these metabolic diseases. We found several shared single nucleotide variants and risk loci, notably highlighting the role of the immune system and endocrine pathways in these diseases. Particularly, rs2476601 and its associated gene *PTPN22* appear to play a crucial role in the connection between type 2 diabetes mellitus, hypothyroidism/mucous oedema and hypoglycaemia. These findings enhance our understanding of the genetic underpinnings of these diseases and open new potential avenues for targeted therapeutic and preventive strategies. The results underscore the importance of considering pleiotropic effects in deciphering the genetic architecture of complex diseases, especially metabolic ones.

## 1 INTRODUCTION

Globally, metabolic diseases are a significant public health issue with increasing prevalence due to aging populations and lifestyle changes.^1^, ^2^ Despite significant progress in the study of metabolic diseases in recent years, the genetic and biological links between these diseases have not been fully elucidated. In particular, the specific roles of single nucleotide polymorphisms (SNPs) in the occurrence and development of these diseases remain to be thoroughly explored.^3^, ^4^, ^5^

In this context, this study focuses on nine common metabolic diseases, including type 2 diabetes (T2D), hypertension, lipid metabolism disorders, hyperthyroidism, hypothyroidism/myxoedema, osteoporosis, gout, diabetic hypoglycaemia and Cushing's syndrome. By comprehensively analysing genome‐wide association studies (GWAS) summary statistics, we aim to uncover potential genetic links and shared biological mechanisms among these diseases. This research not only helps to enhance our understanding of the complex genetic architecture of metabolic diseases but also may provide important insights for future precision medicine and disease prevention strategies.

Currently, although GWAS has revealed multiple genetic variations among metabolic diseases,^6^, ^7^ many questions remain unanswered regarding the overall genetic correlations of these variations, whether they originate from individual gene loci or the entire genome, and whether these relationships are causal.^8^ Furthermore, this shared genetic aetiology implies potential pleiotropy, which often acts as a genetic confounder in the associations between traits,^9^, ^10^, ^11^ To decipher the shared pleiotropic genetic variation loci among multiple traits, cross‐trait analysis has been proposed. Its core idea is to leverage the correlations among GWAS signals^10^, ^11^, ^12^, ^13^ to further investigate the common genetic aetiology among various diseases and explore their potential pleiotropy. Such research methods help to deepen our understanding of the genetic interactions between different diseases, providing a more comprehensive perspective for exploring the genetic mechanisms of complex diseases. Therefore, studying specific genomic variation loci, in the context of whole‐genome genetic correlations, and further dissecting the shared genetic aetiology among these diseases is of great significance.^14^

To achieve this goal, we screened GWAS data from the IEU database,^15^ the FinnGen database,^16^ and the UK Biobank database,^17^ which cover extensive genetic variation information in European ancestry populations. In selecting the analytical methods, we employed advanced statistical tools such as Linkage Disequilibrium Score Regression (LDSC),^18^ Heritability Estimation via Linkage Disequilibrium (HDL)^19^ and Genetic Analysis of Polygenic Traits (GPA)^20^ to identify genetic associations between different diseases. We used tools like Pleiotropy Analysis using Combined Omics (PLACO)^21^ and Functional Mapping and Annotation of GWAS (FUMA)^22^ to identify shared pleiotropic gene loci among these diseases. We revealed the characteristics of these pleiotropic gene loci through gene annotation, colocalization analysis and in‐depth exploration of biological mechanisms, including their functions, regulatory properties and potential pathogenicity. We validated these potential pleiotropic genes using Multi‐marker Analysis of GenoMic Annotation (MAGMA).^23^ In addition, we used Mendelian randomization (MR) approach to remove confounders and pleiotropy as a complement to and validation of genetic relationships between diseases. This series of meticulous research steps is expected to provide profound and comprehensive insights into the genetic basis of these diseases.

The core objective of this study is to uncover the genetic correlations between metabolic diseases and identify pleiotropic SNPs and loci associated with multiple metabolic diseases. We found that these pleiotropic variations play significant roles in key biological pathways, such as the immune and endocrine systems, and may provide crucial clues for uncovering the shared genetic aetiology of metabolic diseases. For instance, we observed a significant association of the gene variant rs2476601 (*PTPN22*) with hypothyroidism, T2D and hypoglycaemia. Current research on *PTPN22* mainly focuses on rheumatoid arthritis,^24^, ^25^ type 1 diabetes and autoimmune thyroid disease.^26^ However, the molecular genetic role of *PTPN22* in T2D, particularly its immunological relationship with Hashimoto's thyroiditis (HT), remains unexplored.

In summary, our study highlights the importance of genome‐wide genetic analysis in understanding metabolic diseases and provides a foundation for further exploration of their shared genetic basis. Despite some limitations, such as the racial homogeneity of the sample and the inability to capture rare variants, our findings still offer valuable insights for further research into the genetic and molecular mechanisms of metabolic diseases.

## 2 METHODS

### 2.1 Data sources

#### 2.1.1 GWAS data

In this study, all GWAS data were sourced from three primary databases: IEU, FinnGen and UK Biobank. These databases provided comprehensive information on nine common metabolic diseases: T2D (ebi‐a‐GCST006867), hypertension (ukb‐d‐I9_HYPTENS), disorders of lipoid metabolism (ukb‐e‐272_CSA), hyperthyroidism/thyrotoxicosis (ukb‐b‐20289), hypothyroidism/myxoedema (ukb‐b‐19732), osteoporosis (ukb‐b‐12141), gout (ukb‐b‐13251), diabetic hypoglycaemia (finn‐b‐DM_HYPOGLYC) and Cushing's syndrome (finn‐b‐E4_CUSHING).

Additionally, data on 91 circulating inflammatory proteins were obtained from GWAS measurements using the Olink Target Inflammation Panel. This data was collected across 11 cohorts, involving a total of 14,824 participants of European ancestry.^27^

**FIGURE 1:**
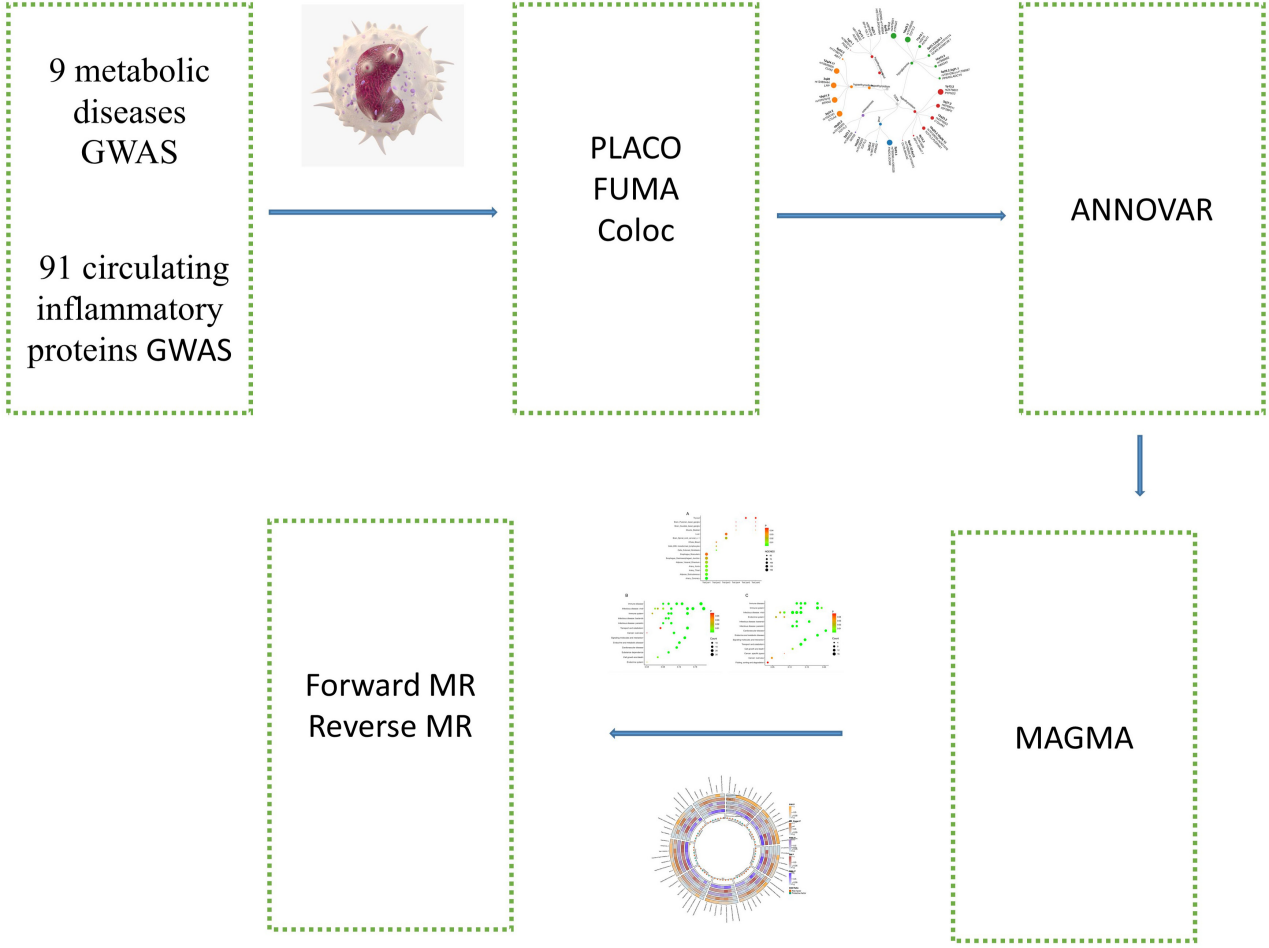
Flow chart.

Detailed descriptions of the samples are available in key publications, and it's important to note that all GWAS data used in our analysis pertained to populations of European ancestry (Table S1). The experimental design is shown in Figure 1.

### 2.2 Statistical analysis

All analyses in this study were performed after excluding single nucleotide variants (SNVs) in MHC regions (chromosome 6: 25–35 MB).

#### 2.2.1 Genome‐wide genetic correlations and genetic overlap

In this section of the analysis, we initially assessed genome‐wide genetic correlations across 36 pairs of traits between two sets of nine metabolic diseases. This was accomplished using LDSC.^18^ For the LDSC analyses, we employed LD scores based on the 1000 Genomes Project, specifically focusing on the European ancestry dataset.^28^ An important aspect of our LDSC analysis was the decision not to restrict the intercept. While sample overlap can influence the intercept, it does not affect the slope. Therefore, the genetic correlations remain unaffected even in cases of sample overlap. This approach not only allows for consideration of residual confounders but also helps identify potential overlaps in samples between two GWAS studies.^18^

Subsequently, we employed HDL to re‐validate the genome‐wide genetic correlations for these 36 trait pairs. HDL is a novel method for analysing GWAS data, designed to better understand the genetic basis of complex traits by precisely estimating genetic correlations and heritability. The HDL method combines advanced statistical techniques and computational tools to provide higher resolution results compared to traditional methods. Compared to LDSC, HDL offers greater accuracy in estimating genetic correlations.^29^ This step added robustness to our findings, ensuring that the genetic correlations identified were reliable and accurately represented the relationships between the metabolic diseases studied.

Finally, we delved into the overall genetic overlap between traits using the GPA. GPA is a comprehensive approach that combines multiple GWAS datasets and functional annotations to detect signals of association. This method is particularly effective in identifying weaker signals that might be overlooked in other analytical approaches. Additionally, GPA provides insights into the relationships between the genetic structures of different traits.^12^ By applying GPA, we aimed to enhance the depth and breadth of our understanding of the genetic overlaps, thereby uncovering the intricate genetic interplay among the various metabolic diseases studied.

Using three different analytical methods, we aimed to comprehensively and accurately identify the genetic relationships between each pair of the nine metabolic diseases. A *p*‐value of less than 0.05 was considered indicative of significant genetic correlation or genetic overlap.

#### 2.2.2 Shared locus analysis

In the shared locus analysis, we concentrated on joint sets of paired traits that exhibited significant genetic correlation or overlap. For this, we employed PLACO methodology. PLACO is adept at identifying potentially pleiotropic SNVs between two traits. It operates by considering the composite null hypothesis, which posits that a variant is either not associated with any of the traits or is associated with just one of them.^21^ This approach is specifically designed to pinpoint pleiotropic loci between two traits.

To ensure the reliability of our findings and to avoid the influence of variants with extremely large effect sizes that could produce spurious signals, we excluded variants with a *Z*‐score squared (*Z* ^2^) greater than 80. We considered SNVs with a PLACO *p*‐value less than 5 × 10^−8^ as significant pleiotropic variants. This threshold was chosen to maintain a stringent criterion for identifying significant pleiotropic variants, thus enhancing the credibility of our analysis. For pleiotropic SNVs identified by PLACO, we further analysed them using FUMA to identify independent variants.^22^ FUMA can be used to describe genomic risk loci. We annotated variant functions using LD information from the 1000 Genomes Project Phase 3 reference panel of European populations and set the maximum *p*‐value of lead SNV to less than 5 × 10^−8^, and the maximum *p*‐value cutoff to less than 0.05. We characterized independent SNVs as those with an *r* ^2^ less than 0.6, and lead SNVs as those with an *r* ^2^ less than 0.1, within a 1 Mb range. To define genomic risk loci, we merged genomic regions if the physical distance between leading SNVs was less than 250 kb.^10^ This approach allowed us to systematically identify and characterize independent and lead SNVs, providing a detailed understanding of the genomic risk loci associated with the metabolic diseases studied.

For the identified potential pleiotropic loci, we conducted a Bayesian co‐localization analysis test. In this analysis, two hypotheses are particularly noteworthy: H3, which suggests that both traits are associated with different causal variants and H4, indicating that both traits are associated and share a causal variant. To perform this analysis, we utilized the default settings of the coloc.abf function. Specifically, the a priori probabilities of SNPs being associated with trait 1 and trait 2 were set at *p*1 = *p*2 = 1 × 10^−4^, and the a priori probability of SNPs being associated with both traits was set at *p*12 = 1 × 10^−5^. For the determination of significant co‐localization, we employed the posteriori probability of H4 (PP.H4). A PP.H4 value greater than 0.7 was used as the criterion for determining whether a locus should be considered as a co‐localized locus.^30^ This threshold was chosen to ensure a high level of confidence in identifying loci where both traits share a causal variant, thereby providing more robust and meaningful insights into the genetic overlap between the traits.

Through the analysis using the three methods mentioned above, we aimed to identify reliable SNVs between trait pairs with genetic correlations.

#### 2.2.3 Analysis of common biological mechanisms in pleiotropic gene loci

In this stage of our study, we focused on elucidating the common biological mechanisms of the Pleiotropic Gene Loci identified by the PLACO analysis. For this purpose, we annotated the pleiotropic SNVs using ANNOVAR.^31^ ANNOVAR is a tool that facilitates the functional annotation of genetic variants, allowing for a more detailed understanding of their potential biological implications.

We then calculated the Combined Annotation‐Dependent Depletion (CADD) and RegulomeDB scores for these variants. CADD scores are used to evaluate the potential deleteriousness of genetic variants. In our analysis, single‐nucleotide variants with CADD scores exceeding 12.37 were considered potentially deleterious.^32^ This threshold is based on established guidelines that suggest variants above this score are likely to have significant functional impacts.

Additionally, we used RegulomeDB scores to assess the regulatory potential of these SNPs. RegulomeDB provides a scoring system where lower scores indicate a stronger likelihood of the SNP being involved in gene regulation.^33^ By integrating these scores, we aimed to gain a comprehensive understanding of the functional impact and regulatory potential of the identified pleiotropic SNVs, thus shedding light on the common biological mechanisms underpinning the metabolic diseases studied.

In our study, we performed MAGMA at the gene level to identify candidate genes associated with pleiotropy. This analysis targeted genes located in or overlapping with the identified pleiotropic loci.^23^ We obtained MAGMA gene IDs and their locations from the MAGMA website (https://ctg.cncr.nl/software/magma), which uses NCBI build 37.3 and includes information on 19,427 protein‐coding genes. The significance threshold for MAGMA analysis was set at a Bonferroni‐corrected *p*‐value of less than 0.05 for each specific locus.

To delve deeper into the biological significance of these pleiotropic genes, we conducted a series of enrichment analyses, encompassing tissue‐specific and genomic enrichment. For phenotypic enrichment, we used data based on Mammalian Phenotype ontology from Mouse Genome Informatics.^34^ The deTS tissue‐specific enrichment method,^35^ drawing on reference data from the Genotypic Tissue Expression Project (GTEx) and the Encyclopedia of DNA Elements Project (ENCODE),^36^, ^37^ was employed to analyse tissue specificity. This involved using Fisher's exact test to compare our gene lists with each tissue‐specific gene set, aiding in the identification of tissue‐specific enrichment patterns.

Lastly, genomic enrichment analysis of the pleiotropic genes was conducted using the Molecular Signatures Database^38^ and the clusterProfiler software package.^39^ This step involved correcting for multiple tests in each category to uncover potential biological pathways. These comprehensive enrichment analyses allowed us to gain deeper insights into the functional aspects and biological pathways associated with the pleiotropic genes identified in our study.

#### 2.2.4 MR analysis

In our study, we conducted two‐way two‐sample MR (TSMR) analyses to explore potential causal relationships between pairwise traits among two sets of nine metabolic diseases, focusing on the concept of vertical pleiotropy. MR is different from genetic correlation studies like LDSC and HDL; it is a method that uses genetic variants as instrumental variables to assess causal relationships. MR uses genetic variants as instrumental variables, which are randomly allocated genetically, allowing for effective control of confounding factors, especially those difficult to control in traditional observational studies. Moreover, because genetic variants are determined at birth, MR can effectively reduce the impact of reverse causation, that is, the issue of the directionality of the causal relationship between exposure and outcome. The TSMR approach is founded on three critical assumptions: (I) the instrumental variable is closely related to the exposure risk, (II) the instrumental variable affects the outcome risk only through the exposure factor and (III) the instrumental variable is independent of confounding factors.^40^

For selecting instrumental variable SNPs, we set a significance threshold of *p* < 5 × 10^−8^. We used the PLINK clump method to assess linkage disequilibrium for each exposed SNP based on the 1000 Genomes European panel, with an *r* ^2^ < 0.01 (clustering distance = 5000 kb) indicating linkage equilibrium. The robustness of the IV was indicated by *f*‐statistics (calculated as *F* = *β* ^2^/SE^2^) exceeding 10.

Our MR analysis utilized several methods, including MR Egger, weighted median, inverse variance weighting (IVW), Wald ratio, simple mode and weighted mode. We primarily used IVW for analysis, considering a *p*‐value of <0.05 as significant.^11^, ^41^ Cochran's *Q*‐statistic was employed to assess heterogeneity among individual SNPs, with a fixed‐effects model applied in cases of no significant heterogeneity (*p* < 0.05). If significant heterogeneity was detected, causal relationships were interpreted with caution.

We also conducted sensitivity analyses to verify the robustness of our results. The MR‐Egger and MR‐PRESSO methods were utilized to check for multinomiality. The MR‐Egger regression intercept measured directional pleiotropy, and MR‐PRESSO was used to enhance pleiotropy detection.^42^ Additionally, the STEIGER test was performed to determine the direction of causality, and leave‐one‐out sensitivity analyses assessed the impact of individual SNPs on MR results.

#### 2.2.5 Mediation MR analysis

We used the positive results from MR for T2D GWAS as a mediator variable and applied MR analysis to determine the causal relationship between hypothyroidism/myxoedema and hypoglycaemia through IVs. In MR analysis, *β*0−*β*1 × *β*2 is used to assess the direct effect of exposure on the outcome,^43^ where *β*0 measures the causal effect of exposure on the outcome, *β*1 quantifies the causal effect of exposure on the mediator variable, *β*2 represents the causal effect of the mediator variable on the outcome, and *β*1 × *β*2 represents the mediating effect of exposure on the outcome.^44^

## 3 RESULTS

### 3.1 Genetic correlations and genetic overlap among common metabolic diseases

Among the 36 trait pairs formed from 9 common metabolic diseases, LDSC identified 14 pairs with significant genetic correlations, while HDL identified 11 pairs with significant genetic correlations. Additionally, we identified 22 trait pairs with significant genetic overlap through GPA. Notably, the trait pairs T2D‐hypoglycaemia (P‐LDSC = 9.55E‐40; P‐HLD = 0.007), T2D‐osteoporosis (P‐LDSC = 0.002; P‐HLD = 3.336E‐08), T2D‐gout (P‐LDSC = 1.400E‐10; P‐HLD = 8.538E‐07), T2D‐hypothyroidism/myxoedema (P‐LDSC = 3.330E‐05; P‐HLD = 2.227E‐05), T2D‐hypertension (P‐LDSC = 0.008; P‐HLD = 0.002), hypoglycaemia‐hypothyroidism/myxoedema (P‐LDSC = 7.870E‐6; P‐HLD = 0.011), gout‐hypothyroidism/myxoedema (P‐LDSC = 0.002; P‐HLD = 0.002) and hypothyroidism/myxoedema‐hyperthyroidism/thyrotoxicosis (P‐LDSC = 0.001; P‐HLD = 5.532E‐017) were identified to have significant genetic correlations in both LDSC and HDL, and they all exhibited significant genetic overlap in GPA (P‐GPA < 0.05) (Table S2). Ultimately, we conducted further analyses on these eight trait pairs.

Additionally, it is noteworthy that the trait pairs T2D‐hypothyroidism/myxoedema‐hypoglycaemia and T2D‐hypothyroidism/myxoedema‐gout showed both significant genetic correlations and overlaps. Although hypoglycaemia‐gout did not have a significant genetic correlation, it exhibited significant genetic overlap.

### 3.2 Shared loci between common metabolic diseases

In our analysis using the PLACO, we identified a significant number of SNVs. Specifically, 18,072 SNVs were found across the eight traits mentioned earlier. Subsequent analysis using FUMA led to the identification of 559 independent genomic risk loci, which spanned 182 independent chromosomal regions. This finding underscores the extensive polyvalent nature of these loci.

**FIGURE 2:**
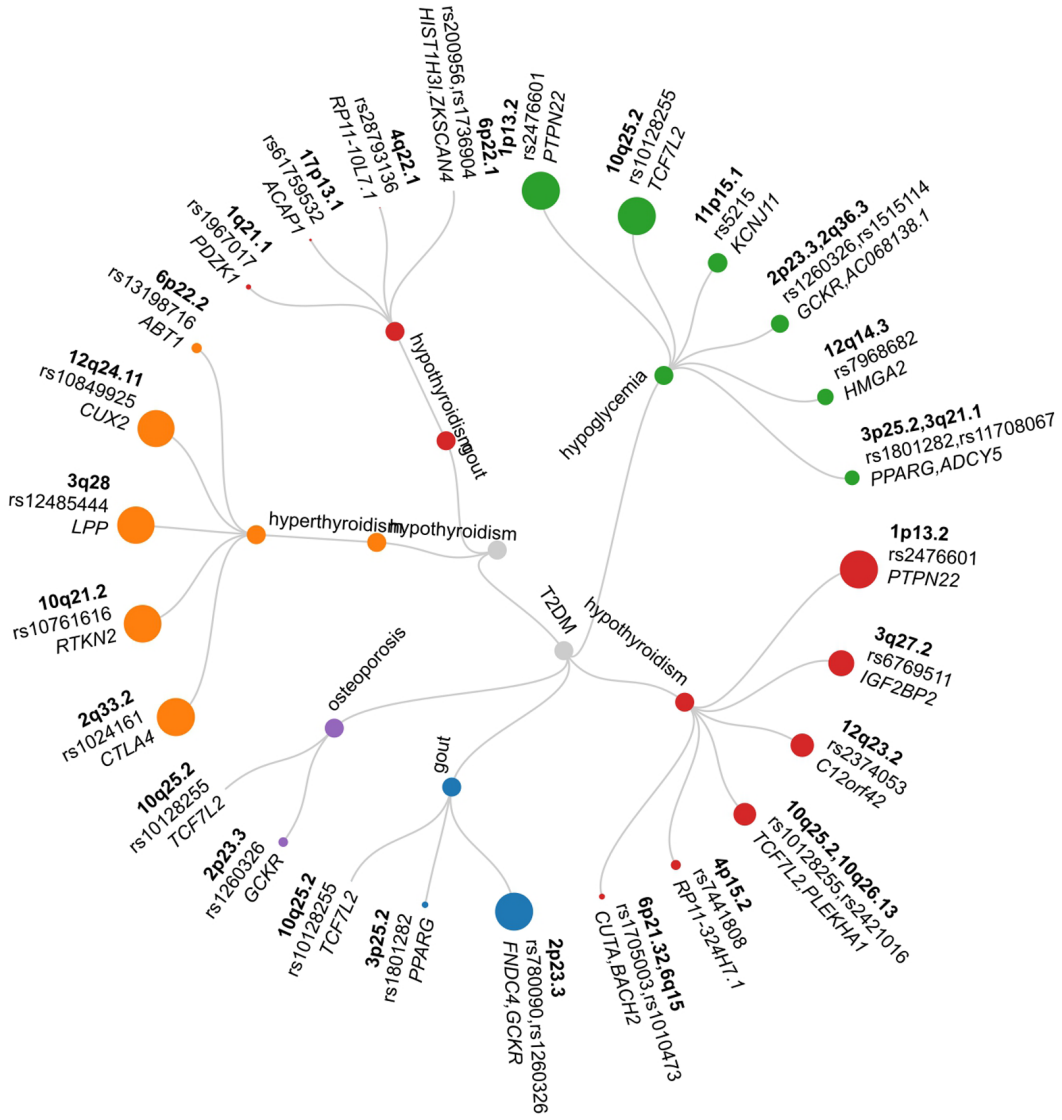
Genetic correlation, overlap and locus information among eight positive trait pairs.

Among these loci, we noticed several common pleiotropic regions shared across multiple paired traits. For instance, the 4p16.1 region was present in six different trait pairs. Similarly, the regions 10q25.2, 3q27.2 and 6p22.3 were each associated with five trait pairs, while 1p13.2, 2q32.2, 3p25.2 and 6p22.2 were each linked to four trait pairs. This widespread association indicates a broad spectrum of pleiotropy across these loci (Figure 2).

Through Bayesian co‐localization analysis, we found that 29 potential pleiotropic loci, constituting 15.5% of the total, had a PP.H4 value greater than 0.7. This result highlights their potential significance in pleiotropic genetic relationships. For example, the 1p13.2 locus remained significant across four trait pairs: T2D and hypoglycaemia, T2D and hypothyroidism/myxoedema, hypoglycaemia and hypothyroidism/myxoedema and hypothyroidism/myxoedema and hyperthyroidism/thyrotoxicosis. Additionally, the 12q24.11 and 12q24.12 loci were found to co‐localize in two trait pairs involving gout and thyroid‐related diseases, indicating their relevance in thyroid metabolism.

Notably, the 1p13.2 locus showed co‐localization between the three traits of T2D, hypothyroidism/myxoedema and hypoglycaemia, suggesting a pivotal role in the genetic interplay among these conditions (Tables S3 and S4).

### 3.3 Analysis of common biological mechanisms of pleiotropic loci

In the analysis of the common biological mechanisms underlying pleiotropic loci, we used ANNOVAR to annotate the identified loci. This detailed annotation revealed a diverse distribution of SNVs across different genomic regions: 283 SNVs were exonic variants, 9623 SNVs were intergenic variants, 12,587 SNVs were intronic variants, 417 SNVs were ncRNA_exonic variants, 2274 SNVs were ncRNA_intronic variants and 2 SNVs were ncRNA_splicing variants. Out of these, 32 SNVs (comprising 25 unique SNVs) had high CADD scores (>12.37), indicating their potential deleterious impact.

A notable finding was the rs2476601 (*PTPN22*) variant within the 1p13.2 region, identified in Section 3.2. This variant, which is exonic, exhibited a high CADD score in both T2D‐hypoglycaemia and T2D‐hypothyroidism/myxoedema. It is a missense variant previously associated with insulin‐dependent diabetes mellitus (*p* = 2 × 10^−6^)^45^ and autoimmune diseases, including thyroid dysfunction (*p* = 3 × 10^−13^).^46^

Another significant variant is rs10849925 (*CUX2*) within the 12q24.11 region. This intron variant, which also has a high CADD score, is associated with hypothyroidism/myxoedema‐hyperthyroidism/thyrotoxicosis. It has been linked to various conditions, including elevated triglyceride levels (*p* = 5 × 10^−13^),^47^ serum uric acid level (*p* = 4 × 10^−20^),^48^ type 1 diabetes mellitus (*p* = 1 × 10^−16^)^49^ and hypertension (*p* = 7 × 10^−12^).^50^

These findings suggest that these SNVs, particularly the exonic variant rs2476601 in the 1p13.2 region, are potentially deleterious. The co‐localization of rs2476601 between T2D, hypothyroidism/myxoedema and hypoglycaemia, along with its high CADD score, implies a significant role in the development of these diseases, potentially through the functional regulation of *PTPN22* gene expression. Further investigation revealed chromatin loop interactions between rs2476601 and the promoter/enhancer of *PTPN22*, using data from GeneHancer in GeneCards (GH01J113869) (Tables S3 and S4).

MAGMA analysis revealed a total of 623 potentially pleiotropic genes across the eight trait pairs, encompassing 365 unique genes. A noteworthy finding was the detection of the TAP2 gene in five of these trait pairs. Significantly, TAP2 was also identified in all three traits within the T2D‐hypothyroidism/myxoedema‐hypoglycaemia relationship (Table S5). Additionally, *PTPN22*, a gene of particular interest in our study, was found in both the T2D‐hypothyroidism/myxoedema and T2D‐hypoglycaemia trait pairs.

**FIGURE 3:**
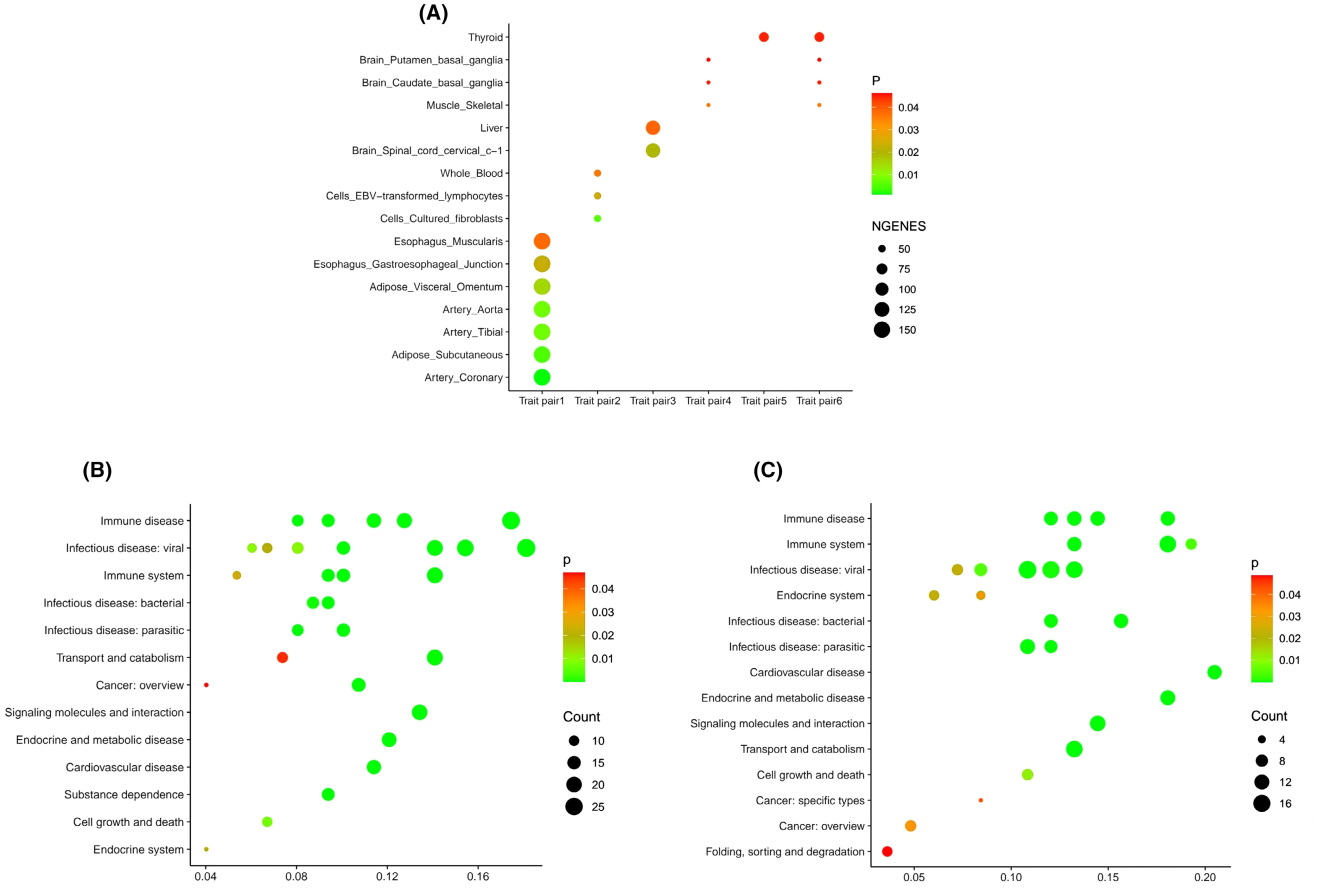
Enrichment analysis (A), tissue enrichment among positive trait pairs, where Trait pair 1 represents gout‐hypothyroidism; Trait pair 2 represents T2D‐hypothyroidism; Trait pair 3 represents hypothyroidism‐hyperthyroidism; Trait pair 4 represents hypoglycaemia‐hypothyroidism; Trait pair 5 represents T2D‐hypoglycaemia; Trait pair 6 represents T2D‐hypothyroidism/myxoedema‐hypoglycaemia. (B) Gene enrichment pathways among the eight positive trait pairs. (C) Gene enrichment pathways among hypothyroidism, T2D and hypoglycaemia.

The tissue‐specific enrichment analysis, conducted using the deTS method based on the GTEx and the ENCODE reference panels, yielded insightful results. These pleiotropic genes showed predominant enrichment in several tissues: the adrenal gland (*p* = 0.025), EBV‐transformed lymphocytes (*p* = 0.022), kidney cortex (*p* = 0.020) and spleen (*p* = 0.018). Moreover, focusing on the three traits of interest—T2D, hypothyroidism/myxoedema and hypoglycaemia—these pleiotropic genes were predominantly enriched in the thyroid (*p* = 0.045), the brain's caudate basal ganglia (*p* = 0.045), the brain's putamen basal ganglia (*p* = 0.046) and the spleen (*p* = 0.036) (Table S6 and Figure 3A).

This analysis provides valuable insights into the potential biological mechanisms underlying the genetic associations in these metabolic diseases, highlighting the role of specific genes and tissue types.

The gene set enrichment analysis results provided a comprehensive view of the pathways involving pleiotropic genes, spanning a wide range of biological dimensions. Notably, these pathways predominantly include endocrine and metabolic disease (*p*.adjust = 3.11E−18), immune disease (*p*.adjust = 3.12E−18), transport and catabolism (*p*.adjust = 3.32E−11) and infectious disease (*p*.adjust = 4.97E−11), among others (Table S7 and Figure 3B). In the case of the T2D‐hypothyroidism/myxoedema‐hypoglycaemia triple trait relationship, genes were significantly enriched in the immune system (*p*.adjust = 3.00E−13), endocrine and metabolic disease (*p*.adjust = 9.78E−13) and infectious disease (*p*.adjust = 5.14E−10) (Figure 3C).

These analyses underscore the potential role of the immune system in metabolic diseases. The immune system may exert its influence through precise modulation of hormones or inflammatory factors. Moreover, the association of immune system activation with inflammatory responses and various infections offers a compelling perspective to explore the link between chronic inflammation and multiple metabolic and endocrine diseases.

Based on these findings, we hypothesize that the immune system may be intricately linked to the development of metabolic and endocrine diseases, particularly through regulating inflammatory responses and responding to infections. Chronic inflammation, for instance, is a well‐acknowledged factor in the development of T2D and other metabolic disorders. In these diseases, immune responses might influence cell signalling, metabolic regulation and hormonal balance. Further research in this area could provide vital insights for identifying new therapeutic targets and strategies.

To further elucidate these complex interactions, we conducted MR analyses of inflammatory protein factors and metabolic diseases. This next step is aimed at testing the aforementioned hypotheses and uncovering the underlying biological mechanisms.

### 3.4 MR and mediation MR analysis

**FIGURE 4:**
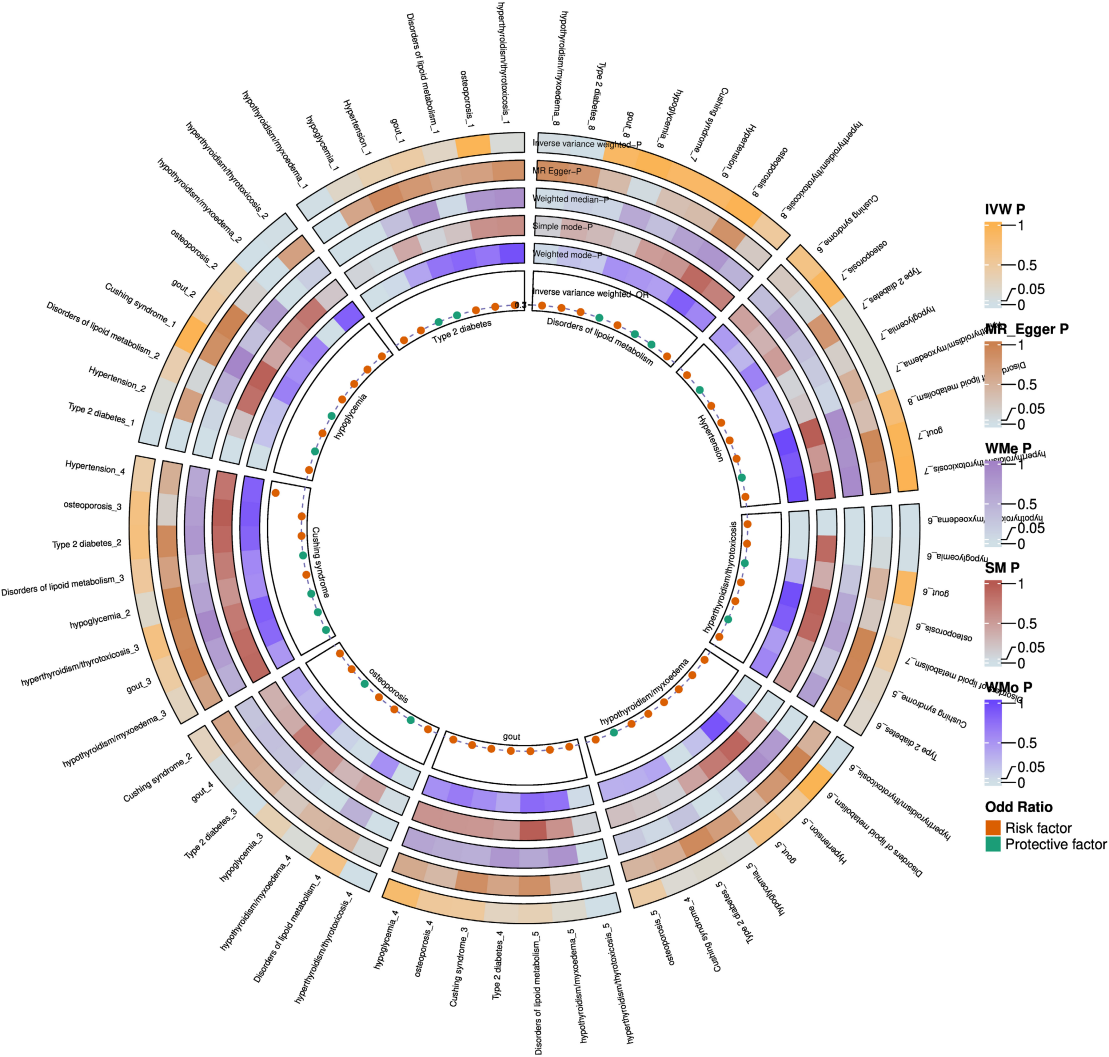
Forest plot of bidirectional MR in metabolic diseases. The *p*‐values of the five methods of TSMR for different shape pairs are shown, the innermost and outermost layers in the figure are different shape pairs, the five stripes represent the five methods respectively, which one represents what is described in detail on the right side of the picture, and the meanings of the different colours represented are also shown on the right side of the picture, the red dots in the inner measurements of the stripes represent the risk factor, and the green dots represent the protective factor.

First, we conducted bidirectional TSMR analysis on the 36 trait pairs formed by the 9 common metabolic diseases. The results indicated significant causal relationships for 10 pairs (Table S8), with no observed heterogeneity or horizontal pleiotropy. Notably, there were significant positive causal relationships between hypothyroidism/myxoedema and T2D (*β* = 0.856; FDR = 0.018), T2D and hypoglycaemia (*β* = 0.528; FDR = 8.33E‐16) and hypothyroidism/myxoedema and hypoglycaemia (*β* = 4.359; FDR = 2.62E‐10). These findings suggest that hypothyroidism/myxoedema may increase the risk of T2D, which in turn may increase the risk of hypoglycaemia. Based on this, we discovered that T2D likely mediates the causal relationship between hypothyroidism/myxoedema and hypoglycaemia (mediating effect = 3.731; direct effect = 0.628) (Table S8 and Figure 4).

Since we identified that genes associated with the relationship among T2D, hypothyroidism/myxoedema and hypoglycaemia were mainly enriched in immune‐related pathways, we performed bidirectional MR analysis on 91 inflammatory protein factors and these three metabolic diseases. The analysis revealed that *CXCL10* plays a critical role in the vertical genetic relationships among these traits. Specifically, the results were as follows: *CXCL10*‐hypothyroidism/myxoedema (*β* = 0.016, *p* = 0.045), hypothyroidism/myxoedema‐*CXCL10* (*β* = 0.971, *p* = 0.0006), *CXCL10*‐T2D (*β* = 0.079, *p* = 0.036), T2D‐*CXCL10* (*β* = −0.038, *p* = 0.017) and *CXCL10*‐hypoglycaemia (*β* = 0.307, *p* = 0.029) (Table S9 and Figure 4). These findings suggest that *CXCL10* plays a complex and crucial role in the causal relationship among hypothyroidism/myxoedema, T2D and hypoglycaemia.

## 4 DISCUSSION

The primary objective of our study was to explore the genetic and biological connections among nine common metabolic diseases. Our findings revealed significant genome‐wide genetic correlations and overlaps among some of these disorders. A focal point of our research was the genetic relationships among the triad of hypothyroidism, type 2 diabetes mellitus and hypoglycaemia.

Existing research suggests a bidirectional relationship between hypothyroidism and T2D. On one side, hypothyroidism can increase the risk of developing T2D. This is potentially due to hypothyroidism's impact on the metabolic rate, which may impair glucose regulation. It affects insulin sensitivity and secretion and can lead to weight gain—a recognized risk factor for T2D. Conversely, individuals with T2D may have an elevated risk of developing hypothyroidism, likely due to insulin resistance and metabolic dysregulation.^51^

Furthermore, our analysis identified numerous shared SNVs and genomic risk loci linked to these conditions. Notably, the rs2476601 variant emerged as a significant focus in our study. This missense variant, located at the promoter region of the *PTPN22* gene, appears to play a crucial role in the genetic association between type 2 diabetes mellitus, hypothyroidism/myxoedema and hypoglycaemia.

The implications of these findings are multifold. They not only enhance our understanding of the genetic basis of these metabolic diseases but also potentially open new avenues for targeted therapeutic and preventive strategies. By elucidating these genetic interconnections, our study contributes to the broader narrative of how metabolic diseases are interlinked, paving the way for further research in this vital area of medical science.

*PTPN22* encodes a protein belonging to the non‐receptor class 4 subfamily of the tyrosine phosphatase family. It functions as an intracellular phosphatase, primarily lymphoid‐specific and plays a critical role as a negative regulator of T‐cell receptor (TCR) signalling. The protein achieves this regulation through the direct dephosphorylation of several key signalling molecules, including the Src family kinases LCK and FYN, ITAM of the TCRz/CD3 complex, ZAP70, VAV, VCP and potentially CBL.^52^, ^53^ Notably, it dephosphorylates LCK, ZAP70 and SKAP2 at the activated ‘Tyr‐394’ residue.^52^, ^54^ Moreover, *PTPN22* positively influences Toll‐like receptor‐induced type 1 interferon production^55^ and is involved in promoting type 1 interferon‐mediated antiviral responses. It also plays a role in regulating NOD2‐induced pro‐inflammatory cytokine secretion and autophagy.^56^

Research on *PTPN22* has predominantly been centred on its association with rheumatoid arthritis, type 1 diabetes mellitus and autoimmune thyroid diseases. However, there exists a gap in fully understanding its molecular genetic role in type 2 diabetes mellitus, particularly its immunological relationship with HT and hypoglycaemia.

Significant associations have been reported between *PTPN22* 1858T and Graves' thyroiditis by Smyth et al.,^57^ with a review by Siminovitch^58^ further reinforcing the evidence of *PTPN22*'s involvement in susceptibility to both systemic and organ‐specific autoimmune diseases. Kawasaki et al.^26^ conducted genotyping on 1698 Asians, including 732 individuals with type 1 diabetes and 141 with autoimmune thyroid disease, finding that all possessed the wild‐type *PTPN22* 1858C/C genotype.

Differences in the distribution of *PTPN22* copies between T2D patients and healthy controls have also been found. Studies have shown that increased copy number of the *PTPN22* gene is an important risk factor for T2D, and there is also a positive correlation between *PTPN22* copy number variation and fasting blood glucose and glycosylated haemoglobin levels in patients with T2D. These findings suggest that the *PTPN22* gene, especially its copy number variant, may be associated with dysregulated glucose metabolism and predispose individuals to T2D.^59^

Our enrichment analysis highlighted that the pathways involving pleiotropic genes extend across various biological dimensions, particularly emphasizing the potential pivotal role of the immune system in metabolic diseases such as T2D, hypoglycaemia and thyroid disorders.

In T2D, it has been established that immune pathways, notably chronic low‐grade inflammation, play a crucial role. This inflammation is often attributed to factors like obesity, changes in adipose tissue and metabolic imbalances.^51^, ^60^, ^61^ Commonly, T2D patients exhibit elevated levels of certain inflammatory markers, including C‐reactive protein, tumour necrosis factor‐α and interleukin‐6. Moreover, there is a significant correlation between the infiltration of immune cells, especially macrophages, into adipose tissue and the onset of insulin resistance. These immune cells release inflammatory mediators that can disrupt normal insulin signalling processes. Additionally, obesity, particularly abdominal obesity, is linked with increased activation of immune cells and inflammatory mediators, thereby intensifying insulin resistance.^62^, ^63^

Although insulin resistance is a primary feature of T2D, evidence suggests that autoimmune responses might also contribute to the disease in some patients. For instance, autoantibodies against islet cells have been detected in a subset of T2D patients.^64^, ^65^ Furthermore, changes in the gut microbiota, or dysbiosis, can influence the host's immune response and are associated with the development of T2D. The gut flora plays a vital role in regulating metabolism, the inflammatory response and immune function, all of which are closely linked to the host's overall health status.^66^, ^67^

The link between hypothyroidism, particularly HT, and the immune system is indeed strong. HT is an autoimmune condition where the immune system erroneously attacks the thyroid gland, causing chronic inflammation and destruction of thyroid cells. This autoimmune response is characterized by the presence of specific autoantibodies against thyroid components, namely anti‐thyroid peroxidase antibodies and anti‐thyroglobulin antibodies, commonly found in patients with HT.^68^, ^69^

Lymphocyte infiltration and other immune cells in the thyroid tissue are typical findings in HT. The cytokines and chemical signalling substances released by these immune cells can intensify the inflammatory response and further impair thyroid function. The development of HT is also linked to genetic predispositions, especially variations in certain HLA (human leukocyte antigen) genotypes and other immunomodulatory genes, which are associated with a heightened risk of developing the disease.

Moreover, individuals with HT may have an increased susceptibility to other autoimmune diseases, such as type 1 diabetes, rheumatoid arthritis and lupus erythematosus.^70^, ^71^, ^72^ This increased susceptibility suggests a broadened autoimmune profile, where the immune system's dysregulation extends beyond the thyroid, potentially affecting other bodily systems and organs. Understanding these connections can provide critical insights into the pathophysiology of autoimmune diseases and guide the development of more effective diagnostic and therapeutic strategies.

Hypoglycaemia is often regarded as a side effect of diabetes treatment, particularly when using insulin or certain types of oral hypoglycaemic agents. The direct link between hypoglycaemia and the immune system has been relatively less studied. In type 1 diabetes, hypoglycaemia is common as it is an autoimmune disease in which the immune system attacks and destroys the insulin‐producing beta cells in the pancreas. For T2D patients, although the primary issue is insulin resistance, the use of insulin or certain oral hypoglycaemic drugs can also lead to hypoglycaemia. Hypoglycaemic events may activate the body's stress response, including increased secretion of adrenaline, cortisol and other stress hormones, which can regulate the function of the immune system. Repeated hypoglycaemic events may impact the immune system, particularly in patients with chronic diabetes.^73^, ^74^, ^75^ Diabetic patients, especially those with poor glycaemic control, may have a higher risk of infection, but the direct relationship with hypoglycaemia is not yet clear. In some cases, the chronic low‐grade inflammatory state of diabetic patients may be associated with the occurrence of hypoglycaemic events, but research in this area is relatively scarce.

Our utilization of MR analysis not only further confirmed the genetic relationship among hypothyroidism, T2D and hypoglycaemia but also verified the potential key role of immune‐related inflammatory protein factors, such as CXCL10, in the development of these diseases. These findings not only enhance our understanding of the genetic basis of metabolic diseases but also provide new clues for future therapeutic strategies. In the treatment strategies for T2D, some anti‐inflammatory drugs have shown potential benefits, and drugs that reduce inflammatory responses may help improve insulin sensitivity.^76^, ^77^, ^78^, ^79^

This study has some acknowledged limitations. The GWAS data used were driven by individuals of European descent, necessitating the exploration of other ethnic groups to investigate if our results can be replicated in a more diverse ancestry. Additionally, due to the nature of GWAS, it captures only common variants associated with diseases, often failing to reveal information about rare or structural variants. Furthermore, genes identified through GWAS associations may have different biological functions, and explaining how these functions collectively regulate the relationships between different metabolic diseases can be complex. Biological interpretations require further experimental validation and functional studies to confirm the exact roles of these genes. Lastly, to avoid the substantial linkage disequilibrium in the MHC region, this area was excluded from the analysis at the outset. However, given the strong correlation between immune diseases and the MHC region,^80^ this may lead to the omission of some results.

## 5 CONCLUSION

This study marks a significant advancement in the field of metabolic disease research by elucidating the genetic relationships among hypothyroidism, T2D and hypoglycaemia, with a particular focus on the rs2476601 variant and the role of the immune system, especially the inflammatory protein *CXCL10*. The results not only deepen our understanding of the genetic basis of these diseases but also lay a foundational framework for future investigations and therapeutic developments.

## AUTHOR CONTRIBUTIONS

Jing Shen, Julong Pan, Gang Yu, Hui Cai, Hua Xu, Hanfei Yan and Yu Feng wrote the main manuscript text, and all authors reviewed the manuscript.

## FUNDING INFORMATION

This work was supported by Suzhou Science and Technology Bureau Medical Application Basic Research Medical Innovation Application Research Program (SKY2023101).This work was also supported by Wujiang District Science and Education Revitalization Health Project (WWK202221).

## CONFLICT OF INTEREST STATEMENT

The authors declare that they have no competing interests.

## Supporting information


Tables S1–S9.

## Data Availability

In this study, all GWAS data were sourced from three primary databases: IEU, FinnGen and UK Biobank. These databases provided comprehensive information on nine common metabolic diseases: type 2 diabetes (ebi‐a‐GCST006867), hypertension (ukb‐d‐I9_HYPTENS), disorders of lipoid metabolism (ukb‐e‐272_CSA), hyperthyroidism/thyrotoxicosis (ukb‐b‐20289), hypothyroidism/myxoedema (ukb‐b‐19732), osteoporosis (ukb‐b‐12141), gout (ukb‐b‐13251), diabetic hypoglycaemia (finn‐b‐DM_HYPOGLYC) and Cushing's syndrome (finn‐b‐E4_CUSHING). Additionally, data on 91 circulating inflammatory proteins were obtained from GWAS measurements using the Olink Target Inflammation Panel. This data was collected across 11 cohorts, involving a total of 14,824 participants of European ancestry. Detailed descriptions of the samples are available in key publications, and it's important to note that all GWAS data used in our analysis pertained to populations of European ancestry.
