# Supplementary material for: Genetic interactions and pleiotropy in metabolic diseases: Insights from a comprehensive GWAS analysis
Source: J Cell Mol Med. 2024 Sep 5;28(17):e70045. doi: 10.1111/jcmm.70045 (PMC11377178; doi:10.1111/jcmm.70045)
Supplement: Supplementary file 1 — Tables S1–S9. [file JCMM-28-e70045-s001.pdf]

**content**	**Supplementary table**
Data Sources	S1
Genome-Wide Genetic Correlations and Genetic Overlap	S2
Shared Loci Between Common Metabolic Diseases	S3
Analysis of FUMA	S4
Analysis of MAGMA	S5
Analysis of tissue-specific enrichment analysis	S6
analysis of pathways	S7
Mendelian Randomization Analysis of nine metabolic disorders and Mediation MR	S8
Mendelian Randomization Analysis of the interaction of 91 inflammatory protein factors with these three metabolic disorders	S9

Data Sources

TRIT	PMID/Source	Samplesize	Ancestry
91 circulating inflammatory proteins	37563310	14824	EUR
Type2 diabetes	ebi-a-GCST006867	655666	EUR
Hypertension	ukb-d-I9_HYPTENS	361194	EUR
Disorders of lipoid metabolism	ukb-e-272_CSA	8876	EUR
hyperthyroidism/thyrotoxicosis	ukb-b-20289	462933	EUR
hypothyroidism/myxoedema	ukb-b-19732	462933	EUR
osteoporosis	ukb-b-12141	462933	EUR
gout	ukb-b-13251	462933	EUR
Diabetic hypoglycemia	finn-b-DM_HYPOGLYC	162201	EUR
Cushing syndrome	finn-b-E4_CUSHING	211123	EUR

91 details of circulating inflammatory proteins

TRIT	PMID/Source	Discovery sample number	Replication sample
C-X-C motif chemokine 6 levels	GCST90274783	• 14744 European	• 1585 European, NR
Adenosine Deaminase levels	GCST90274759	• 14736 European	• 1585 European, NR
Delta and Notch-like epidermal growth factor-related receptor	GCST90274785	• 14735 European	• 1585 European, NR
Axin-1 levels	GCST90274761	• 11793 European	• 1585 European, NR
Fibroblast growth factor 19 levels	GCST90274787	• 14744 European	• 1585 European, NR
Fibroblast growth factor 21 levels	GCST90274788	• 14743 European	• 1585 European, NR
Eotaxin levels	GCST90274764	• 14734 European	• 1585 European, NR
C-C motif chemokine 19 levels	GCST90274765	• 14736 European	• 1585 European, NR
C-C motif chemokine 20 levels	GCST90274766	• 14736 European	• 1585 European, NR
C-C motif chemokine 23 levels	GCST90274767	• 14736 European	• 1585 European, NR
C-C motif chemokine 25 levels	GCST90274768	• 14736 European	• 1585 European, NR
Interferon gamma levels	GCST90274794	• 11793 European	• 1585 European, NR
Interleukin-10 levels	GCST90274795	• 14744 European	• 1585 European, NR

Natural killer cell receptor 2B4 levels	GCST90274771	• 14735 European	• 1585 European, NR
CD40L receptor levels	GCST90274772	• 14736 European	• 1585 European, NR
T-cell surface glycoprotein CD5 levels	GCST90274773	• 14735 European	• 1585 European, NR
T-cell surface glycoprotein CD6 isoform levels	GCST90274774	• 14735 European	• 1585 European, NR
CUB domain-containing protein 1 levels	GCST90274775	• 14734 European	• 1585 European, NR
Interleukin-17A levels	GCST90274801	• 11784 European	• 1585 European, NR
Cystatin D levels	GCST90274777	• 14736 European	• 1585 European, NR
Fractalkine levels	GCST90274778	• 14743 European	• 1585 European, NR
interleukin-18 receptor 1 levels	GCST90274804	• 14743 European	• 1585 European, NR
C-X-C motif chemokine 10 levels	GCST90274780	• 14744 European	• 1585 European, NR
C-X-C motif chemokine 11 levels	GCST90274781	• 14736 European	• 1585 European, NR
Eukaryotic translation initiation factor 4E-binding protein 1 levels	GCST90274758	• 14736 European	• 1585 European, NR
C-X-C motif chemokine 9 levels	GCST90274784	• 14735 European	• 1585 European, NR
Artemin levels	GCST90274760	• 11778 European	• 1585 European, NR
Protein S100-A12 levels	GCST90274786	• 14743 European	• 1585 European, NR
beta-nerve growth factor levels	GCST90274762	• 14743 European	• 1585 European, NR
Caspase 8 levels	GCST90274763	• 14744 European	• 1585 European, NR
Fibroblast growth factor 23 levels	GCST90274789	• 14735 European	• 1585 European, NR
Fibroblast growth factor 5 levels	GCST90274790	• 11789 European	• 1585 European, NR
Fms-related tyrosine kinase 3 ligand levels	GCST90274791	• 14734 European	• 1585 European, NR
Glial cell line-derived neurotrophic factor levels	GCST90274792	• 14736 European	• 1585 European, NR
Hepatocyte growth factor levels	GCST90274793	• 14734 European	• 1585 European, NR
C-C motif chemokine 28 levels	GCST90274769	• 14734 European	• 1585 European, NR
C-C motif chemokine 4 levels	GCST90274770	• 14744 European	• 1585 European, NR
Interleukin-10 receptor subunit alpha levels	GCST90274796	• 11793 European	• 1585 European, NR
Interleukin-10 receptor subunit beta levels	GCST90274797	• 14734 European	• 1585 European, NR
Interleukin-12 subunit beta levels	GCST90274798	• 14735 European	• 1585 European, NR
Interleukin-13 levels	GCST90274799	• 11792 European	• 1585 European, NR
Interleukin-15 receptor subunit alpha levels	GCST90274800	• 11792 European	• 1585 European, NR

Macrophage colony-stimulating factor 1 levels	GCST90274776	• 14734 European	• 1585 European, NR
Interleukin-17C levels	GCST90274802	• 11793 European	• 1585 European, NR
Interleukin-18 levels	GCST90274803	• 14744 European	• 1585 European, NR
C-X-C motif chemokine 1 levels	GCST90274779	• 14736 European	• 1585 European, NR
Interleukin-1-alpha levels	GCST90274805	• 11788 European	• 1585 European, NR
Interleukin-2 levels	GCST90274806	• 11789 European	• 1585 European, NR
Interleukin-20 levels	GCST90274807	• 11784 European	• 1585 European, NR
C-X-C motif chemokine 5 levels	GCST90274782	• 14736 European	• 1585 European, NR
Interleukin-20 receptor subunit alpha levels	GCST90274808	• 11792 European	• 1585 European, NR
Interleukin-22 receptor subunit alpha-1 levels	GCST90274809	• 11793 European	• 1585 European, NR
Interleukin-24 levels	GCST90274810	• 11785 European	• 1585 European, NR
Interleukin-2 receptor subunit beta levels	GCST90274811	• 11792 European	• 1585 European, NR
Interleukin-33 levels	GCST90274812	• 11793 European	• 1585 European, NR
Interleukin-4 levels	GCST90274813	• 11793 European	• 1585 European, NR
Interleukin-5 levels	GCST90274814	• 11792 European	• 1585 European, NR
Interleukin-6 levels	GCST90274815	• 14743 European	• 1585 European, NR
Interleukin-7 levels	GCST90274816	• 14736 European	• 1585 European, NR
Interleukin-8 levels	GCST90274817	• 14744 European	• 1585 European, NR
Latency-associated peptide transforming growth factor beta 1 levels	GCST90274818	• 14736 European	• 1585 European, NR
Leukemia inhibitory factor levels	GCST90274819	• 11793 European	• 1585 European, NR
Leukemia inhibitory factor receptor levels	GCST90274820	• 11784 European	• 1585 European, NR
Monocyte chemoattractant protein-1 levels	GCST90274821	• 14733 European	• 1585 European, NR
Monocyte chemoattractant protein 2 levels	GCST90274822	• 14736 European	• 1585 European, NR
Monocyte chemoattractant protein-3 levels	GCST90274823	• 11783 European	• 1585 European, NR
Monocyte chemoattractant protein-4 levels	GCST90274824	• 14736 European	• 1585 European, NR
Macrophage inflammatory protein 1a levels	GCST90274825	• 14743 European	• 1585 European, NR
Matrix metalloproteinase-1 levels	GCST90274826	• 14744 European	• 1585 European, NR
Matrix metalloproteinase-10 levels	GCST90274827	• 14744 European	• 1585 European, NR
Neurturin levels	GCST90274828	• 11791 European	• 1585 European, NR

Neurotrophin-3 levels	GCST90274829	• 14744 European	• 1585 European, NR
Osteoprotegerin levels	GCST90274830	• 14733 European	• 1585 European, NR
Oncostatin-M levels	GCST90274831	• 14736 European	• 1585 European, NR
Programmed cell death 1 ligand 1 levels	GCST90274832	• 14736 European	• 1585 European, NR
Stem cell factor levels	GCST90274833	• 14736 European	• 1585 European, NR
SIR2-like protein 2 levels	GCST90274834	• 14736 European	• 1585 European, NR
Signaling lymphocytic activation molecule levels	GCST90274835	• 14734 European	• 1585 European, NR
Sulfotransferase 1A1 levels	GCST90274836	• 11793 European	• 1585 European, NR
STAM binding protein levels	GCST90274837	• 14736 European	• 1585 European, NR
Transforming growth factor-alpha levels	GCST90274838	• 14733 European	• 1585 European, NR
Tumor necrosis factor levels	GCST90274839	• 11785 European	• 1585 European, NR
TNF-beta levels	GCST90274840	• 11792 European	• 1585 European, NR
Tumor necrosis factor receptor superfamily member 9 levels	GCST90274841	• 11784 European	• 1585 European, NR
Tumor necrosis factor ligand superfamily member 14 levels	GCST90274842	• 11793 European	• 1585 European, NR
TNF-related apoptosis-inducing ligand levels	GCST90274843	• 14735 European	• 1585 European, NR
TNF-related activation-induced cytokine levels	GCST90274844	• 14736 European	• 1585 European, NR
Thymic stromal lymphopoietin levels	GCST90274845	• 11793 European	• 1585 European, NR
Tumor necrosis factor ligand superfamily member 12 levels	GCST90274846	• 14736 European	• 1585 European, NR
Urokinase-type plasminogen activator levels	GCST90274847	• 14734 European	• 1585 European, NR
Vascular endothelial growth factor A levels	GCST90274848	• 14744 European	• 1585 European, NR

Genome-Wide Genetic Correlations and Genetic Overlap

				LDSC			HDL				GPA	MR	Reverse MR
**Source**	**Source**	**TRIT**	**TRIT**	**rg**	**rg_se**	**rg_p**	**rg**	**rg_se**	**rg_p**	**statistics**	**pvalue**	**FDR**	**FDR**
ebi-a-GCST006867	finngen_R9_DM_HYPOGLYC	Type 2 diabetes	hypoglycemia	0.628	0.0476	9.55E-40	1.029	0.374	0.00697	6657.23518	1E-300	8.32955E-16	0.263417
ebi-a-GCST006867	finngen_R9_E4_CUSHING	Type 2 diabetes	Cushing syndrome	0.462	0.294	0.116	inf	na	na	0.02987346	0.862778	0.913238072	0.416505
ebi-a-GCST006867	ukb-b-12141	Type 2 diabetes	osteoporosis	0.167	0.0541	0.00209	-0.143	0.026	3.336E-08	3256.26433	1E-300	0.02717012	0.955712
ebi-a-GCST006867	ukb-b-13251	Type 2 diabetes	gout	0.29	0.0452	1.4E-10	0.254	0.0516	8.538E-07	2647.95174	1E-300	0.775257357	0.834654
ebi-a-GCST006867	ukb-b-19732	Type 2 diabetes	hypothyroidism/myxoedema	0.148	0.0357	0.0000333	0.1066	0.025	2.227E-05	10924.454	1E-300	0.418563649	0.017839
ebi-a-GCST006867	ukb-b-20289	Type 2 diabetes	hyperthyroidism/thyrotoxicosis	0.0373	0.053	0.481	0.0517	0.033	0.1168	248.427055	5.72E-56	0.8338768	0.514323
ebi-a-GCST006867	ukb-d-I9_HYPTENS	Type 2 diabetes	Hypertension	0.236	0.0895	0.0083	0.215	0.069	0.002	4.53809825	0.033148	0.66990472	0.508983

ebi-a-GCST006867	ukb-e-272_CSA	Type 2 diabetes	Disorders of lipoid metabolism	1.63	0.384	0.0000222	inf	na	na	-0.015866	0.99	0.001132571	0.378436
finngen_R9_DM_HYPOGLYC	finngen_R9_E4_CUSHING	hypoglycemia	Cushing syndrome	1.03	0.459	0.0241	inf	na	na	na	na	0.821496412	0.97464
finngen_R9_DM_HYPOGLYC	ukb-b-12141	hypoglycemia	osteoporosis	-0.0196	0.0834	0.814	-0.132	0.15	0.379	5422.29417	1E-300	0.422520148	0.733127
finngen_R9_DM_HYPOGLYC	ukb-b-13251	hypoglycemia	gout	0.15	0.0803	0.0626	0.15	0.124	0.228	12377.556	1E-300	0.803400994	0.659118
finngen_R9_DM_HYPOGLYC	ukb-b-19732	hypoglycemia	hypothyroidism/myxoedema	0.36	0.0805	0.00000787	0.542	0.215	0.011	70429.7742	1E-300	0.20909256	2.62E-10
finngen_R9_DM_HYPOGLYC	ukb-b-20289	hypoglycemia	hyperthyroidism/thyrotoxicosis	0.243	0.163	0.136	0.285	0.152	0.06	80402.8628	1E-300	0.016678844	0.086169
finngen_R9_DM_HYPOGLYC	ukb-d-I9_HYPTENS	hypoglycemia	Hypertension	0.495	0.143	0.00053	0.662	0.357	0.064	9.43495304	0.002129	0.276131051	0.262754
finngen_R9_DM_HYPOGLYC	ukb-e-272_CSA	hypoglycemia	Disorders of lipoid metabolism	1.07	0.6	0.0733	inf	na	na	-0.0092902	0.99	0.962217936	0.632849
finngen_R9_E4_CUSHING	ukb-b-12141	Cushing syndrome	osteoporosis	-0.778	0.484	0.108	inf	na	na	0.05460528	0.815235	0.458963494	0.969012

finngen_R9_E4_CUSHING	ukb-b-13251	Cushing syndrome	gout	1.03	0.415	0.0132	inf	na	na	0.01537554	0.901317	0.676370977	0.964651
finngen_R9_E4_CUSHING	ukb-b-19732	Cushing syndrome	hypothyroidism/myxoedema	0.381	0.291	0.19	inf	na	na	0.05198713	0.819641	0.326651204	0.797056
finngen_R9_E4_CUSHING	ukb-b-20289	Cushing syndrome	hyperthyroidism/thyrotoxicosis	0.938	0.522	0.0725	inf	na	na	-0.029359	0.99	0.594093121	0.923326
finngen_R9_E4_CUSHING	ukb-d-I9_HYPTENS	Cushing syndrome	Hypertension	0.74	0.9	0.411	inf	na	na	-0.0368071	0.99	0.654558335	0.834818
finngen_R9_E4_CUSHING	ukb-e-272_CSA	Cushing syndrome	Disorders of lipoid metabolism	-7	3.17	0.0274	inf	na	na	-0.0125654	0.99	0.860257095	0.731351
ukb-b-12141	ukb-b-13251	osteoporosis	gout	-0.0335	0.0611	0.584	-0.059	0.039	0.128	2620.78659	1E-300	0.660031789	0.289853
ukb-b-12141	ukb-b-19732	osteoporosis	hypothyroidism/myxoedema	0.0984	0.06	0.101	0.0075	0.024	0.756	11683.1579	1E-300	0.459629781	0.182792
ukb-b-12141	ukb-b-20289	osteoporosis	hyperthyroidism/thyrotoxicosis	0.056	0.0992	0.573	0.129	0.057	0.023	3903.32921	1E-300	0.767365886	0.001704
ukb-b-12141	ukb-d-I9_HYPTENS	osteoporosis	Hypertension	0.113	0.15	0.452	-0.071	0.073	0.333	0.03583211	0.849863	0.920782534	0.173999

ukb-b-12141	ukb-e-272_CSA	osteoporosis	Disorders of lipoid metabolism	0.26	0.561	0.643	inf	na	na	-0.0940891	0.99	0.977702566	0.646132
ukb-b-13251	ukb-b-19732	gout	hypothyroidism/myxoedema	0.158	0.0507	0.00182	0.1197	0.0391	0.0022	15758.66	1E-300	0.754797774	0.803576
ukb-b-13251	ukb-b-20289	gout	hyperthyroidism/thyrotoxicosis	0.168	0.14	0.232	0.0133	0.0418	0.749	21056.676	1E-300	0.875193915	1.79E-05
ukb-b-13251	ukb-d-I9_HYPTENS	gout	Hypertension	0.1	0.134	0.455	0.219	0.066	0.00086	9.67443269	0.001868	0.936464395	0.555753
ukb-b-13251	ukb-e-272_CSA	gout	Disorders of lipoid metabolism	0.14	0.47	0.0264	inf	na	na	0.00799337	0.92876	0.869011916	0.960321
ukb-b-19732	ukb-b-20289	hypothyroidism/myxoedema	hyperthyroidism/thyrotoxicosis	0.443	0.132	0.00076	0.372	0.044	5.532E-17	89257.3802	1E-300	9.21757E-22	0.000976
ukb-b-19732	ukb-d-I9_HYPTENS	hypothyroidism/myxoedema	Hypertension	0.13	0.112	0.243	0.143	0.055	0.008	84.7218383	3.43E-20	0.312648557	0.91794
ukb-b-19732	ukb-e-272_CSA	hypothyroidism/myxoedema	Disorders of lipoid metabolism	-0.108	0.335	0.747	inf	na	na	160.752184	7.75E-37	0.067049969	0.974041

ukb-b-20289	ukb-d-I9_HYPTENS	hyperthyroidism/thyrotoxicosis	Hypertension	0.22	0.143	0.124	0.078	0.113	0.489	211.957588	5.14E-48	0.976079825	0.557269
ukb-b-20289	ukb-e-272_CSA	hyperthyroidism/thyrotoxicosis	Disorders of lipoid metabolism	0.21	0.552	0.703	inf	na	na	11.7761058	0.0006	0.707425583	0.967258
ukb-d-I9_HYPTENS	ukb-e-272_CSA	Hypertension	Disorders of lipoid metabolism	0.55	1.01	0.586	inf	na	na	0.00640278	0.936223	0.860341072	0.991267

Shared Loci Between Common Metabolic Diseases

Trait pair	Trait pair	NO.SNP_PLACO	NO.SNP_FUMA	No. loci_FUMA	loci_FUMA	No. loci_coloc	loci_coloc	exonic	intergenic	intronic	ncRNA_exonic	ncRNA_intronic	ncRNA_splicing
Type 2 diabetes	hypoglycemia	1414	65	32	1p13.2, 2p23.3, 2q36.3, 3p25.2, 3q21.1, 3q27.2, 4p16.1, 4q31.3, 5q11.2, 5q21.1, 6p24.3, 6p22.3, 6p21.31, 6q22.32, 7p15.1, 7q36.3, 8p11.21, 8q24.11, 9p21.3, 10p13, 10q23.33, 10q25.2, 11p15.1, 11q13.4, 11q14.3, 12p13.32, 12p11.22, 12q14.3, 12q24.31, 16q12.2, 18q21.32, 19q13.32	5	1p13.2, 10q23.33, 10q25.2, 12p13.32, 12p11.22	20	958	1275	17	62	0

Type 2 diabetes	osteoporosis	337	19	13	1p34.3, 2p23.3, 3p25.2, 3q27.2, 5q11.2, 6p22.3, 10q25.2, 11q14.3, 12p12.1, 13q31.1, 15q26.1, 16q12.2, 19q13.32	0		3	111	427	7	76	0
Type 2 diabetes	gout	1066	56	24	2p23.3, 2p23.2, 2q24.3, 2q36.3, 3p25.2, 3p21.1, 3q22.3, 3q27.2, 4p16.1, 4q22.1, 5q11.2, 6p25.1, 6p24.3, 6p22.3, 6p22.2, 6p21.1, 6q25.3, 7q11.23, 10q23.33, 10q25.2, 16q12.2, 16q22.1, 18q21.32, 22q13.31	3	2p23.3, 2p23.2, 7q11.23	32	939	1486	19	80	0

Type 2 diabetes	hypothyroidism/myxoedema	3349	143	44	1p34.3, 1p13.3, 1p13.2, 2p21, 2q24.2, 2q32.2, 2q36.3, 3p25.2, 3q27.2, 3q28, 4p16.1, 4p15.2, 5q13.3, 5q21.1, 6p22.3, 6p21.32, 6p21.1, 6q15, 6q23.2, 6q27, 7p21.2, 7q22.1, 8p23.1, 8q24.21, 9p24.2, 9q22.33, 10p15.1, 10q22.3, 10q23.33, 10q25.2, 10q26.13, 11p11.2, 11q12.2, 11q13.4, 11q14.3, 11q21, 12p13.31, 12q23.2, 12q24.11, 12q24.12, 12q24.31, 15q14, 17q21.2, 18q21.32	5	1p13.2, 6p21.1, 7q22.1, 10q26.13, 15q14	59	2489	4262	124	971	2

Type 2 diabetes	Hypertension	182	13	6	2p21, 3q27.2, 4p16.1, 6p22.3, 8q24.11, 10q25.2	0		10	32	671	2	2	0
hypoglycemia	hypothyroidism/myxoedema	1439	26	10	1p13.3, 1p13.2, 2q32.2, 2q33.1, 6p22.2, 6p22.1, 6p21.31, 6q15, 6q27, 11p13	1	1p13.2	17	755	450	19	82	0
gout	hypothyroidism/myxoedema	4680	114	21	1p13.3, 1q21.1, 2q32.2, 4p16.1, 4q22.1, 6p22.2, 6p22.1, 6p21.32, 6q15, 6q27, 9q22.33, 11q13.1, 12q13.2, 12q24.11, 12q24.12, 12q24.13, 12q24.31, 17p13.1, 19p13.3, 19p13.2, 22q12.3	3	12q24.11, 12q24.12, 12q24.13	75	1868	2163	112	274	0

hypothyroidism/myxoedema	hyperthyroidism/thyrotoxicosis	5605	123	37	1p36.13, 1p13.2, 1q23.1, 2p25.3, 2p24.3, 2q24.2, 2q32.2, 2q33.2, 3q13.11, 3q28, 4p16.1, 4p15.2, 4p14, 4q31.23, 5q13.3, 6p25.3, 6p22.2, 6p22.1, 6p21.32, 6q15, 6q27, 8q24.21, 8q24.22, 9p21.3, 10p15.1, 10q21.2, 11q21, 11q24.3, 12q24.11, 12q24.12, 14q31.1, 15q14, 17q21.2, 19p13.3, 21q22.3, 22q12.2, 22q12.3	12	1p13.2, 2q33.2, 3q28, 6q27, 8q24.21, 8q24.22, 10p15.1, 10q21.2, 11q21, 12q24.11, 12q24.12, 19p13.3	67	2471	1853	117	727	0
	Total	18072	559	187		29		283	9623	12587	417	2274	2

Analysis of FUMA

Trait pair	Trait pair	rsID	Chr	BP	Region	LeadSNPs	NearestGene	Func	CADD	RDB	P.PLACO	start	end	PP.H3.abf	PP.H4.abf
Type 2 diabetes	hypoglycemia	rs1230666	1	114173410	1p13.2	rs1230666	MAGI3	intronic	2.8	NA	3.20247E-16	114075796	114451386	0.001	0.996
Type 2 diabetes	hypoglycemia	rs1230661	1	114185590	1p13.2	rs1230661	MAGI3	intronic	10.51	6	3.20247E-16	114075796	114451386	0.001	0.996
Type 2 diabetes	hypoglycemia	rs2476601	1	114377568	1p13.2	rs2476601	PTPN22	exonic	17.25	2b	3.20247E-16	114075796	114451386	0.001	0.996
Type 2 diabetes	hypoglycemia	rs11102694	1	114426001	1p13.2	rs11102694	AP4B1-AS1:BCL2L15	ncRNA_intronic	5.406	6	3.20247E-16	114075796	114451386	0.001	0.996
Type 2 diabetes	hypoglycemia	rs1728918	2	27635463	2p23.3	rs1728918	PPM1G	intergenic	2.458	4	6.97846E-09	27598097	27752871	0.004	0.075
Type 2 diabetes	hypoglycemia	rs1260326	2	27730940	2p23.3	rs1260326	GCKR	exonic	13.22	5	6.97846E-09	27598097	27752871	0.004	0.075
Type 2 diabetes	hypoglycemia	rs7578326	2	227020653	2q36.3	rs7578326	AC068138.1	intergenic	4.856	4	6.97846E-09	27598097	27752871	0.004	0.075
Type 2 diabetes	hypoglycemia	rs1515114	2	227098387	2q36.3	rs1515114	AC068138.1	intergenic	12.88	6	1.00889E-14	227020653	227181683	0.044	0.373
Type 2 diabetes	hypoglycemia	rs2943656	2	227121918	2q36.3	rs2943656	AC068138.1	intergenic	1.095	3a	1.00889E-14	227020653	227181683	0.044	0.373
Type 2 diabetes	hypoglycemia	rs1801282	3	12393125	3p25.2	rs1801282	PPARG	exonic	16.21	7	1.37441E-10	12329783	12413339	0.022	0.211
Type 2 diabetes	hypoglycemia	rs11708067	3	123065778	3q21.1	rs11708067	ADCY5	intronic	19.37	4	1.14271E-09	123051019	123131254	0.005	0.094
Type 2 diabetes	hypoglycemia	rs6767484	3	185520578	3q27.2	rs6767484	IGF2BP2	intronic	3.382	5	3.0518E-17	185488303	185538006	0.021	0.342

Type 2 diabetes	hypoglycemia	rs4234726	4	6272888	4p16.1	rs4234726	WFS1	intronic	6.45	5	3.04397E-12	6263996	6328507	0.021	0.063
Type 2 diabetes	hypoglycemia	rs4689391	4	6280449	4p16.1	rs4689391	WFS1	intronic	0.6	4	3.04397E-12	6263996	6328507	0.021	0.063
Type 2 diabetes	hypoglycemia	rs4688992	4	6316920	4p16.1	rs4688992	PPP2R2C	intergenic	3.079	2b	3.04397E-12	6263996	6328507	0.021	0.063
Type 2 diabetes	hypoglycemia	rs10021011	4	153410390	4q31.3	rs10021011	FBXW7	intronic	0.194	7	1.62392E-08	153234366	153519246	0.060	0.397
Type 2 diabetes	hypoglycemia	rs3936510	5	55860866	5q11.2	rs3936510	AC022431.2	intronic	8.907	NA	1.39801E-08	55854153	55861894	0.003	0.135
Type 2 diabetes	hypoglycemia	rs7729395	5	102100576	5q21.1	rs7729395	PAM	intronic	3.429	5	4.31456E-09	101662386	102586407	0.058	0.304
Type 2 diabetes	hypoglycemia	rs9502583	6	7284487	6p24.3	rs9502583	SSR1:RP11-69L16.4	ncRNA_intronic	0.457	7	3.98002E-08	7275261	7317551	0.042	0.080
Type 2 diabetes	hypoglycemia	rs9295475	6	20652765	6p22.3	rs9295475	CDKAL1	intronic	3.341	4	6.26671E-18	20635026	20741383	0.013	0.335
Type 2 diabetes	hypoglycemia	rs1012635	6	20675295	6p22.3	rs1012635	CDKAL1	intronic	8.511	5	6.26671E-18	20635026	20741383	0.013	0.335
Type 2 diabetes	hypoglycemia	rs7756992	6	20679709	6p22.3	rs7756992	CDKAL1	intronic	0.166	5	6.26671E-18	20635026	20741383	0.013	0.335
Type 2 diabetes	hypoglycemia	rs2206734	6	20694884	6p22.3	rs2206734	CDKAL1	intronic	0.606	5	6.26671E-18	20635026	20741383	0.013	0.335
Type 2 diabetes	hypoglycemia	rs9350276	6	20740296	6p22.3	rs9350276	CDKAL1	intronic	1.979	7	6.26671E-18	20635026	20741383	0.013	0.335
Type 2 diabetes	hypoglycemia	rs563751	6	33552769	6p21.31	rs563751	GGNBP1	intronic	0.519	5	6.7429E-10	33542478	33815924	0.020	0.012
Type 2 diabetes	hypoglycemia	rs4713692	6	33807638	6p21.31	rs4713692	LINC01016	intergenic	2.034	2b	6.7429E-10	33542478	33815924	0.020	0.012
Type 2 diabetes	hypoglycemia	rs1591805	6	126717064	6q22.32	rs1591805	CENPW	intergenic	4.04	NA	1.04409E-09	126659043	127080700	0.438	0.348

Type 2 diabetes	hypoglycemia	rs6918725	6	126990392	6q22.32	rs6918725	RPS4XP9	intergenic	3.776	6	1.04409E-09	126659043	127080700	0.438	0.348
Type 2 diabetes	hypoglycemia	rs881850	7	28152675	7p15.1	rs881850	JAZF1	intronic	1.227	4	6.47618E-14	28142088	28214614	0.007	0.197
Type 2 diabetes	hypoglycemia	rs1635853	7	28189549	7p15.1	rs1635853	JAZF1	intronic	3.204	4	6.47618E-14	28142088	28214614	0.007	0.197
Type 2 diabetes	hypoglycemia	rs849135	7	28196413	7p15.1	rs849135	JAZF1	intronic	1.197	6	6.47618E-14	28142088	28214614	0.007	0.197
Type 2 diabetes	hypoglycemia	rs508347	7	28212824	7p15.1	rs508347	JAZF1	intronic	11.92	5	6.47618E-14	28142088	28214614	0.007	0.197
Type 2 diabetes	hypoglycemia	rs17837729	7	157007665	7q36.3	rs17837729	UBE3C	intronic	3.867	5	8.59278E-09	156926134	157075220	0.093	0.563
Type 2 diabetes	hypoglycemia	rs516946	8	41519248	8p11.21	rs516946	ANK1:RP11-930P14.1	ncRNA_intronic	0.235	NA	7.74899E-10	41488038	41537318	0.004	0.194
Type 2 diabetes	hypoglycemia	rs3802177	8	118185025	8q24.11	rs3802177	SLC30A8	UTR3	3.933	NA	4.81341E-13	118184783	118220270	0.002	0.086
Type 2 diabetes	hypoglycemia	rs7020996	9	22129579	9p21.3	rs7020996	CDKN2B-AS1	intergenic	5.239	7	4.60069E-16	22129579	22136489	0.002	0.270
Type 2 diabetes	hypoglycemia	rs10811661	9	22134094	9p21.3	rs10811661	CDKN2B-AS1	intergenic	4.974	5	4.60069E-16	22129579	22136489	0.002	0.270
Type 2 diabetes	hypoglycemia	rs11257655	10	12307894	10p13	rs11257655	RN7SL232P	intergenic	8.818	4	1.30294E-08	12245520	12328010	0.006	0.146
Type 2 diabetes	hypoglycemia	rs10786048	10	94259968	10q23.33	rs10786048	IDE	intronic	0.112	7	4.69361E-20	94200769	94495241	0.021	0.940
Type 2 diabetes	hypoglycemia	rs11187094	10	94368178	10q23.33	rs11187094	KIF11	intronic	0.026	7	4.69361E-20	94200769	94495241	0.021	0.940
Type 2 diabetes	hypoglycemia	rs10882091	10	94374377	10q23.33	rs10882091	KIF11	intronic	2.786	6	4.69361E-20	94200769	94495241	0.021	0.940
Type 2 diabetes	hypoglycemia	rs1111875	10	94462882	10q23.33	rs1111875	Y_RNA	intergenic	6.157	NA	4.69361E-20	94200769	94495241	0.021	0.940

Type 2 diabetes	hypoglycemia	rs477167	10	114611504	10q25.2	rs477167	RP11-57H14.4	intergenic	1.555	5	1.0096E-112	114490014	114866172	0.006	0.994
Type 2 diabetes	hypoglycemia	rs7901275	10	114732906	10q25.2	rs7901275	TCF7L2	intronic	8.723	7	1.0096E-112	114490014	114866172	0.006	0.994
Type 2 diabetes	hypoglycemia	rs10128255	10	114742835	10q25.2	rs10128255	TCF7L2	intronic	13.15	7	1.0096E-112	114490014	114866172	0.006	0.994
Type 2 diabetes	hypoglycemia	rs4074718	10	114748617	10q25.2	rs4074718	TCF7L2	intronic	7.949	NA	1.0096E-112	114490014	114866172	0.006	0.994
Type 2 diabetes	hypoglycemia	rs7903146	10	114758349	10q25.2	rs7903146	TCF7L2	intronic	3.302	5	1.0096E-112	114490014	114866172	0.006	0.994
Type 2 diabetes	hypoglycemia	rs12266632	10	114764959	10q25.2	rs12266632	TCF7L2	intronic	0.109	5	1.0096E-112	114490014	114866172	0.006	0.994
Type 2 diabetes	hypoglycemia	rs56299331	10	114788436	10q25.2	rs56299331	TCF7L2	intronic	0.698	3a	1.0096E-112	114490014	114866172	0.006	0.994
Type 2 diabetes	hypoglycemia	rs35936842	10	114818772	10q25.2	rs35936842	TCF7L2	intronic	0.598	2b	1.0096E-112	114490014	114866172	0.006	0.994
Type 2 diabetes	hypoglycemia	rs35936842	10	114818772	10q25.2	rs35936842	TCF7L2	NA	7.458	NA	1.0096E-112	114490014	114866172	0.006	0.994
Type 2 diabetes	hypoglycemia	rs10885414	10	114861304	10q25.2	rs10885414	TCF7L2	intronic	0.035	6	1.0096E-112	114490014	114866172	0.006	0.994
Type 2 diabetes	hypoglycemia	rs5215	11	17408630	11p15.1	rs5215	KCNJ11	exonic	14.43	5	2.68869E-10	17368013	17421886	0.025	0.264
Type 2 diabetes	hypoglycemia	rs613937	11	72474839	11q13.4	rs613937	ARAP1:STARD10	intronic	0.008	5	1.51788E-10	72419514	72851463	0.013	0.122
Type 2 diabetes	hypoglycemia	rs10830961	11	92694757	11q14.3	rs10830961	RP11-676F20.1	intergenic	0.025	6	7.35905E-09	92668826	92700287	0.003	0.011
Type 2 diabetes	hypoglycemia	rs12299509	12	4406281	12p13.32	rs12299509	CCND2	intronic	2.778	4	5.73185E-09	4406281	4406281	0.000	0.754

Type 2 diabetes	hypoglycemia	rs10842994	12	27965150	12p11.22	rs10842994	RN7SKP15	intergenic	4.53	5	1.50397E-11	27919006	27967385	0.058	0.797
Type 2 diabetes	hypoglycemia	rs7968682	12	66371880	12q14.3	rs7968682	HMGA2	intergenic	13.76	6	2.16828E-08	66326943	66376202	0.021	0.188
Type 2 diabetes	hypoglycemia	rs1169299	12	121429194	12q24.31	rs1169299	HNF1A	intronic	0.499	7	2.67754E-08	121429194	121451425	0.005	0.272
Type 2 diabetes	hypoglycemia	rs1558902	16	53803574	16q12.2	rs1558902	FTO	intronic	3.29	7	4.68378E-19	53797908	53848561	0.051	0.596
Type 2 diabetes	hypoglycemia	rs2058908	16	53806145	16q12.2	rs2058908	FTO	intronic	6.456	5	4.68378E-19	53797908	53848561	0.051	0.596
Type 2 diabetes	hypoglycemia	rs7190492	16	53828752	16q12.2	rs7190492	FTO	intronic	1.762	6	4.68378E-19	53797908	53848561	0.051	0.596
Type 2 diabetes	hypoglycemia	rs8091524	18	57751960	18q21.32	rs8091524	RP11-795H16.3	intergenic	4.965	6	3.32532E-09	57730096	57913434	0.111	0.603
Type 2 diabetes	hypoglycemia	rs17175643	18	57896742	18q21.32	rs17175643	RP11-795H16.2	intergenic	0.07	7	3.32532E-09	57730096	57913434	0.111	0.603
Type 2 diabetes	hypoglycemia	rs8108269	19	46158513	19q13.32	rs8108269	RN7SL836P	intergenic	6.533	5	3.02293E-08	46148386	46163870	0.005	0.151
Type 2 diabetes	osteoporosis	rs12141058	1	40000354	1p34.3	rs12141058	RP11-69E11.4:PPIEL	ncRNA_intronic	1.987	6	2.87E-09	39942242	40088043	0.206	0.032
Type 2 diabetes	osteoporosis	rs12037222	1	40064961	1p34.3	rs12037222	PABPC4	intergenic	0.223	2b	2.87E-09	39942242	40088043	0.206	0.032
Type 2 diabetes	osteoporosis	rs1260326	2	27730940	2p23.3	rs1260326	GCKR	exonic	13.22	5	5.03E-12	27598097	27752871	0.000	0.064
Type 2 diabetes	osteoporosis	rs11709077	3	12336507	3p25.2	rs11709077	PPARG	intronic	11.87	2b	6.1E-09	12329783	12413339	0.000	0.002
Type 2 diabetes	osteoporosis	rs11927381	3	185508591	3q27.2	rs11927381	IGF2BP2	intronic	2.862	5	2.39E-10	185488303	185548683	0.000	0.001

Type 2 diabetes	osteoporosis	rs11705701	3	185544309	3q27.2	rs11705701	IGF2BP2	intergenic	8.855	3a	2.39E-10	185488303	185548683	0.000	0.001
Type 2 diabetes	osteoporosis	rs13173241	5	55861359	5q11.2	rs13173241	AC022431.2	intronic	4.754	5	4.13E-08	55853349	55861894	0.000	0.003
Type 2 diabetes	osteoporosis	rs10946398	6	20661034	6p22.3	rs10946398	CDKAL1	intronic	2.14	5	4.15E-08	20652717	20703952	0.000	0.000
Type 2 diabetes	osteoporosis	rs10128255	10	114742835	10q25.2	rs10128255	TCF7L2	intronic	13.15	7	1.05E-13	114734743	114866172	0.000	0.000
Type 2 diabetes	osteoporosis	rs11196180	10	114748029	10q25.2	rs11196180	TCF7L2	intronic	2.355	5	1.05E-13	114734743	114866172	0.000	0.000
Type 2 diabetes	osteoporosis	rs11196182	10	114750157	10q25.2	rs11196182	TCF7L2	intronic	1.5	3a	1.05E-13	114734743	114866172	0.000	0.000
Type 2 diabetes	osteoporosis	rs10885402	10	114761697	10q25.2	rs10885402	TCF7L2	intronic	1.491	5	1.05E-13	114734743	114866172	0.000	0.000
Type 2 diabetes	osteoporosis	rs10885414	10	114861304	10q25.2	rs10885414	TCF7L2	intronic	0.035	6	1.05E-13	114734743	114866172	0.000	0.000
Type 2 diabetes	osteoporosis	rs10830963	11	92708710	11q14.3	rs10830963	MTNR1B	intronic	1.762	3a	3.33E-08	92673828	92708710	0.000	0.001
Type 2 diabetes	osteoporosis	rs10842707	12	26471364	12p12.1	rs10842707	RP11-283G6.4:RP11-283G6.5	ncRNA_intronic	9.014	2b	3.77E-08	26440698	26491475	0.001	0.020
Type 2 diabetes	osteoporosis	rs12428731	13	80709595	13q31.1	rs12428731	SPRY2	intergenic	1.399	6	9.51E-10	80677558	80717841	0.015	0.005
Type 2 diabetes	osteoporosis	rs12594752	15	91531995	15q26.1	rs12594752	PRC1	intronic	6.612	4	1.41E-08	91502383	91561182	0.022	0.000
Type 2 diabetes	osteoporosis	rs11075989	16	53819877	16q12.2	rs11075989	FTO	intronic	2.342	6	3.63E-18	53797908	53845487	0.003	0.034
Type 2 diabetes	osteoporosis	rs10422601	19	46159414	19q13.32	rs10422601	RN7SL836P	intergenic	4.092	5	5.46E-10	46148386	46163870	0.001	0.037

Type 2 diabetes	gout	rs11689803	2	27566520	2p23.3	rs11689803	GTF3C2	intronic	6.56	5	1.86086E-40	27404674	28357551	0.008	0.992
Type 2 diabetes	gout	rs1728918	2	27635463	2p23.3	rs1728918	PPM1G	intergenic	2.458	4	1.86086E-40	27404674	28357551	0.008	0.992
Type 2 diabetes	gout	rs4665978	2	27648726	2p23.3	rs4665978	NRBP1	intergenic	1.283	6	1.86086E-40	27404674	28357551	0.008	0.992
Type 2 diabetes	gout	rs780090	2	27718474	2p23.3	rs780090	FNDC4	upstream	13.59	1f	1.86086E-40	27404674	28357551	0.008	0.992
Type 2 diabetes	gout	rs1260326	2	27730940	2p23.3	rs1260326	GCKR	exonic	13.22	5	1.86086E-40	27404674	28357551	0.008	0.992
Type 2 diabetes	gout	rs3817588	2	27731212	2p23.3	rs3817588	GCKR	intronic	0.53	5	1.86086E-40	27404674	28357551	0.008	0.992
Type 2 diabetes	gout	rs12464616	2	27827092	2p23.3	rs12464616	ZNF512:RP11-158I13.2	ncRNA_intronic	2.95	6	1.86086E-40	27404674	28357551	0.008	0.992
Type 2 diabetes	gout	rs2178198	2	27895073	2p23.2	rs2178198	SLC4A1AP	intronic	5.537	6	1.86086E-40	27404674	28357551	0.008	0.992
Type 2 diabetes	gout	rs7607980	2	165551201	2q24.3	rs7607980	COBLL1	exonic	7.978	4	2.91408E-09	165502911	165558215	0.000	0.000
Type 2 diabetes	gout	rs2943656	2	227121918	2q36.3	rs2943656	AC068138.1	intergenic	1.095	3a	1.20662E-11	227020853	227199263	0.000	0.002
Type 2 diabetes	gout	rs10194882	2	227198983	2q36.3	rs10194882	AC068138.1	intergenic	2.324	7	1.20662E-11	227020853	227199263	0.000	0.002
Type 2 diabetes	gout	rs1801282	3	12393125	3p25.2	rs1801282	PPARG	exonic	16.21	7	1.16295E-12	12329783	12413339	0.001	0.029
Type 2 diabetes	gout	rs11242	3	53125922	3p21.1	rs11242	RP11-894J14.5:RFT1	exonic	0.114	NA	4.98327E-09	52970877	53139977	0.981	0.001

Type 2 diabetes	gout	rs9845672	3	138061954	3q22.3	rs9845672	MRAS	intergenic	3.106	7	1.37524E-08	138050685	138123854	0.014	0.172
Type 2 diabetes	gout	rs9859406	3	185534482	3q27.2	rs9859406	IGF2BP2	intronic	0.869	5	6.75275E-10	185488303	185538006	0.000	0.000
Type 2 diabetes	gout	rs4234733	4	6316381	4p16.1	rs4234733	PPP2R2C	intergenic	0.466	1f	4.40813E-08	6295750	6324785	0.000	0.000
Type 2 diabetes	gout	rs10939614	4	9926613	4p16.1	rs10939614	SLC2A9	intronic	3.839	4	2.01302E-16	9915325	10536544	0.189	0.004
Type 2 diabetes	gout	rs4481233	4	9956079	4p16.1	rs4481233	SLC2A9	intronic	5.29	5	2.01302E-16	9915325	10536544	0.189	0.004
Type 2 diabetes	gout	rs10004571	4	10062965	4p16.1	rs10004571	SLC2A9	intergenic	2.908	5	2.01302E-16	9915325	10536544	0.189	0.004
Type 2 diabetes	gout	rs55731306	4	10067885	4p16.1	rs55731306	RP11-448G15.3	intergenic	2.525	7	2.01302E-16	9915325	10536544	0.189	0.004
Type 2 diabetes	gout	rs741077	4	10236142	4p16.1	rs741077	AC006499.9	intergenic	0.773	NA	2.01302E-16	9915325	10536544	0.189	0.004
Type 2 diabetes	gout	rs11728025	4	10408221	4p16.1	rs11728025	RP11-136I13.1	intergenic	0.259	5	2.01302E-16	9915325	10536544	0.189	0.004
Type 2 diabetes	gout	rs2108878	4	10527342	4p16.1	rs2108878	CLNK	intronic	2.374	6	2.01302E-16	9915325	10536544	0.189	0.004
Type 2 diabetes	gout	rs142387850	4	88864048	4q22.1	rs142387850	SPP1	intergenic	2.73	6	1.67126E-10	88843204	89234709	0.130	0.005
Type 2 diabetes	gout	rs2728119	4	88877531	4q22.1	rs2728119	SPP1	intergenic	0.929	NA	1.67126E-10	88843204	89234709	0.130	0.005
Type 2 diabetes	gout	rs4693210	4	89208565	4q22.1	rs4693210	RP11-10L7.1	ncRNA_intronic	0.979	7	1.67126E-10	88843204	89234709	0.130	0.005
Type 2 diabetes	gout	rs1808860	4	89234709	4q22.1	rs1808860	RP11-10L7.1	ncRNA_intronic	0.16	NA	1.67126E-10	88843204	89234709	0.130	0.005

Type 2 diabetes	gout	rs3936510	5	55860866	5q11.2	rs3936510	AC022431.2	intronic	8.907	NA	1.58308E-08	55854153	55861894	0.000	0.002
Type 2 diabetes	gout	rs10458103	6	7037738	6p25.1	rs10458103	snoU13	intergenic	1.765	7	6.29839E-09	7014062	7160014	0.264	0.556
Type 2 diabetes	gout	rs4959424	6	7084857	6p25.1	rs4959424	RREB1	intergenic	1.804	5	6.29839E-09	7014062	7160014	0.264	0.556
Type 2 diabetes	gout	rs7451690	6	7109433	6p24.3	rs7451690	RREB1	intronic	12.14	2b	6.29839E-09	7014062	7160014	0.264	0.556
Type 2 diabetes	gout	rs665723	6	7139064	6p24.3	rs665723	RREB1	intronic	7.937	4	6.29839E-09	7014062	7160014	0.264	0.556
Type 2 diabetes	gout	rs9368222	6	20686996	6p22.3	rs9368222	CDKAL1	intronic	1.737	6	2.72589E-10	20652717	20703952	0.000	0.000
Type 2 diabetes	gout	rs4419666	6	25693274	6p22.2	rs4419666	SCGN	intronic	1.741	6	1.7475E-11	25693274	25879330	0.020	0.105
Type 2 diabetes	gout	rs4145221	6	25763192	6p22.2	rs4145221	SLC17A4	intronic	9.667	7	1.7475E-11	25693274	25879330	0.020	0.105
Type 2 diabetes	gout	rs1165209	6	25801319	6p22.2	rs1165209	SLC17A1	intronic	0.85	7	1.7475E-11	25693274	25879330	0.020	0.105
Type 2 diabetes	gout	rs7753366	6	25817518	6p22.2	rs7753366	SLC17A1	intronic	10.43	7	1.7475E-11	25693274	25879330	0.020	0.105
Type 2 diabetes	gout	rs68137036	6	43820215	6p21.1	rs68137036	RP11-344J7.2	upstream	0.285	4	1.85991E-08	43804103	43829941	0.001	0.016
Type 2 diabetes	gout	rs637614	6	160772133	6q25.3	rs637614	SLC22A3	intronic	2.463	NA	1.17491E-08	160710113	160966347	0.002	0.042
Type 2 diabetes	gout	rs714052	7	72864869	7q11.23	rs714052	BAZ1B	intronic	3.364	NA	1.02081E-13	72841823	73058017	0.102	0.836
Type 2 diabetes	gout	rs7800944	7	73035857	7q11.23	rs7800944	MLXIPL	intronic	0.319	3a	1.02081E-13	72841823	73058017	0.102	0.836
Type 2 diabetes	gout	rs6460047	7	73042443	7q11.23	rs6460047	MLXIPL	intergenic	0.42	5	1.02081E-13	72841823	73058017	0.102	0.836

Type 2 diabetes	gout	rs12778642	10	94464307	10q23.33	rs12778642	Y_RNA	intergenic	1.288	7	1.10184E-11	94248310	94485763	0.000	0.001
Type 2 diabetes	gout	rs7917983	10	114732882	10q25.2	rs7917983	TCF7L2	intronic	5.159	6	1.87709E-09	114722134	114817009	0.000	0.000
Type 2 diabetes	gout	rs10128255	10	114742835	10q25.2	rs10128255	TCF7L2	intronic	13.15	7	1.87709E-09	114722134	114817009	0.000	0.000
Type 2 diabetes	gout	rs7074440	10	114785424	10q25.2	rs7074440	TCF7L2	intronic	9.565	2b	1.87709E-09	114722134	114817009	0.000	0.000
Type 2 diabetes	gout	rs11075987	16	53815161	16q12.2	rs11075987	FTO	intronic	7.828	4	3.84378E-11	53797908	53845487	0.000	0.000
Type 2 diabetes	gout	rs11642841	16	53845487	16q12.2	rs11642841	FTO	intronic	0.26	7	3.84378E-11	53797908	53845487	0.000	0.000
Type 2 diabetes	gout	rs889400	16	69555876	16q22.1	rs889400	NFAT5	intergenic	1.468	7	2.11332E-11	69548788	69940361	0.323	0.667
Type 2 diabetes	gout	rs244415	16	69666683	16q22.1	rs244415	NFAT5	intronic	3.05	6	2.11332E-11	69548788	69940361	0.323	0.667
Type 2 diabetes	gout	rs138993217	16	69770032	16q22.1	rs138993217	CTD-2033A16.3	ncRNA_intronic	2.838	7	2.11332E-11	69548788	69940361	0.323	0.667
Type 2 diabetes	gout	rs7193221	16	69848721	16q22.1	rs7193221	WWP2	intronic	10.4	4	2.11332E-11	69548788	69940361	0.323	0.667
Type 2 diabetes	gout	rs571312	18	57839769	18q21.32	rs571312	RNU4-17P	intergenic	0.484	NA	3.29177E-10	57732418	57912226	0.014	0.042
Type 2 diabetes	gout	rs2294915	22	44340904	22q13.31	rs2294915	PNPLA3	intronic	0.188	5	3.2727E-11	44324727	44395451	0.846	0.145
Type 2 diabetes	gout	rs4823182	22	44377442	22q13.31	rs4823182	SAMM50	intronic	0.339	6	3.2727E-11	44324727	44395451	0.846	0.145
Type 2 diabetes	gout	rs2235776	22	44377999	22q13.31	rs2235776	SAMM50	intronic	9.698	6	3.2727E-11	44324727	44395451	0.846	0.145

Type 2 diabetes	hypothyroidism/myxoedema	rs3818806	1	39748271	1p34.3	rs3818806	MACF1	UTR3	4.314	NA	1.40483E-08	39551488	40035928	0.008	0.012
Type 2 diabetes	hypothyroidism/myxoedema	rs12567695	1	108248583	1p13.3	rs12567695	VAV3	intronic	4.206	7	1.83428E-11	108248583	108409665	0.015	0.036
Type 2 diabetes	hypothyroidism/myxoedema	rs75778624	1	108359790	1p13.3	rs75778624	VAV3	intronic	4.082	5	1.83428E-11	108248583	108409665	0.015	0.036
Type 2 diabetes	hypothyroidism/myxoedema	rs1230666	1	114173410	1p13.2	rs1230666	MAGI3	intronic	2.8	NA	7.17751E-47	114075796	114547798	0.001	0.997
Type 2 diabetes	hypothyroidism/myxoedema	rs1230661	1	114185590	1p13.2	rs1230661	MAGI3	intronic	10.51	6	7.17751E-47	114075796	114547798	0.001	0.997
Type 2 diabetes	hypothyroidism/myxoedema	rs1230673	1	114194513	1p13.2	rs1230673	MAGI3	intronic	2.977	NA	7.17751E-47	114075796	114547798	0.001	0.997
Type 2 diabetes	hypothyroidism/myxoedema	rs1111695	1	114243899	1p13.2	rs1111695	PHTF1	intronic	0.131	NA	7.17751E-47	114075796	114547798	0.001	0.997
Type 2 diabetes	hypothyroidism/myxoedema	rs2476601	1	114377568	1p13.2	rs2476601	PTPN22	exonic	17.25	2b	7.17751E-47	114075796	114547798	0.001	0.997

Type 2 diabetes	hypothyroidism/myxoedema	rs11102694	1	114426001	1p13.2	rs11102694	AP4B1-AS1:BCL2L15	ncRNA_intronic	5.406	6	7.17751E-47	114075796	114547798	0.001	0.997
Type 2 diabetes	hypothyroidism/myxoedema	rs3789613	1	114443035	1p13.2	rs3789613	AP4B1-AS1:AP4B1	ncRNA_exonic	2.94	6	7.17751E-47	114075796	114547798	0.001	0.997
Type 2 diabetes	hypothyroidism/myxoedema	rs17032011	1	114475350	1p13.2	rs17032011	HIPK1	intronic	8.237	1d	7.17751E-47	114075796	114547798	0.001	0.997
Type 2 diabetes	hypothyroidism/myxoedema	rs937672	1	114526216	1p13.2	rs937672	OLFML3	intergenic	7.352	3a	7.17751E-47	114075796	114547798	0.001	0.997
Type 2 diabetes	hypothyroidism/myxoedema	rs61819209	1	114546218	1p13.2	rs61819209	RP4-590F24.1	intergenic	2.926	2b	7.17751E-47	114075796	114547798	0.001	0.997
Type 2 diabetes	hypothyroidism/myxoedema	rs6756560	2	43528478	2p21	rs6756560	THADA	intronic	1.044	4	3.55886E-15	43449385	43850357	0.040	0.021
Type 2 diabetes	hypothyroidism/myxoedema	rs11899984	2	43578246	2p21	rs11899984	THADA	intronic	6.648	2b	3.55886E-15	43449385	43850357	0.040	0.021

Type 2 diabetes	hypothyroidism/myxoedema	rs77881454	2	43757293	2p21	rs77881454	THADA	intronic	0.567	6	3.55886E-15	43449385	43850357	0.040	0.021
Type 2 diabetes	hypothyroidism/myxoedema	rs78098795	2	43840361	2p21	rs78098795	Y_RNA	intergenic	3.549	5	3.55886E-15	43449385	43850357	0.040	0.021
Type 2 diabetes	hypothyroidism/myxoedema	rs2357526	2	160524596	2q24.2	rs2357526	AC009961.3	intergenic	1.799	7	6.65485E-09	160407977	160674656	0.241	0.572
Type 2 diabetes	hypothyroidism/myxoedema	rs7582694	2	191970120	2q32.2	rs7582694	STAT4	intronic	0.069	4	2.85478E-13	191943742	191973034	0.001	0.077
Type 2 diabetes	hypothyroidism/myxoedema	rs13405357	2	227022036	2q36.3	rs13405357	AC068138.1	intergenic	0.665	7	8.92458E-10	227020653	227181683	0.000	0.000
Type 2 diabetes	hypothyroidism/myxoedema	rs307603	3	12152097	3p25.2	rs307603	SYN2	ncRNA_intronic	2.502	NA	2.75493E-15	12026709	12322168	1.000	0.000
Type 2 diabetes	hypothyroidism/myxoedema	rs310746	3	12259616	3p25.2	rs310746	SYN2	intergenic	3.524	NA	2.75493E-15	12026709	12322168	1.000	0.000
Type 2 diabetes	hypothyroidism/myxoedema	rs145268310	3	12310773	3p25.2	rs145268310	GSTM5P1	intergenic	0.186	6	2.75493E-15	12026709	12322168	1.000	0.000

Type 2 diabetes	hypothyroidism/myxoedema	rs13066537	3	12322168	3p25.2	rs13066537	PPARG	intergenic	0.087	5	2.75493E-15	12026709	12322168	1.000	0.000
Type 2 diabetes	hypothyroidism/myxoedema	rs10513800	3	185480388	3q27.2	rs10513800	IGF2BP2	intronic	2.917	7	5.06033E-24	185459675	185548683	0.027	0.465
Type 2 diabetes	hypothyroidism/myxoedema	rs6769511	3	185530290	3q27.2	rs6769511	IGF2BP2	intronic	12.66	5	5.06033E-24	185459675	185548683	0.027	0.465
Type 2 diabetes	hypothyroidism/myxoedema	rs11705701	3	185544309	3q27.2	rs11705701	IGF2BP2	intergenic	8.855	3a	5.06033E-24	185459675	185548683	0.027	0.465
Type 2 diabetes	hypothyroidism/myxoedema	rs13076312	3	188089254	3q28	rs13076312	LPP	intronic	1.292	5	2.95351E-09	188072513	188133518	0.003	0.005
Type 2 diabetes	hypothyroidism/myxoedema	rs4689395	4	6295985	4p16.1	rs4689395	WFS1	intronic	5.862	5	4.59364E-12	6264629	6315029	0.000	0.002
Type 2 diabetes	hypothyroidism/myxoedema	rs1046317	4	6304242	4p16.1	rs1046317	WFS1	UTR3	6.463	1f	4.59364E-12	6264629	6315029	0.000	0.002
Type 2 diabetes	hypothyroidism/myxoedema	rs7441808	4	26090375	4p15.2	rs7441808	RP11-324H7.1	intergenic	14.67	3a	2.60967E-09	26085480	26128710	0.001	0.069

Type 2 diabetes	hypothyroidism/myxoedema	rs1382879	5	76521868	5q13.3	rs1382879	PDE8B	intronic	6.55	NA	2.26409E-09	76515824	76568292	0.023	0.393
Type 2 diabetes	hypothyroidism/myxoedema	rs253759	5	102615675	5q21.1	rs253759	C5orf30	intergenic	1.579	NA	6.34372E-10	102196309	102678073	0.541	0.075
Type 2 diabetes	hypothyroidism/myxoedema	rs9368222	6	20686996	6p22.3	rs9368222	CDKAL1	intronic	1.737	6	2.081E-09	20522718	20904686	0.006	0.001
Type 2 diabetes	hypothyroidism/myxoedema	rs74892619	6	20706071	6p22.3	rs74892619	CDKAL1	intronic	0.68	4	2.081E-09	20522718	20904686	0.006	0.001
Type 2 diabetes	hypothyroidism/myxoedema	rs7767391	6	20725240	6p22.3	rs7767391	CDKAL1	intronic	6.932	6	2.081E-09	20522718	20904686	0.006	0.001
Type 2 diabetes	hypothyroidism/myxoedema	rs6928012	6	20728513	6p22.3	rs6928012	CDKAL1	intronic	6.836	7	2.081E-09	20522718	20904686	0.006	0.001
Type 2 diabetes	hypothyroidism/myxoedema	rs1705003	6	33385953	6p21.32	rs1705003	CUTA	exonic	13.39	2b	2.99447E-11	33317432	33467135	0.005	0.045
Type 2 diabetes	hypothyroidism/myxoedema	rs9369425	6	43810974	6p21.1	rs9369425	RP11-344J7.2	intergenic	0.093	4	1.12256E-11	43804103	43829941	0.031	0.899

Type 2 diabetes	hypothyroidism/myxoedema	rs1010473	6	90856878	6q15	rs1010473	BACH2	intronic	15.96	2b	6.03889E-10	90809560	91012867	0.007	0.004
Type 2 diabetes	hypothyroidism/myxoedema	rs3756784	6	131950233	6q23.2	rs3756784	ENPP3	intronic	0.096	5	1.99803E-09	131897278	131973932	0.012	0.640
Type 2 diabetes	hypothyroidism/myxoedema	rs12203510	6	167473006	6q27	rs12203510	RP11-517H2.6	ncRNA_intronic	1.922	1f	4.41151E-13	167390242	167544278	0.054	0.011
Type 2 diabetes	hypothyroidism/myxoedema	rs1331301	6	167502638	6q27	rs1331301	RP11-517H2.6	ncRNA_intronic	4.746	NA	4.41151E-13	167390242	167544278	0.054	0.011
Type 2 diabetes	hypothyroidism/myxoedema	rs17168486	7	14898282	7p21.2	rs17168486	DGKB	intronic	6.136	3a	3.33303E-08	14859137	14923907	0.000	0.008
Type 2 diabetes	hypothyroidism/myxoedema	rs506597	7	100313420	7q22.1	rs506597	EPO	intergenic	4.185	NA	2.42099E-12	100221867	100366917	0.013	0.984
Type 2 diabetes	hypothyroidism/myxoedema	rs1878561	8	8092405	8p23.1	rs1878561	FAM86B3P	ncRNA_intronic	0.091	NA	6.62216E-18	8088230	12756526	0.971	0.029
Type 2 diabetes	hypothyroidism/myxoedema	rs1107832	8	8146083	8p23.1	rs1107832	SGK223	intergenic	4.345	4	6.62216E-18	8088230	12756526	0.971	0.029

Type 2 diabetes	hypothyroidism/myxoedema	rs60672704	8	8164318	8p23.1	rs60672704	SGK223	intergenic	1.545	5	6.62216E-18	8088230	12756526	0.971	0.029
Type 2 diabetes	hypothyroidism/myxoedema	rs2945238	8	8170136	8p23.1	rs2945238	SGK223	intergenic	2.414	4	6.62216E-18	8088230	12756526	0.971	0.029
Type 2 diabetes	hypothyroidism/myxoedema	rs2979148	8	8262041	8p23.1	rs2979148	CTA-398F10.1	intergenic	1.86	NA	6.62216E-18	8088230	12756526	0.971	0.029
Type 2 diabetes	hypothyroidism/myxoedema	rs2979146	8	8263945	8p23.1	rs2979146	CTA-398F10.1	intergenic	1.184	NA	6.62216E-18	8088230	12756526	0.971	0.029
Type 2 diabetes	hypothyroidism/myxoedema	rs876954	8	8310923	8p23.1	rs876954	CTA-398F10.2	intergenic	3.98	NA	6.62216E-18	8088230	12756526	0.971	0.029
Type 2 diabetes	hypothyroidism/myxoedema	rs2976871	8	8313010	8p23.1	rs2976871	CTA-398F10.2	intergenic	6.952	1f	6.62216E-18	8088230	12756526	0.971	0.029
Type 2 diabetes	hypothyroidism/myxoedema	rs2921094	8	8358667	8p23.1	rs2921094	CTA-398F10.2	intergenic	1.325	5	6.62216E-18	8088230	12756526	0.971	0.029
Type 2 diabetes	hypothyroidism/myxoedema	rs66464561	8	8439879	8p23.1	rs66464561	RN7SL178P	ncRNA_exonic	3.112	7	6.62216E-18	8088230	12756526	0.971	0.029

Type 2 diabetes	hypothyroidism/myxoedema	rs28472946	8	8443173	8p23.1	rs28472946	RN7SL178P	intergenic	0.02	7	6.62216E-18	8088230	12756526	0.971	0.029
Type 2 diabetes	hypothyroidism/myxoedema	rs4841009	8	8514186	8p23.1	rs4841009	AC087269.2	intergenic	0.037	4	6.62216E-18	8088230	12756526	0.971	0.029
Type 2 diabetes	hypothyroidism/myxoedema	rs111714591	8	8548877	8p23.1	rs111714591	CLDN23	intergenic	0.154	6	6.62216E-18	8088230	12756526	0.971	0.029
Type 2 diabetes	hypothyroidism/myxoedema	rs28729565	8	8551896	8p23.1	rs28729565	CLDN23	intergenic	0.781	6	6.62216E-18	8088230	12756526	0.971	0.029
Type 2 diabetes	hypothyroidism/myxoedema	rs546603	8	8595838	8p23.1	rs546603	RP11-211C9.1	ncRNA_intronic	3.232	5	6.62216E-18	8088230	12756526	0.971	0.029
Type 2 diabetes	hypothyroidism/myxoedema	rs9329167	8	8664097	8p23.1	rs9329167	MFHAS1	intronic	1.509	2b	6.62216E-18	8088230	12756526	0.971	0.029
Type 2 diabetes	hypothyroidism/myxoedema	rs2409096	8	8692477	8p23.1	rs2409096	MFHAS1	intronic	4.006	6	6.62216E-18	8088230	12756526	0.971	0.029
Type 2 diabetes	hypothyroidism/myxoedema	rs3789843	8	8724257	8p23.1	rs3789843	MFHAS1	intronic	0.004	NA	6.62216E-18	8088230	12756526	0.971	0.029

Type 2 diabetes	hypothyroidism/myxoedema	rs17700611	8	8793654	8p23.1	rs17700611	RP11-62H7.3	intergenic	1.214	1f	6.62216E-18	8088230	12756526	0.971	0.029
Type 2 diabetes	hypothyroidism/myxoedema	rs2921378	8	8914812	8p23.1	rs2921378	ERI1	intergenic	1.465	5	6.62216E-18	8088230	12756526	0.971	0.029
Type 2 diabetes	hypothyroidism/myxoedema	rs11775429	8	8925462	8p23.1	rs11775429	ERI1	intergenic	0.07	5	6.62216E-18	8088230	12756526	0.971	0.029
Type 2 diabetes	hypothyroidism/myxoedema	rs10111263	8	8969433	8p23.1	rs10111263	ERI1	intergenic	0.411	6	6.62216E-18	8088230	12756526	0.971	0.029
Type 2 diabetes	hypothyroidism/myxoedema	rs435581	8	9029413	8p23.1	rs435581	RP11-10A14.4	intergenic	3.439	NA	6.62216E-18	8088230	12756526	0.971	0.029
Type 2 diabetes	hypothyroidism/myxoedema	rs2010390	8	9047178	8p23.1	rs2010390	RP11-10A14.5	ncRNA_intronic	2.056	NA	6.62216E-18	8088230	12756526	0.971	0.029
Type 2 diabetes	hypothyroidism/myxoedema	rs330048	8	9087278	8p23.1	rs330048	RP11-10A14.8	intergenic	0.794	1f	6.62216E-18	8088230	12756526	0.971	0.029
Type 2 diabetes	hypothyroidism/myxoedema	rs7843880	8	9099173	8p23.1	rs7843880	RP11-115J16.1	intergenic	0.649	6	6.62216E-18	8088230	12756526	0.971	0.029

Type 2 diabetes	hypothyroidism/myxoedema	rs7387873	8	9145927	8p23.1	rs7387873	RP11-115J16.1	ncRNA_intronic	3.929	6	6.62216E-18	8088230	12756526	0.971	0.029
Type 2 diabetes	hypothyroidism/myxoedema	rs11781008	8	9153239	8p23.1	rs11781008	RP11-115J16.1	ncRNA_intronic	4.306	5	6.62216E-18	8088230	12756526	0.971	0.029
Type 2 diabetes	hypothyroidism/myxoedema	rs11774915	8	9188762	8p23.1	rs11774915	RP11-115J16.1	ncRNA_intronic	2.5	7	6.62216E-18	8088230	12756526	0.971	0.029
Type 2 diabetes	hypothyroidism/myxoedema	rs35847492	8	9276025	8p23.1	rs35847492	RP11-115J16.2	ncRNA_intronic	1.3	4	6.62216E-18	8088230	12756526	0.971	0.029
Type 2 diabetes	hypothyroidism/myxoedema	rs6980728	8	9390529	8p23.1	rs6980728	RP11-375N15.2	intergenic	0.517	7	6.62216E-18	8088230	12756526	0.971	0.029
Type 2 diabetes	hypothyroidism/myxoedema	rs10107396	8	9673152	8p23.1	rs10107396	TNKS	intergenic	0.06	7	6.62216E-18	8088230	12756526	0.971	0.029
Type 2 diabetes	hypothyroidism/myxoedema	rs17734541	8	9708433	8p23.1	rs17734541	snoU13	intergenic	0.889	6	6.62216E-18	8088230	12756526	0.971	0.029
Type 2 diabetes	hypothyroidism/myxoedema	rs615632	8	9796321	8p23.1	rs615632	snoU13	intergenic	1.195	5	6.62216E-18	8088230	12756526	0.971	0.029

Type 2 diabetes	hypothyroidism/myxoedema	rs10111315	8	9865588	8p23.1	rs10111315	RP11-1E4.1	intergenic	8.47	7	6.62216E-18	8088230	12756526	0.971	0.029
Type 2 diabetes	hypothyroidism/myxoedema	rs2062331	8	9979992	8p23.1	rs2062331	MSRA	intronic	3.032	5	6.62216E-18	8088230	12756526	0.971	0.029
Type 2 diabetes	hypothyroidism/myxoedema	rs6601450	8	10243101	8p23.1	rs6601450	MSRA	intronic	0.343	5	6.62216E-18	8088230	12756526	0.971	0.029
Type 2 diabetes	hypothyroidism/myxoedema	rs1962073	8	10260553	8p23.1	rs1962073	MSRA	intronic	0.822	5	6.62216E-18	8088230	12756526	0.971	0.029
Type 2 diabetes	hypothyroidism/myxoedema	rs7833781	8	10333477	8p23.1	rs7833781	RP11-981G7.2	ncRNA_intronic	2.793	6	6.62216E-18	8088230	12756526	0.971	0.029
Type 2 diabetes	hypothyroidism/myxoedema	rs7002088	8	10494738	8p23.1	rs7002088	RP1L1	intronic	5.818	5	6.62216E-18	8088230	12756526	0.971	0.029
Type 2 diabetes	hypothyroidism/myxoedema	rs4841407	8	10516185	8p23.1	rs4841407	RP1L1	intergenic	0.233	5	6.62216E-18	8088230	12756526	0.971	0.029

Type 2 diabetes	hypothyroidism/myxoedema	rs9650657	8	10607400	8p23.1	rs9650657	SOX7:SOX7:CTD-2135J3.3	ncRNA_intronic	2.714	6	6.62216E-18	8088230	12756526	0.971	0.029
Type 2 diabetes	hypothyroidism/myxoedema	rs6997997	8	10766082	8p23.1	rs6997997	XKR6	intronic	1.219	7	6.62216E-18	8088230	12756526	0.971	0.029
Type 2 diabetes	hypothyroidism/myxoedema	rs7821914	8	10805015	8p23.1	rs7821914	XKR6	intronic	3.386	1f	6.62216E-18	8088230	12756526	0.971	0.029
Type 2 diabetes	hypothyroidism/myxoedema	rs13248300	8	10926675	8p23.1	rs13248300	XKR6	intronic	0.295	6	6.62216E-18	8088230	12756526	0.971	0.029
Type 2 diabetes	hypothyroidism/myxoedema	rs4841501	8	11000976	8p23.1	rs4841501	XKR6	intronic	4.018	6	6.62216E-18	8088230	12756526	0.971	0.029
Type 2 diabetes	hypothyroidism/myxoedema	rs10156356	8	11046209	8p23.1	rs10156356	XKR6	intronic	6.142	5	6.62216E-18	8088230	12756526	0.971	0.029
Type 2 diabetes	hypothyroidism/myxoedema	rs2409745	8	11076635	8p23.1	rs2409745	AF131215.8	intergenic	1.543	6	6.62216E-18	8088230	12756526	0.971	0.029

Type 2 diabetes	hypothyroidism/myxoedema	rs10098488	8	11130977	8p23.1	rs10098488	LINC00529	intergenic	0.423	6	6.62216E-18	8088230	12756526	0.971	0.029
Type 2 diabetes	hypothyroidism/myxoedema	rs2736389	8	11161310	8p23.1	rs2736389	MTMR9	intronic	0.19	7	6.62216E-18	8088230	12756526	0.971	0.029
Type 2 diabetes	hypothyroidism/myxoedema	rs7834139	8	11206363	8p23.1	rs7834139	TDH	ncRNA_intronic	2.289	2b	6.62216E-18	8088230	12756526	0.971	0.029
Type 2 diabetes	hypothyroidism/myxoedema	rs11787413	8	11240571	8p23.1	rs11787413	C8orf12	intronic	1.086	7	6.62216E-18	8088230	12756526	0.971	0.029
Type 2 diabetes	hypothyroidism/myxoedema	rs2572440	8	11249010	8p23.1	rs2572440	C8orf12	intronic	1.583	NA	6.62216E-18	8088230	12756526	0.971	0.029
Type 2 diabetes	hypothyroidism/myxoedema	rs10104336	8	11268344	8p23.1	rs10104336	C8orf12	intronic	0.111	6	6.62216E-18	8088230	12756526	0.971	0.029
Type 2 diabetes	hypothyroidism/myxoedema	rs7836059	8	11272164	8p23.1	rs7836059	C8orf12	intronic	2.672	4	6.62216E-18	8088230	12756526	0.971	0.029
Type 2 diabetes	hypothyroidism/myxoedema	rs13439487	8	11310529	8p23.1	rs13439487	FAM167A	intronic	4.203	5	6.62216E-18	8088230	12756526	0.971	0.029

Type 2 diabetes	hypothyroidism/myxoedema	rs1564267	8	11337887	8p23.1	rs1564267	FAM167A	intergenic	4.079	1d	6.62216E-18	8088230	12756526	0.971	0.029
Type 2 diabetes	hypothyroidism/myxoedema	rs13272061	8	11352261	8p23.1	rs13272061	BLK	intronic	3.657	2b	6.62216E-18	8088230	12756526	0.971	0.029
Type 2 diabetes	hypothyroidism/myxoedema	rs1478887	8	11355980	8p23.1	rs1478887	BLK	intronic	0.618	NA	6.62216E-18	8088230	12756526	0.971	0.029
Type 2 diabetes	hypothyroidism/myxoedema	rs2618443	8	11384556	8p23.1	rs2618443	BLK	intronic	2.083	NA	6.62216E-18	8088230	12756526	0.971	0.029
Type 2 diabetes	hypothyroidism/myxoedema	rs10098664	8	11417493	8p23.1	rs10098664	BLK:RP11-148021.2	ncRNA_exonic	4.147	7	6.62216E-18	8088230	12756526	0.971	0.029
Type 2 diabetes	hypothyroidism/myxoedema	rs2244894	8	11448659	8p23.1	rs2244894	LINC00208	intergenic	4.266	5	6.62216E-18	8088230	12756526	0.971	0.029
Type 2 diabetes	hypothyroidism/myxoedema	rs35373084	8	11457248	8p23.1	rs35373084	LINC00208	intergenic	0.726	NA	6.62216E-18	8088230	12756526	0.971	0.029
Type 2 diabetes	hypothyroidism/myxoedema	rs13273172	8	11461111	8p23.1	rs13273172	LINC00208	intergenic	0.311	6	6.62216E-18	8088230	12756526	0.971	0.029

Type 2 diabetes	hypothyroidism/myxoedema	rs17153559	8	11491128	8p23.1	rs17153559	GATA4	intergenic	0.259	5	6.62216E-18	8088230	12756526	0.971	0.029
Type 2 diabetes	hypothyroidism/myxoedema	rs34421088	8	11589042	8p23.1	rs34421088	GATA4	intronic	6.452	7	6.62216E-18	8088230	12756526	0.971	0.029
Type 2 diabetes	hypothyroidism/myxoedema	rs7819276	8	11755804	8p23.1	rs7819276	RP11-589N15.1	intergenic	5.545	2b	6.62216E-18	8088230	12756526	0.971	0.029
Type 2 diabetes	hypothyroidism/myxoedema	rs12681142	8	11802601	8p23.1	rs12681142	OR7E161P	intergenic	0.712	6	6.62216E-18	8088230	12756526	0.971	0.029
Type 2 diabetes	hypothyroidism/myxoedema	rs12681142	8	11802601	8p23.1	rs12681142	OR7E161P	NA	0.607	NA	6.62216E-18	8088230	12756526	0.971	0.029
Type 2 diabetes	hypothyroidism/myxoedema	rs7460395	8	11835375	8p23.1	rs7460395	DEFB136	intergenic	2.796	1f	6.62216E-18	8088230	12756526	0.971	0.029
Type 2 diabetes	hypothyroidism/myxoedema	rs10086500	8	11838932	8p23.1	rs10086500	DEFB135	upstream	1.6	6	6.62216E-18	8088230	12756526	0.971	0.029
Type 2 diabetes	hypothyroidism/myxoedema	rs4841662	8	11843758	8p23.1	rs4841662	DEFB135	intergenic	5.376	1d	6.62216E-18	8088230	12756526	0.971	0.029

Type 2 diabetes	hypothyroidism/myxoedema	rs1561927	8	129568078	8q24.21	rs1561927	RP11-89M16.1	ncRNA_intronic	0.599	4	6.51432E-10	129534534	129592699	0.558	0.213
Type 2 diabetes	hypothyroidism/myxoedema	rs28455755	8	129571140	8q24.21	rs28455755	RP11-89M16.1	ncRNA_intronic	1.763	7	6.51432E-10	129534534	129592699	0.558	0.213
Type 2 diabetes	hypothyroidism/myxoedema	rs7825794	8	129577486	8q24.21	rs7825794	RP11-89M16.1	upstream	3.74	5	6.51432E-10	129534534	129592699	0.558	0.213
Type 2 diabetes	hypothyroidism/myxoedema	rs10758593	9	4292083	9p24.2	rs10758593	GLIS3	intronic	7.301	5	6.48344E-11	4282536	4296430	0.002	0.013
Type 2 diabetes	hypothyroidism/myxoedema	rs10759944	9	100556972	9q22.33	rs10759944	RP11-23B15.1	intergenic	0.115	2b	8.24308E-22	100533317	100670272	0.003	0.019
Type 2 diabetes	hypothyroidism/myxoedema	rs7859751	9	100575888	9q22.33	rs7859751	RP11-23B15.1	intergenic	0.6	7	8.24308E-22	100533317	100670272	0.003	0.019
Type 2 diabetes	hypothyroidism/myxoedema	rs13302470	9	100615553	9q22.33	rs13302470	FOXE1	UTR5	9.435	4	8.24308E-22	100533317	100670272	0.003	0.019
Type 2 diabetes	hypothyroidism/myxoedema	rs7072793	10	6106266	10p15.1	rs7072793	IL2RA	intergenic	4.523	5	7.06205E-09	6098824	6110875	0.001	0.048

Type 2 diabetes	hypothyroidism/myxoedema	rs7090530	10	6110875	10p15.1	rs7090530	RP11-414H17.2	intergenic	1.856	5	7.06205E-09	6098824	6110875	0.001	0.048
Type 2 diabetes	hypothyroidism/myxoedema	rs1250591	10	80985374	10q22.3	rs1250591	ZMIZ1	intronic	2.432	7	7.69351E-10	80959973	80989082	0.001	0.185
Type 2 diabetes	hypothyroidism/myxoedema	rs1111875	10	94462882	10q23.33	rs1111875	Y_RNA	intergenic	6.157	NA	6.26513E-11	94208397	94485763	0.000	0.002
Type 2 diabetes	hypothyroidism/myxoedema	rs7901275	10	114732906	10q25.2	rs7901275	TCF7L2	intronic	8.723	7	2.26217E-16	114722134	114818772	0.000	0.000
Type 2 diabetes	hypothyroidism/myxoedema	rs10128255	10	114742835	10q25.2	rs10128255	TCF7L2	intronic	13.15	7	2.26217E-16	114722134	114818772	0.000	0.000
Type 2 diabetes	hypothyroidism/myxoedema	rs17747324	10	114752503	10q25.2	rs17747324	TCF7L2	intronic	1.161	6	2.26217E-16	114722134	114818772	0.000	0.000
Type 2 diabetes	hypothyroidism/myxoedema	rs10787472	10	114781297	10q25.2	rs10787472	TCF7L2	intronic	1.589	5	2.26217E-16	114722134	114818772	0.000	0.000
Type 2 diabetes	hypothyroidism/myxoedema	rs61872784	10	114798893	10q25.2	rs61872784	TCF7L2	intronic	4.281	5	2.26217E-16	114722134	114818772	0.000	0.000

Type 2 diabetes	hypothyroidism/myxoedema	rs10510109	10	124120457	10q26.13	rs10510109	PLEKHA1	intergenic	0.557	7	1.06104E-15	124104221	124198585	0.290	0.710
Type 2 diabetes	hypothyroidism/myxoedema	rs7068487	10	124145212	10q26.13	rs7068487	PLEKHA1	intronic	4.089	7	1.06104E-15	124104221	124198585	0.290	0.710
Type 2 diabetes	hypothyroidism/myxoedema	rs2421016	10	124167512	10q26.13	rs2421016	PLEKHA1	intronic	17.59	NA	1.06104E-15	124104221	124198585	0.290	0.710
Type 2 diabetes	hypothyroidism/myxoedema	rs7124681	11	47529947	11p11.2	rs7124681	CELF1	intronic	3.081	1f	2.00552E-08	47372377	47946836	0.791	0.019
Type 2 diabetes	hypothyroidism/myxoedema	rs174594	11	61619829	11q12.2	rs174594	FADS2	intronic	2.049	NA	1.50352E-10	61542006	61624181	0.029	0.696
Type 2 diabetes	hypothyroidism/myxoedema	rs11603349	11	72460694	11q13.4	rs11603349	ARAP1	intronic	0.999	5	8.67098E-13	72419514	72851463	0.016	0.051
Type 2 diabetes	hypothyroidism/myxoedema	rs10830956	11	92681013	11q14.3	rs10830956	SNRPGP16	intergenic	2.191	6	1.73931E-11	92668826	92708710	0.000	0.003
Type 2 diabetes	hypothyroidism/myxoedema	rs2166706	11	92691532	11q14.3	rs2166706	RP11-676F20.1	intergenic	3.042	7	1.73931E-11	92668826	92708710	0.000	0.003

Type 2 diabetes	hypothyroidism/myxoedema	rs11021232	11	95320808	11q21	rs11021232	RP11-338H14.1	intergenic	1.81	5	1.19334E-09	95298828	95320808	0.000	0.019
Type 2 diabetes	hypothyroidism/myxoedema	rs10844503	12	9854256	12p13.31	rs10844503	RP11-75L1.1	intergenic	1.376	4	7.26478E-12	9804373	9910132	0.010	0.035
Type 2 diabetes	hypothyroidism/myxoedema	rs7977720	12	9866349	12p13.31	rs7977720	CLECL1	intergenic	1.315	6	7.26478E-12	9804373	9910132	0.010	0.035
Type 2 diabetes	hypothyroidism/myxoedema	rs2374053	12	103747178	12q23.2	rs2374053	C12orf42	intronic	14.48	7	4.21499E-09	103652558	103769787	0.019	0.380
Type 2 diabetes	hypothyroidism/myxoedema	rs2028005	12	111361137	12q24.11	rs2028005	MYL2	intergenic	1.043	NA	2.3955E-27	111359712	112985328	0.013	0.435
Type 2 diabetes	hypothyroidism/myxoedema	rs3847953	12	111765464	12q24.12	rs3847953	CUX2	intronic	2.838	NA	2.3955E-27	111359712	112985328	0.013	0.435
Type 2 diabetes	hypothyroidism/myxoedema	rs3184504	12	111884608	12q24.12	rs3184504	SH2B3	exonic	11.21	3a	2.3955E-27	111359712	112985328	0.013	0.435
Type 2 diabetes	hypothyroidism/myxoedema	rs593226	12	111993886	12q24.12	rs593226	ATXN2	intronic	5.648	7	2.3955E-27	111359712	112985328	0.013	0.435

Type 2 diabetes	hypothyroidism/myxoedema	rs847892	12	112141570	12q24.12	rs847892	ACAD10	intronic	0.934	NA	2.3955E-27	111359712	112985328	0.013	0.435
Type 2 diabetes	hypothyroidism/myxoedema	rs4766897	12	112179471	12q24.12	rs4766897	ACAD10	intronic	1.631	2b	2.3955E-27	111359712	112985328	0.013	0.435
Type 2 diabetes	hypothyroidism/myxoedema	rs11066320	12	112906415	12q24.12	rs11066320	PTPN11	intronic	3.412	6	2.3955E-27	111359712	112985328	0.013	0.435
Type 2 diabetes	hypothyroidism/myxoedema	rs692902	12	121197124	12q24.31	rs692902	RP11-173P15.7	ncRNA_intronic	4.172	4	1.24773E-09	121189116	121489657	0.133	0.008
Type 2 diabetes	hypothyroidism/myxoedema	rs3213566	12	121222578	12q24.31	rs3213566	SPPL3	intronic	0.493	NA	1.24773E-09	121189116	121489657	0.133	0.008
Type 2 diabetes	hypothyroidism/myxoedema	rs7305618	12	121402932	12q24.31	rs7305618	HNF1A-AS1	intergenic	1.777	4	1.24773E-09	121189116	121489657	0.133	0.008
Type 2 diabetes	hypothyroidism/myxoedema	rs2251468	12	121405126	12q24.31	rs2251468	HNF1A-AS1	intergenic	0.791	1f	1.24773E-09	121189116	121489657	0.133	0.008
Type 2 diabetes	hypothyroidism/myxoedema	rs7965349	12	121471931	12q24.31	rs7965349	OASL	intronic	0.073	4	1.24773E-09	121189116	121489657	0.133	0.008

Type 2 diabetes	hypothyroidism/myxoedema	rs12593201	15	38844106	15q14	rs12593201	RASGRP1	intronic	7.223	7	1.24773E-09	121189116	121489657	NA	NA
Type 2 diabetes	hypothyroidism/myxoedema	rs16967104	15	38900699	15q14	rs16967104	RASGRP1	intergenic	1.584	7	1.24773E-09	121189116	121489657	NA	NA
Type 2 diabetes	hypothyroidism/myxoedema	rs12600570	17	40261545	17q21.2	rs12600570	DHX58	intronic	0.136	1f	8.98292E-12	40256498	40546917	0.992	0.008
Type 2 diabetes	hypothyroidism/myxoedema	rs11079035	17	40289012	17q21.2	rs11079035	CTD-2132N18.3:RAB5C	intronic	2.557	6	8.98292E-12	40256498	40546917	0.992	0.008
Type 2 diabetes	hypothyroidism/myxoedema	rs116920984	17	40491135	17q21.2	rs116920984	STAT3	intronic	0.003	6	8.98292E-12	40256498	40546917	0.992	0.008
Type 2 diabetes	hypothyroidism/myxoedema	rs12970134	18	57884750	18q21.32	rs12970134	RP11-795H16.2	intergenic	0.478	5	6.16817E-10	57732418	57913434	0.015	0.149
Type 2 diabetes	Hypertension	rs13411485	2	43575984	2p21	rs13411485	THADA	intronic	0.675	5	4.27877E-08	43449385	43778470	0.000	0.000
Type 2 diabetes	Hypertension	rs10513800	3	185480388	3q27.2	rs10513800	IGF2BP2	intronic	2.917	7	3.88452E-09	185459675	185538006	0.000	0.000
Type 2 diabetes	Hypertension	rs4686696	3	185516520	3q27.2	rs4686696	IGF2BP2	intronic	2.191	4	3.88452E-09	185459675	185538006	0.000	0.000
Type 2 diabetes	Hypertension	rs6820509	4	6295750	4p16.1	rs6820509	WFS1	intronic	2.707	5	5.798E-10	6263996	6321396	0.000	0.000

Type 2 diabetes	Hypertension	rs1046319	4	6304286	4p16.1	rs1046319	WFS1	UTR3	2.787	1f	5.798E-10	6263996	6321396	0.000	0.000
Type 2 diabetes	Hypertension	rs2206734	6	20694884	6p22.3	rs2206734	CDKAL1	intronic	0.606	5	3.49579E-13	20641336	20727570	0.000	0.000
Type 2 diabetes	Hypertension	rs6931514	6	20703952	6p22.3	rs6931514	CDKAL1	intronic	3.617	7	3.49579E-13	20641336	20727570	0.000	0.000
Type 2 diabetes	Hypertension	rs11774700	8	118220270	8q24.11	rs11774700	SLC30A8	intergenic	0.218	7	6.76801E-09	118184783	118220270	0.000	0.000
Type 2 diabetes	Hypertension	rs7901275	10	114732906	10q25.2	rs7901275	TCF7L2	intronic	8.723	7	6.18815E-15	114722134	114818772	0.000	0.000
Type 2 diabetes	Hypertension	rs4073288	10	114747277	10q25.2	rs4073288	TCF7L2:RP11-139K1.2	ncRNA_exonic	5.741	NA	6.18815E-15	114722134	114818772	0.000	0.000
Type 2 diabetes	Hypertension	rs10787472	10	114781297	10q25.2	rs10787472	TCF7L2	intronic	1.589	5	6.18815E-15	114722134	114818772	0.000	0.000
Type 2 diabetes	Hypertension	rs7074440	10	114785424	10q25.2	rs7074440	TCF7L2	intronic	9.565	2b	6.18815E-15	114722134	114818772	0.000	0.000
Type 2 diabetes	Hypertension	rs56299331	10	114788436	10q25.2	rs56299331	TCF7L2	intronic	0.698	3a	6.18815E-15	114722134	114818772	0.000	0.000
hypoglycemia	hypothyroidism/myxoedema	rs4915073	1	107783257	1p13.3	rs4915073	NTNG1	intronic	6.446	NA	3.75119E-14	106788968	108407862	0.189	0.101
hypoglycemia	hypothyroidism/myxoedema	rs9787296	1	107840205	1p13.3	rs9787296	NTNG1	intronic	2.975	4	3.75119E-14	106788968	108407862	0.189	0.101

hypoglycemia	hypothyroidism/myxoedema	rs1217236	1	113590090	1p13.2	rs1217236	RP11-31F15.2	ncRNA_intronic	4.702	NA	1.50589E-30	112885855	114432426	0.000	1.000
hypoglycemia	hypothyroidism/myxoedema	rs1230678	1	113755798	1p13.2	rs1230678	RP11-389022.5	intergenic	0.36	7	1.50589E-30	112885855	114432426	0.000	1.000
hypoglycemia	hypothyroidism/myxoedema	rs1020658	2	191131412	2q32.2	rs1020658	HIBCH	intronic	0.214	NA	8.68414E-09	190373010	191985304	0.070	0.082
hypoglycemia	hypothyroidism/myxoedema	rs1427679	2	203859027	2q33.1	rs1427679	WDR12	upstream	6.64	7	2.86588E-22	202930214	204613717	0.093	0.418
hypoglycemia	hypothyroidism/myxoedema	rs2352546	2	203894023	2q33.1	rs2352546	NBEAL1	intronic	2.668	NA	2.86588E-22	202930214	204613717	0.093	0.418
hypoglycemia	hypothyroidism/myxoedema	rs3922681	6	25698253	6p22.2	rs3922681	SCGN	intronic	3.239	4	1.18476E-09	25685530	25873184	0.427	0.573
hypoglycemia	hypothyroidism/myxoedema	rs3922681	6	25698481	6p22.2	rs9379786	SCGN	intronic	4.991	NA	1.18476E-09	25685530	25873184	0.427	0.573
hypoglycemia	hypothyroidism/myxoedema	rs9379786	6	25730027	6p22.2	rs9379786	HIST1H2BPS1	intergenic	0.367	6	1.18476E-09	25685530	25873184	0.427	0.573

hypoglycemia	hypothyroidism/myxoedema	rs9379786	6	25730255	6p22.2	rs9379786	HIST1H2BPS1	intergenic	0.754	6	1.18476E-09	25685530	25873184	0.427	0.573
hypoglycemia	hypothyroidism/myxoedema	rs1977200	6	26466164	6p22.2	rs1977200	BTN2A1	intronic	5.89	NA	1.2947E-18	26340450	29602717	0.864	0.136
hypoglycemia	hypothyroidism/myxoedema	rs66785117	6	27555519	6p22.1	rs66785117	RNU6-471P	intergenic	2.782	6	1.2947E-18	26340450	29602717	0.864	0.136
hypoglycemia	hypothyroidism/myxoedema	rs200501	6	27821164	6p22.1	rs200501	HIST1H2BN	UTR3	3.024	NA	1.2947E-18	26340450	29602717	0.864	0.136
hypoglycemia	hypothyroidism/myxoedema	rs35072899	6	28313764	6p22.1	rs35072899	ZSCAN31	intronic	3.923	7	1.2947E-18	26340450	29602717	0.864	0.136
hypoglycemia	hypothyroidism/myxoedema	rs7767099	6	28800921	6p22.1	rs7767099	XXbac-BPG308K3.5	intergenic	5.9	NA	1.2947E-18	26340450	29602717	0.864	0.136
hypoglycemia	hypothyroidism/myxoedema	rs3130729	6	29157970	6p22.1	rs3130729	OR2J4P	intergenic	0.199	6	1.2947E-18	26340450	29602717	0.864	0.136
hypoglycemia	hypothyroidism/myxoedema	rs3117337	6	29243779	6p22.1	rs3117337	XXbac-BPG308J9.3	ncRNA_intronic	7.914	NA	1.2947E-18	26340450	29602717	0.864	0.136

hypoglycemia	hypothyroidism/myxoedema	rs3130856	6	29544551	6p22.1	rs3130856	GABBR1	intronic	2.036	7	1.2947E-18	26340450	29602717	0.864	0.136
hypoglycemia	hypothyroidism/myxoedema	rs1233397	6	29577938	6p22.1	rs1233397	GABBR1	intronic	4.552	NA	1.2947E-18	26340450	29602717	0.864	0.136
hypoglycemia	hypothyroidism/myxoedema	rs34213779	6	33626143	6p21.31	rs34213779	ITPR3	intronic	7.499	NA	2.96242E-08	33176725	34529900	1.000	0.000
hypoglycemia	hypothyroidism/myxoedema	rs71565398	6	33633953	6p21.31	rs71565398	ITPR3	intronic	0.667	NA	2.96242E-08	33176725	34529900	1.000	0.000
hypoglycemia	hypothyroidism/myxoedema	rs1010473	6	90147159	6q15	rs1010473	ANKRD6	intronic	1.929	NA	2.00351E-09	89196632	91110879	0.113	0.031
hypoglycemia	hypothyroidism/myxoedema	rs1590257	6	166964294	6q27	rs1590257	RPS6KA2	intronic	0.787	7	1.20146E-09	165971017	167925120	0.088	0.068
hypoglycemia	hypothyroidism/myxoedema	rs443570	6	166976437	6q27	rs443570	RPS6KA2	intronic	4.456	NA	1.20146E-09	165971017	167925120	0.088	0.068
hypoglycemia	hypothyroidism/myxoedema	rs932635	11	35242423	11p13	rs932635	CD44	intronic	0.762	7	3.91909E-10	35237412	35244574	0.002	0.072

gout	hypothyroidism/myxoedema	rs4073682	1	108372838	1p13.3	rs4073682	VAV3	intronic	6.901	3a	1.13854E-09	108264355	108409665	0.000	0.000
gout	hypothyroidism/myxoedema	rs1967017	1	145723645	1q21.1	rs1967017	PDZK1	intergenic	15.25	2a	1.6428E-09	145719488	145725689	0.000	0.019
gout	hypothyroidism/myxoedema	rs7566808	2	191971155	2q32.2	rs7566808	STAT4	intronic	2.572	6	1.56501E-08	191943742	191971565	0.000	0.000
gout	hypothyroidism/myxoedema	rs4697698	4	9942577	4p16.1	rs4697698	SLC2A9	intronic	0.031	6	3.84977E-18	9918986	10416360	0.003	0.001
gout	hypothyroidism/myxoedema	rs12498742	4	9944052	4p16.1	rs12498742	SLC2A9	intronic	6.549	5	3.84977E-18	9918986	10416360	0.003	0.001
gout	hypothyroidism/myxoedema	rs4529048	4	9997112	4p16.1	rs4529048	SLC2A9	intronic	0.314	5	3.84977E-18	9918986	10416360	0.003	0.001
gout	hypothyroidism/myxoedema	rs7683792	4	10027969	4p16.1	rs7683792	SLC2A9	intronic	1.747	6	3.84977E-18	9918986	10416360	0.003	0.001
gout	hypothyroidism/myxoedema	rs6856396	4	10031163	4p16.1	rs6856396	SLC2A9	intronic	4.216	7	3.84977E-18	9918986	10416360	0.003	0.001

gout	hypothyroidism/myxoedema	rs17385112	4	10167532	4p16.1	rs17385112	AC006499.7	intergenic	1.621	5	3.84977E-18	9918986	10416360	0.003	0.001
gout	hypothyroidism/myxoedema	rs76748344	4	10311985	4p16.1	rs76748344	AC006499.1	intergenic	0.765	7	3.84977E-18	9918986	10416360	0.003	0.001
gout	hypothyroidism/myxoedema	rs4698030	4	10315850	4p16.1	rs4698030	AC006499.1	intergenic	2.68	7	3.84977E-18	9918986	10416360	0.003	0.001
gout	hypothyroidism/myxoedema	rs993173	4	10323935	4p16.1	rs993173	AC006499.1	intergenic	1.761	NA	3.84977E-18	9918986	10416360	0.003	0.001
gout	hypothyroidism/myxoedema	rs1860904	4	10357448	4p16.1	rs1860904	RP11-136I13.1	intergenic	0.845	NA	3.84977E-18	9918986	10416360	0.003	0.001
gout	hypothyroidism/myxoedema	rs10004908	4	88807246	4q22.1	rs10004908	HSP90AB3P	intergenic	1.601	7	6.57728E-19	88771611	89234813	0.019	0.001
gout	hypothyroidism/myxoedema	rs12510320	4	88810174	4q22.1	rs12510320	HSP90AB3P	intergenic	2.18	7	6.57728E-19	88771611	89234813	0.019	0.001
gout	hypothyroidism/myxoedema	rs17013547	4	88832568	4q22.1	rs17013547	HSP90AB3P	intergenic	0.506	4	6.57728E-19	88771611	89234813	0.019	0.001

gout	hypothyroidism/myxoedema	rs1463116	4	88856342	4q22.1	rs1463116	SPP1	intergenic	1.215	NA	6.57728E-19	88771611	89234813	0.019	0.001
gout	hypothyroidism/myxoedema	rs79653755	4	88860366	4q22.1	rs79653755	SPP1	intergenic	0.481	7	6.57728E-19	88771611	89234813	0.019	0.001
gout	hypothyroidism/myxoedema	rs5024096	4	88871276	4q22.1	rs5024096	SPP1	intergenic	2.65	2b	6.57728E-19	88771611	89234813	0.019	0.001
gout	hypothyroidism/myxoedema	rs6822046	4	88892117	4q22.1	rs6822046	SPP1	intergenic	3.002	6	6.57728E-19	88771611	89234813	0.019	0.001
gout	hypothyroidism/myxoedema	rs11938899	4	88909908	4q22.1	rs11938899	SPP1	intergenic	3.085	6	6.57728E-19	88771611	89234813	0.019	0.001
gout	hypothyroidism/myxoedema	rs2725236	4	88919106	4q22.1	rs2725236	RNU1-36P	intergenic	1.275	NA	6.57728E-19	88771611	89234813	0.019	0.001
gout	hypothyroidism/myxoedema	rs2725222	4	88958492	4q22.1	rs2725222	PKD2	intronic	1.777	NA	6.57728E-19	88771611	89234813	0.019	0.001
gout	hypothyroidism/myxoedema	rs2728098	4	88976008	4q22.1	rs2728098	PKD2	intronic	0.029	NA	6.57728E-19	88771611	89234813	0.019	0.001

gout	hypothyroidism/myxoedema	rs2725201	4	88999306	4q22.1	rs2725201	PKD2	downstream	0.78	NA	6.57728E-19	88771611	89234813	0.019	0.001
gout	hypothyroidism/myxoedema	rs2725269	4	89009006	4q22.1	rs2725269	ABCG2	intergenic	2.823	NA	6.57728E-19	88771611	89234813	0.019	0.001
gout	hypothyroidism/myxoedema	rs13120400	4	89033527	4q22.1	rs13120400	ABCG2	intronic	0.55	2b	6.57728E-19	88771611	89234813	0.019	0.001
gout	hypothyroidism/myxoedema	rs1481012	4	89039082	4q22.1	rs1481012	ABCG2	intronic	4.102	6	6.57728E-19	88771611	89234813	0.019	0.001
gout	hypothyroidism/myxoedema	rs3109823	4	89064602	4q22.1	rs3109823	ABCG2	intronic	7.204	NA	6.57728E-19	88771611	89234813	0.019	0.001
gout	hypothyroidism/myxoedema	rs2869736	4	89138377	4q22.1	rs2869736	ABCG2	intronic	9.554	4	6.57728E-19	88771611	89234813	0.019	0.001
gout	hypothyroidism/myxoedema	rs28793136	4	89216768	4q22.1	rs28793136	RP11-10L7.1	ncRNA_intronic	13	5	6.57728E-19	88771611	89234813	0.019	0.001
gout	hypothyroidism/myxoedema	rs9366622	6	25414537	6p22.2	rs9366622	LRRC16A	intronic	3.441	4	2.80876E-19	25414537	29607101	1.000	0.000

gout	hypothyroidism/myxoedema	rs13212936	6	25588815	6p22.2	rs13212936	LRRC16A	intronic	0.05	7	2.80876E-19	25414537	29607101	1.000	0.000
gout	hypothyroidism/myxoedema	rs68006638	6	25710571	6p22.2	rs68006638	SCGN	intergenic	0.616	6	2.80876E-19	25414537	29607101	1.000	0.000
gout	hypothyroidism/myxoedema	rs3936052	6	25715534	6p22.2	rs3936052	HIST1H2AA	intergenic	4.477	5	2.80876E-19	25414537	29607101	1.000	0.000
gout	hypothyroidism/myxoedema	rs1892252	6	25772639	6p22.2	rs1892252	SLC17A4	intronic	0.286	6	2.80876E-19	25414537	29607101	1.000	0.000
gout	hypothyroidism/myxoedema	rs13200784	6	25829633	6p22.2	rs13200784	SLC17A1	intronic	6.25	5	2.80876E-19	25414537	29607101	1.000	0.000
gout	hypothyroidism/myxoedema	rs1324088	6	25841122	6p22.2	rs1324088	SLC17A3	intronic	1.548	NA	2.80876E-19	25414537	29607101	1.000	0.000
gout	hypothyroidism/myxoedema	rs183879	6	25987441	6p22.2	rs183879	U91328.21	ncRNA_intronic	2.365	5	2.80876E-19	25414537	29607101	1.000	0.000
gout	hypothyroidism/myxoedema	rs34391493	6	26022648	6p22.2	rs34391493	HIST1H4A	downstream	2.876	3a	2.80876E-19	25414537	29607101	1.000	0.000

gout	hypothyroidism/myxoedema	rs6918586	6	26097384	6p22.2	rs6918586	HFE	UTR3	1.777	6	2.80876E-19	25414537	29607101	1.000	0.000
gout	hypothyroidism/myxoedema	rs9393686	6	26106601	6p22.2	rs9393686	HIST1H1T	intergenic	0.27	6	2.80876E-19	25414537	29607101	1.000	0.000
gout	hypothyroidism/myxoedema	rs34661691	6	26173478	6p22.2	rs34661691	HIST1H2BD	intergenic	4.645	5	2.80876E-19	25414537	29607101	1.000	0.000
gout	hypothyroidism/myxoedema	rs67575965	6	26196593	6p22.2	rs67575965	HIST1H3D	downstream	5.666	4	2.80876E-19	25414537	29607101	1.000	0.000
gout	hypothyroidism/myxoedema	rs1883215	6	26314233	6p22.2	rs1883215	HIST1H3PS1	intergenic	0.875	3a	2.80876E-19	25414537	29607101	1.000	0.000
gout	hypothyroidism/myxoedema	rs9379850	6	26343057	6p22.2	rs9379850	RNU6-1259P	intergenic	1.269	4	2.80876E-19	25414537	29607101	1.000	0.000
gout	hypothyroidism/myxoedema	rs9393718	6	26407482	6p22.2	rs9393718	BTN3A1	intronic	5.27	1d	2.80876E-19	25414537	29607101	1.000	0.000
gout	hypothyroidism/myxoedema	rs2273558	6	26466035	6p22.2	rs2273558	BTN2A1	intronic	3.628	7	2.80876E-19	25414537	29607101	1.000	0.000

gout	hypothyroidism/myxoedema	rs6924727	6	26491234	6p22.2	rs6924727	BTN1A1	intergenic	0.26	6	2.80876E-19	25414537	29607101	1.000	0.000
gout	hypothyroidism/myxoedema	rs183211374	6	26543212	6p22.2	rs183211374	HMGN4	intronic	0.885	6	2.80876E-19	25414537	29607101	1.000	0.000
gout	hypothyroidism/myxoedema	rs911186	6	27150599	6p22.2	rs911186	RP11-209A2.1	intergenic	11	NA	2.80876E-19	25414537	29607101	1.000	0.000
gout	hypothyroidism/myxoedema	rs7759741	6	27241043	6p22.1	rs7759741	XXbac-BPGBPG24018.1	intergenic	1.064	7	2.80876E-19	25414537	29607101	1.000	0.000
gout	hypothyroidism/myxoedema	rs7746199	6	27261324	6p22.1	rs7746199	POM121L2	intronic	0.879	1f	2.80876E-19	25414537	29607101	1.000	0.000
gout	hypothyroidism/myxoedema	rs2064219	6	27376001	6p22.1	rs2064219	MCFD2P1	downstream	11.4	6	2.80876E-19	25414537	29607101	1.000	0.000
gout	hypothyroidism/myxoedema	rs35984974	6	27410422	6p22.2	rs35984974	ZNF184	intergenic	1.104	6	2.80876E-19	25414537	29607101	1.000	0.000

gout	hypothyroidism/myxoedema	rs2056923	6	27689939	6p22.1	rs2056923	RP1-97D16.1	intergenic	7.067	5	2.80876E-19	25414537	29607101	1.000	0.000
gout	hypothyroidism/myxoedema	rs200956	6	27839746	6p22.1	rs200956	HIST1H3I	exonic	16.41	1f	2.80876E-19	25414537	29607101	1.000	0.000
gout	hypothyroidism/myxoedema	rs1736904	6	28219270	6p22.1	rs1736904	ZKSCAN4	intronic	12.41	NA	2.80876E-19	25414537	29607101	1.000	0.000
gout	hypothyroidism/myxoedema	rs853679	6	28296863	6p22.1	rs853679	ZSCAN31	intronic	8.065	NA	2.80876E-19	25414537	29607101	1.000	0.000
gout	hypothyroidism/myxoedema	rs7775835	6	28678357	6p22.1	rs7775835	RPSAP2	intergenic	1.117	4	2.80876E-19	25414537	29607101	1.000	0.000
gout	hypothyroidism/myxoedema	rs9393925	6	28682442	6p22.1	rs9393925	RPSAP2	intergenic	2.227	6	2.80876E-19	25414537	29607101	1.000	0.000
gout	hypothyroidism/myxoedema	rs1233578	6	28712247	6p22.1	rs1233578	RPSAP2	intergenic	6.017	7	2.80876E-19	25414537	29607101	1.000	0.000
gout	hypothyroidism/myxoedema	rs9257802	6	29343355	6p22.1	rs9257802	OR5V1	intronic	3.559	7	2.80876E-19	25414537	29607101	1.000	0.000

gout	hypothyroidism/myxoedema	rs1419636	6	29355702	6p22.1	rs1419636	OR5V1	intronic	1.865	NA	2.80876E-19	25414537	29607101	1.000	0.000
gout	hypothyroidism/myxoedema	rs1233478	6	29477821	6p22.1	rs1233478	XXbac-BPG13B8.10	ncRNA_intronic	0.714	NA	2.80876E-19	25414537	29607101	1.000	0.000
gout	hypothyroidism/myxoedema	rs1233410	6	29515494	6p22.1	rs1233410	OR2I1P	intergenic	8.305	3a	2.80876E-19	25414537	29607101	1.000	0.000
gout	hypothyroidism/myxoedema	rs1003582	6	29538403	6p22.2	rs1003582	GABBR1	intronic	2.571	7	2.80876E-19	25414537	29607101	1.000	0.000
gout	hypothyroidism/myxoedema	rs1233396	6	29546799	6p22.1	rs1233396	GABBR1	intronic	0.777	NA	2.80876E-19	25414537	29607101	1.000	0.000
gout	hypothyroidism/myxoedema	rs1704995	6	33187688	6p22.2	rs1704995	ZNF70P1	intergenic	8.782	NA	2.80876E-19	25414537	29607101	1.000	0.000
gout	hypothyroidism/myxoedema	rs418002	6	33223425	6p21.32	rs418002	VPS52	intronic	6.97	NA	5.67855E-11	33187688	33535941	0.001	0.001
gout	hypothyroidism/myxoedema	rs1061801	6	33282338	6p21.32	rs1061801	ZBTB22	UTR3	5.713	NA	5.67855E-11	33187688	33535941	0.001	0.001

gout	hypothyroidism/myxoedema	rs9277976	6	33292745	6p21.32	rs9277976	DAXX	intronic	11.13	5	5.67855E-11	33187688	33535941	0.001	0.001
gout	hypothyroidism/myxoedema	rs9278026	6	33339481	6p21.32	rs9278026	LYPLA2P1	intergenic	3.298	6	5.67855E-11	33187688	33535941	0.001	0.001
gout	hypothyroidism/myxoedema	rs3757247	6	90957463	6q15	rs3757247	BACH2	intronic	3.507	NA	8.36071E-10	90874672	91024294	0.000	0.000
gout	hypothyroidism/myxoedema	rs9111	6	90981653	6q15	rs9111	BACH2	UTR5	5.914	NA	8.36071E-10	90874672	91024294	0.000	0.000
gout	hypothyroidism/myxoedema	rs911409	6	91022755	6q15	rs911409	MIR4464	downstream	0.44	6	8.36071E-10	90874672	91024294	0.000	0.000
gout	hypothyroidism/myxoedema	rs927297	6	91024294	6q15	rs927297	MIR4464	intergenic	0.861	4	8.36071E-10	90874672	91024294	0.000	0.000
gout	hypothyroidism/myxoedema	rs2236313	6	167360389	6q27	rs2236313	RP11-514O12.4:RNASET2	intronic	3.329	6	1.64779E-08	167360389	167412048	0.000	0.000

gout	hypothyroidism/myxoedema	rs10760017	9	100687923	9q22.33	rs10760017	HEMGN	intergenic	5.112	3a	2.70522E-08	100687923	100687923	0.000	0.001
gout	hypothyroidism/myxoedema	rs12806363	11	64138447	11q13.1	rs12806363	RPS6KA4	intronic	4.84	6	6.58677E-09	56368708	56508409	0.000	0.000
gout	hypothyroidism/myxoedema	rs11231822	11	64338228	11q13.1	rs11231822	SLC22A11	intronic	0.47	5	3.73136E-12	64024056	64364866	1.000	0.000
gout	hypothyroidism/myxoedema	rs12363578	11	64364866	11q13.1	rs12363578	SLC22A12	intronic	0.09	5	3.73136E-12	64024056	64364866	1.000	0.000
gout	hypothyroidism/myxoedema	rs705696	12	56480648	12q13.2	rs705696	ERBB3	intronic	0.669	NA	6.58677E-09	56368708	56508409	0.001	0.003
gout	hypothyroidism/myxoedema	rs3759094	12	56497903	12q13.2	rs3759094	RP11-603J24.9	intronic	10.49	4	6.58677E-09	56368708	56508409	0.001	0.003
gout	hypothyroidism/myxoedema	rs11065822	12	111600134	12q24.11	rs11065822	CUX2	intronic	0.479	6	2.29213E-40	111426615	113218868	0.029	0.932
gout	hypothyroidism/myxoedema	rs886126	12	111679214	12q24.11	rs886126	CUX2	intronic	0.29	7	2.29213E-40	111426615	113218868	0.029	0.932

gout	hypothyroidism/myxoedema	rs1265564	12	111708458	12q24.11	rs1265564	CUX2	intronic	10.23	5	2.29213E-40	111426615	113218868	0.029	0.932
gout	hypothyroidism/myxoedema	rs7963641	12	111716718	12q24.11	rs7963641	CUX2	intronic	0.755	5	2.29213E-40	111426615	113218868	0.029	0.932
gout	hypothyroidism/myxoedema	rs933307	12	111735537	12q24.11	rs933307	CUX2	intronic	2.365	NA	2.29213E-40	111426615	113218868	0.029	0.932
gout	hypothyroidism/myxoedema	rs61507607	12	111767994	12q24.11	rs61507607	CUX2	intronic	4.237	5	2.29213E-40	111426615	113218868	0.029	0.932
gout	hypothyroidism/myxoedema	rs7299183	12	111771763	12q24.11	rs7299183	CUX2	intronic	0.309	7	2.29213E-40	111426615	113218868	0.029	0.932
gout	hypothyroidism/myxoedema	rs6490061	12	111773345	12q24.12	rs6490061	CUX2	intronic	4.933	6	2.29213E-40	111426615	113218868	0.029	0.932
gout	hypothyroidism/myxoedema	rs3809272	12	111800258	12q24.11	rs3809272	FAM109A	UTR3	1.548	4	2.29213E-40	111426615	113218868	0.029	0.932
gout	hypothyroidism/myxoedema	rs7398796	12	111814155	12q24.12	rs7398796	RP3-473L9.4	ncRNA_intronic	1.716	7	2.29213E-40	111426615	113218868	0.029	0.932

gout	hypothyroidism/myxoedema	rs10849941	12	111818487	12q24.12	rs10849941	RP3-473L9.4	ncRNA_intronic	6.138	5	2.29213E-40	111426615	113218868	0.029	0.932
gout	hypothyroidism/myxoedema	rs10774624	12	111833788	12q24.12	rs10774624	RP3-473L9.4	ncRNA_intronic	4.691	7	2.29213E-40	111426615	113218868	0.029	0.932
gout	hypothyroidism/myxoedema	rs11065898	12	111862575	12q24.12	rs11065898	SH2B3	intronic	0.269	4	2.29213E-40	111426615	113218868	0.029	0.932
gout	hypothyroidism/myxoedema	rs648997	12	111976776	12q24.12	rs648997	ATXN2	intronic	0.073	NA	2.29213E-40	111426615	113218868	0.029	0.932
gout	hypothyroidism/myxoedema	rs615134	12	112003695	12q24.12	rs615134	ATXN2	intronic	3.228	6	2.29213E-40	111426615	113218868	0.029	0.932
gout	hypothyroidism/myxoedema	rs4766897	12	112179471	12q24.12	rs4766897	ACAD10	intronic	1.631	2b	2.29213E-40	111426615	113218868	0.029	0.932
gout	hypothyroidism/myxoedema	rs7135514	12	112448485	12q24.12	rs7135514	TMEM116	intronic	4.906	3a	2.29213E-40	111426615	113218868	0.029	0.932
gout	hypothyroidism/myxoedema	rs4767364	12	112521448	12q24.12	rs4767364	NAA25	intronic	4.388	7	2.29213E-40	111426615	113218868	0.029	0.932

gout	hypothyroidism/myxoedema	rs7304572	12	112525073	12q24.12	rs7304572	NAA25	intronic	1.5	2c	2.29213E-40	111426615	113218868	0.029	0.932
gout	hypothyroidism/myxoedema	rs11066320	12	112906415	12q24.12	rs11066320	PTPN11	intronic	3.412	6	2.29213E-40	111426615	113218868	0.029	0.932
gout	hypothyroidism/myxoedema	rs233701	12	113012149	12q24.13	rs233701	RPH3A	intronic	0.714	2b	2.29213E-40	111426615	113218868	0.029	0.932
gout	hypothyroidism/myxoedema	rs233721	12	113031543	12q24.13	rs233721	RPH3A	intronic	0.998	6	2.29213E-40	111426615	113218868	0.029	0.932
gout	hypothyroidism/myxoedema	rs2891403	12	113137572	12q24.13	rs2891403	RPH3A	intronic	0.745	7	2.29213E-40	111426615	113218868	0.029	0.932
gout	hypothyroidism/myxoedema	rs6489855	12	113164767	12q24.13	rs6489855	RPH3A	intronic	3.14	7	2.29213E-40	111426615	113218868	0.029	0.932
gout	hypothyroidism/myxoedema	rs10850078	12	113209519	12q24.13	rs10850078	RPH3A	intronic	5.415	6	2.29213E-40	111426615	113218868	0.029	0.932
gout	hypothyroidism/myxoedema	rs28642975	12	122602305	12q24.31	rs28642975	MLXIP	intronic	0.917	7	9.87329E-10	122483836	122630285	0.058	0.447

gout	hypothyroidism/myxoedema	rs61759532	17	7240391	17p13.1	rs61759532	ACAP1	intronic	20.3	4	4.88656E-08	7226957	7240391	0.000	0.005
gout	hypothyroidism/myxoedema	rs6510827	19	4830628	19p13.3	rs6510827	TICAM1	intronic	3.279	4	1.79296E-08	4799850	4837557	0.000	0.001
gout	hypothyroidism/myxoedema	rs10405423	19	7211311	19p13.2	rs10405423	INSR	intronic	2.545	5	3.3709E-08	7211190	7219697	0.003	0.046
gout	hypothyroidism/myxoedema	rs112578407	22	37575469	22q12.3	rs112578407	RP1-151B14.6	ncRNA_intronic	0.526	5	3.70898E-08	37573712	37609342	0.000	0.000
hypothyroidism/myxoedema	hyperthyroidism/thyrotoxicosis	rs12033437	1	19749894	1p36.13	rs12033437	CAPZB	intronic	10.47	4	1.83E-09	19648143	19787354	0.008	0.047
hypothyroidism/myxoedema	hyperthyroidism/thyrotoxicosis	rs12128454	1	113910209	1p13.2	rs12128454	MAGI3	intergenic	7.752	6	9.15E-53	113871830	114537037	0.001	0.999
hypothyroidism/myxoedema	hyperthyroidism/thyrotoxicosis	rs1230666	1	114173410	1p13.2	rs1230666	MAGI3	intronic	2.8	NA	9.15E-53	113871830	114537037	0.001	0.999

hypothyroidism/myxoedema	hyperthyroidism/thyrotoxicosis	rs1230675	1	114175770	1p13.2	rs1230675	MAGI3	intronic	1.94	NA	9.15E-53	113871830	114537037	0.001	0.999
hypothyroidism/myxoedema	hyperthyroidism/thyrotoxicosis	rs1230661	1	114185590	1p13.2	rs1230661	MAGI3	intronic	10.51	6	9.15E-53	113871830	114537037	0.001	0.999
hypothyroidism/myxoedema	hyperthyroidism/thyrotoxicosis	rs1230682	1	114293526	1p13.2	rs1230682	PHTF1	intronic	5.653	NA	9.15E-53	113871830	114537037	0.001	0.999
hypothyroidism/myxoedema	hyperthyroidism/thyrotoxicosis	rs11102694	1	114426001	1p13.2	rs11102694	AP4B1-AS1:BCL2L15	ncRNA_intronic	5.406	6	9.15E-53	113871830	114537037	0.001	0.999
hypothyroidism/myxoedema	hyperthyroidism/thyrotoxicosis	rs10776775	1	114436482	1p13.2	rs10776775	AP4B1-AS1	ncRNA_intronic	4.615	6	9.15E-53	113871830	114537037	0.001	0.999
hypothyroidism/myxoedema	hyperthyroidism/thyrotoxicosis	rs1310183	1	114439522	1p13.2	rs1310183	AP4B1-AS1:AP4B1	ncRNA_intronic	6.069	6	9.15E-53	113871830	114537037	0.001	0.999

hypothyroidism/myxoedema	hyperthyroidism/thyrotoxicosis	rs10563713	1	114451425	1p13.2	rs10563713	DCLRE1B	intronic	1.784	NA	9.15E-53	113871830	114537037	0.001	0.999
hypothyroidism/myxoedema	hyperthyroidism/thyrotoxicosis	rs10563713	1	114451425	1p13.2	rs10563713	DCLRE1B	NA	NA	NA	9.15E-53	113871830	114537037	0.001	0.999
hypothyroidism/myxoedema	hyperthyroidism/thyrotoxicosis	rs12029644	1	114536780	1p13.2	rs12029644	RP4-590F24.1	intergenic	8.53	7	9.15E-53	113871830	114537037	0.001	0.999
hypothyroidism/myxoedema	hyperthyroidism/thyrotoxicosis	rs11264798	1	157661848	1q23.1	rs11264798	FCRL3:RP11-367J7.3	ncRNA_intronic	4.045	7	4.89E-08	157540981	157787253	0.063	0.137
hypothyroidism/myxoedema	hyperthyroidism/thyrotoxicosis	rs11675342	2	1407628	2p25.3	rs11675342	TPO	intergenic	0.828	7	5.47E-10	1400070	1417244	0.000	0.001
hypothyroidism/myxoedema	hyperthyroidism/thyrotoxicosis	rs10929817	2	12631916	2p24.3	rs10929817	AC096559.1	ncRNA_intronic	3.704	7	2.32E-11	12627163	12646111	0.002	0.218

hypothyroidism/myxoedema	hyperthyroidism/thyrotoxicosis	rs11679244	2	163082395	2q24.2	rs11679244	FAP	intronic	8.481	6	2.33E-09	163076146	163213723	0.000	0.008
hypothyroidism/myxoedema	hyperthyroidism/thyrotoxicosis	rs13023380	2	163154363	2q24.2	rs13023380	IFIH1	intronic	11.97	5	2.33E-09	163076146	163213723	0.000	0.008
hypothyroidism/myxoedema	hyperthyroidism/thyrotoxicosis	rs10168266	2	191935804	2q32.2	rs10168266	STAT4	intronic	0.746	6	1.72E-16	191900449	191973034	0.000	0.033
hypothyroidism/myxoedema	hyperthyroidism/thyrotoxicosis	rs11889341	2	191943742	2q32.2	rs11889341	STAT4	intronic	2.455	5	1.72E-16	191900449	191973034	0.000	0.033
hypothyroidism/myxoedema	hyperthyroidism/thyrotoxicosis	rs10207044	2	191958581	2q32.2	rs10207044	STAT4	intronic	2.037	NA	1.72E-16	191900449	191973034	0.000	0.033
hypothyroidism/myxoedema	hyperthyroidism/thyrotoxicosis	rs11681040	2	204551970	2q33.2	rs11681040	AC125238.3	intergenic	0.497	4	2.77E-64	204471201	204805387	0.002	0.998

hypothyroidism/myxoedema	hyperthyroidism/thyrotoxicosis	rs11676147	2	204555946	2q33.2	rs11676147	CD28	intergenic	5.128	3a	2.77E-64	204471201	204805387	0.002	0.998
hypothyroidism/myxoedema	hyperthyroidism/thyrotoxicosis	rs12693993	2	204595597	2q33.2	rs12693993	CD28	intronic	4.418	5	2.77E-64	204471201	204805387	0.002	0.998
hypothyroidism/myxoedema	hyperthyroidism/thyrotoxicosis	rs1024161	2	204721752	2q33.2	rs1024161	CTLA4	intergenic	13.58	5	2.77E-64	204471201	204805387	0.002	0.998
hypothyroidism/myxoedema	hyperthyroidism/thyrotoxicosis	rs11571302	2	204742934	2q33.2	rs11571302	CTLA4	intergenic	3.355	NA	2.77E-64	204471201	204805387	0.002	0.998
hypothyroidism/myxoedema	hyperthyroidism/thyrotoxicosis	rs11676461	2	204769396	2q33.2	rs11676461	CTLA4	intergenic	0.224	7	2.77E-64	204471201	204805387	0.002	0.998
hypothyroidism/myxoedema	hyperthyroidism/thyrotoxicosis	rs12616245	2	204770803	2q33.2	rs12616245	ICOS	intergenic	3.258	7	2.77E-64	204471201	204805387	0.002	0.998

hypothyroidism/myxoedema	hyperthyroidism/thyrotoxicosis	rs11571311	2	204773679	2q33.2	rs11571311	ICOS	intergenic	0.987	7	2.77E-64	204471201	204805387	0.002	0.998
hypothyroidism/myxoedema	hyperthyroidism/thyrotoxicosis	rs12464033	2	204796094	2q33.2	rs12464033	ICOS	intergenic	2.535	5	2.77E-64	204471201	204805387	0.002	0.998
hypothyroidism/myxoedema	hyperthyroidism/thyrotoxicosis	rs13090803	3	105934953	3q13.11	rs13090803	RP11-93B21.1	intergenic	2.145	6	1.42E-12	105916291	105975019	0.004	0.069
hypothyroidism/myxoedema	hyperthyroidism/thyrotoxicosis	rs13093110	3	188125120	3q28	rs13093110	LPP	intronic	8.718	5	2.13E-27	188072513	188135783	0.030	0.959
hypothyroidism/myxoedema	hyperthyroidism/thyrotoxicosis	rs12485444	3	188135783	3q28	rs12485444	LPP	intronic	13.87	5	2.13E-27	188072513	188135783	0.030	0.959
hypothyroidism/myxoedema	hyperthyroidism/thyrotoxicosis	rs11729055	4	10709726	4p16.1	rs11729055	RP11-61G19.1	intergenic	0.046	3b	2.4E-13	10701970	10774758	0.013	0.225

hypothyroidism/myxoedema	hyperthyroidism/thyrotoxicosis	rs11731191	4	10716979	4p16.1	rs11731191	RP11-61G19.1	intergenic	4.139	6	2.4E-13	10701970	10774758	0.013	0.225
hypothyroidism/myxoedema	hyperthyroidism/thyrotoxicosis	rs13110490	4	10727403	4p16.1	rs13110490	RP11-61G19.1	intergenic	0.505	7	2.4E-13	10701970	10774758	0.013	0.225
hypothyroidism/myxoedema	hyperthyroidism/thyrotoxicosis	rs13116306	4	10727697	4p16.1	rs13116306	RP11-61G19.1	intergenic	3.945	7	2.4E-13	10701970	10774758	0.013	0.225
hypothyroidism/myxoedema	hyperthyroidism/thyrotoxicosis	rs11933540	4	26120001	4p15.2	rs11933540	RP11-324H7.1	intergenic	4.144	5	1.48E-10	26085480	26128710	0.001	0.029
hypothyroidism/myxoedema	hyperthyroidism/thyrotoxicosis	rs13136820	4	40307564	4p14	rs13136820	AC195454.1	intergenic	3.997	4	1.48E-10	26085480	26128710	0.001	0.029
hypothyroidism/myxoedema	hyperthyroidism/thyrotoxicosis	rs10030849	4	149653099	4q31.23	rs10030849	ATP5LP4	intergenic	0.544	7	1.53E-11	40301264	40308368	0.153	0.288

hypothyroidism/myxoedema	hyperthyroidism/thyrotoxicosis	rs10213867	5	76533530	5q13.3	rs10213867	PDE8B	intronic	0.261	7	9.63E-10	76515824	76568292	0.001	0.015
hypothyroidism/myxoedema	hyperthyroidism/thyrotoxicosis	rs1050976	6	408079	6p25.3	rs1050976	IRF4	UTR3	3.508	4	1.46E-11	403799	421196	0.002	0.305
hypothyroidism/myxoedema	hyperthyroidism/thyrotoxicosis	rs13199775	6	25828782	6p22.2	rs13199775	SLC17A1	intronic	2.121	6	2.26E-45	25514179	29607101	0.927	0.073
hypothyroidism/myxoedema	hyperthyroidism/thyrotoxicosis	rs11966360	6	26316982	6p22.2	rs11966360	HIST1H3PS1	intergenic	6.554	5	2.26E-45	25514179	29607101	0.927	0.073
hypothyroidism/myxoedema	hyperthyroidism/thyrotoxicosis	rs12174631	6	26373150	6p22.2	rs12174631	BTN3A2	exonic	1.957	1f	2.26E-45	25514179	29607101	0.927	0.073
hypothyroidism/myxoedema	hyperthyroidism/thyrotoxicosis	rs13195047	6	26493384	6p22.2	rs13195047	BTN1A1	intergenic	0.376	4	2.26E-45	25514179	29607101	0.927	0.073

hypothyroidism/myxoedema	hyperthyroidism/thyrotoxicosis	rs13198716	6	26582035	6p22.2	rs13198716	ABT1	intergenic	12.54	1f	2.26E-45	25514179	29607101	0.927	0.073
hypothyroidism/myxoedema	hyperthyroidism/thyrotoxicosis	rs12204280	6	27033104	6p22.2	rs12204280	VN1R13P	intergenic	0.745	4	2.26E-45	25514179	29607101	0.927	0.073
hypothyroidism/myxoedema	hyperthyroidism/thyrotoxicosis	rs13194053	6	27143883	6p22.2	rs13194053	MIR3143	intergenic	3.548	1f	2.26E-45	25514179	29607101	0.927	0.073
hypothyroidism/myxoedema	hyperthyroidism/thyrotoxicosis	rs13211166	6	27265940	6p22.1	rs13211166	POM121L2	intronic	10.85	4	2.26E-45	25514179	29607101	0.927	0.073
hypothyroidism/myxoedema	hyperthyroidism/thyrotoxicosis	rs10946917	6	27380978	6p22.1	rs10946917	MCFD2P1	intergenic	0.172	7	2.26E-45	25514179	29607101	0.927	0.073
hypothyroidism/myxoedema	hyperthyroidism/thyrotoxicosis	rs113039233	6	27430126	6p22.1	rs113039233	ZNF184	intronic	0.257	6	2.26E-45	25514179	29607101	0.927	0.073

hypothyroidism/myxoedema	hyperthyroidism/thyrotoxicosis	rs12215012	6	27507494	6p22.1	rs12215012	HNRNPA1P1	intergenic	1.17	5	2.26E-45	25514179	29607101	0.927	0.073
hypothyroidism/myxoedema	hyperthyroidism/thyrotoxicosis	rs12179134	6	27675469	6p22.1	rs12179134	RP1-97D16.1	intergenic	5.906	6	2.26E-45	25514179	29607101	0.927	0.073
hypothyroidism/myxoedema	hyperthyroidism/thyrotoxicosis	rs10214440	6	27702440	6p22.1	rs10214440	RP1-97D16.1	intergenic	9.656	5	2.26E-45	25514179	29607101	0.927	0.073
hypothyroidism/myxoedema	hyperthyroidism/thyrotoxicosis	rs1150692	6	28173957	6p22.1	rs1150692	TOB2P1	intergenic	2.085	3a	2.26E-45	25514179	29607101	0.927	0.073
hypothyroidism/myxoedema	hyperthyroidism/thyrotoxicosis	rs10456362	6	28221816	6p22.1	rs10456362	ZKSCAN4	intronic	11.35	4	2.26E-45	25514179	29607101	0.927	0.073
hypothyroidism/myxoedema	hyperthyroidism/thyrotoxicosis	rs13210258	6	28308671	6p22.1	rs13210258	ZSCAN31	intronic	8.486	3a	2.26E-45	25514179	29607101	0.927	0.073

hypothyroidism/myxoedema	hyperthyroidism/thyrotoxicosis	rs13198809	6	28323702	6p22.1	rs13198809	ZSCAN31:ZKSCAN3	intronic	6.817	4	2.26E-45	25514179	29607101	0.927	0.073
hypothyroidism/myxoedema	hyperthyroidism/thyrotoxicosis	rs1233578	6	28712247	6p22.1	rs1233578	RPSAP2	intergenic	6.017	7	2.26E-45	25514179	29607101	0.927	0.073
hypothyroidism/myxoedema	hyperthyroidism/thyrotoxicosis	rs1233582	6	28714044	6p22.1	rs1233582	RPSAP2	intergenic	0.195	5	2.26E-45	25514179	29607101	0.927	0.073
hypothyroidism/myxoedema	hyperthyroidism/thyrotoxicosis	rs1073820	6	29132520	6p22.1	rs1073820	OR2J2	intergenic	4.715	NA	2.26E-45	25514179	29607101	0.927	0.073
hypothyroidism/myxoedema	hyperthyroidism/thyrotoxicosis	rs1233480	6	29477414	6p22.1	rs1233480	XXbac-BPG13B8.10	ncRNA_intronic	4.49	NA	2.26E-45	25514179	29607101	0.927	0.073
hypothyroidism/myxoedema	hyperthyroidism/thyrotoxicosis	rs1233478	6	29477821	6p22.1	rs1233478	XXbac-BPG13B8.10	ncRNA_intronic	0.714	NA	2.26E-45	25514179	29607101	0.927	0.073

hypothyroidism/myxoedema	hyperthyroidism/thyrotoxicosis	rs1015868	6	29485373	6p22.1	rs1015868	XXbac-BPG13B8.10	intergenic	2.253	NA	2.26E-45	25514179	29607101	0.927	0.073
hypothyroidism/myxoedema	hyperthyroidism/thyrotoxicosis	rs1233417	6	29511850	6p22.1	rs1233417	GPR53P	intergenic	0.791	NA	2.26E-45	25514179	29607101	0.927	0.073
hypothyroidism/myxoedema	hyperthyroidism/thyrotoxicosis	rs1233413	6	29513582	6p22.1	rs1233413	GPR53P	intergenic	1.32	7	2.26E-45	25514179	29607101	0.927	0.073
hypothyroidism/myxoedema	hyperthyroidism/thyrotoxicosis	rs1119080	6	29536685	6p22.1	rs1119080	GABBR1	intronic	1.238	6	2.26E-45	25514179	29607101	0.927	0.073
hypothyroidism/myxoedema	hyperthyroidism/thyrotoxicosis	rs1003582	6	29538403	6p22.1	rs1003582	GABBR1	intronic	2.571	7	2.26E-45	25514179	29607101	0.927	0.073
hypothyroidism/myxoedema	hyperthyroidism/thyrotoxicosis	rs1233397	6	29545715	6p22.1	rs1233397	GABBR1	intronic	1.387	NA	2.26E-45	25514179	29607101	0.927	0.073

hypothyroidism/myxoedema	hyperthyroidism/thyrotoxicosis	rs107822	6	33175575	6p21.32	rs107822	MIR219-1	upstream:downstream	11.89	1b	3.71E-20	33173842	33197589	0.548	0.000
hypothyroidism/myxoedema	hyperthyroidism/thyrotoxicosis	rs12199079	6	90852258	6q15	rs12199079	BACH2	intronic	2.742	6	2.01E-19	90809560	91014029	0.038	0.031
hypothyroidism/myxoedema	hyperthyroidism/thyrotoxicosis	rs10944479	6	90880393	6q15	rs10944479	BACH2	intronic	11.1	5	2.01E-19	90809560	91014029	0.038	0.031
hypothyroidism/myxoedema	hyperthyroidism/thyrotoxicosis	rs11755527	6	90958231	6q15	rs11755527	BACH2	intronic	3.55	6	2.01E-19	90809560	91014029	0.038	0.031
hypothyroidism/myxoedema	hyperthyroidism/thyrotoxicosis	rs1130033	6	167385623	6q27	rs1130033	RP1-167A14.2	ncRNA_intronic	0.564	NA	8.88E-21	167366394	167523395	0.073	0.847
hypothyroidism/myxoedema	hyperthyroidism/thyrotoxicosis	rs12526548	6	167431147	6q27	rs12526548	FGFR1OP:RP11-517H2.6	ncRNA_intronic	3.733	7	8.88E-21	167366394	167523395	0.073	0.847

hypothyroidism/myxoedema	hyperthyroidism/thyrotoxicosis	rs118155381	8	128997208	8q24.21	rs118155381	PVT1	ncRNA_intronic	1.291	6	6.92E-12	128994897	129015313	0.011	0.982
hypothyroidism/myxoedema	hyperthyroidism/thyrotoxicosis	rs10087240	8	129012574	8q24.21	rs10087240	PVT1	ncRNA_intronic	1.698	7	6.92E-12	128994897	129015313	0.011	0.982
hypothyroidism/myxoedema	hyperthyroidism/thyrotoxicosis	rs11781004	8	134212130	8q24.22	rs11781004	WISP1	intronic	0.603	5	1.5E-13	134209453	134219818	0.005	0.992
hypothyroidism/myxoedema	hyperthyroidism/thyrotoxicosis	rs10965064	9	21553538	9p21.3	rs10965064	MIR31HG	ncRNA_intronic	0.747	4	2.79E-09	21552052	21585265	0.000	0.003
hypothyroidism/myxoedema	hyperthyroidism/thyrotoxicosis	rs11256311	10	6049313	10p15.1	rs11256311	IL2RA	intergenic	9.329	5	3.69E-16	6035291	6185310	0.001	0.998
hypothyroidism/myxoedema	hyperthyroidism/thyrotoxicosis	rs11256442	10	6079344	10p15.1	rs11256442	IL2RA	intronic	4.473	4	3.69E-16	6035291	6185310	0.001	0.998

hypothyroidism/myxoedema	hyperthyroidism/thyrotoxicosis	rs12253981	10	6092346	10p15.1	rs12253981	IL2RA	intronic	8.179	2b	3.69E-16	6035291	6185310	0.001	0.998
hypothyroidism/myxoedema	hyperthyroidism/thyrotoxicosis	rs11256516	10	6092444	10p15.1	rs11256516	IL2RA	intronic	2.51	5	3.69E-16	6035291	6185310	0.001	0.998
hypothyroidism/myxoedema	hyperthyroidism/thyrotoxicosis	rs11597367	10	6107534	10p15.1	rs11597367	IL2RA	intergenic	0.011	5	3.69E-16	6035291	6185310	0.001	0.998
hypothyroidism/myxoedema	hyperthyroidism/thyrotoxicosis	rs10795791	10	6108340	10p15.1	rs10795791	IL2RA	intergenic	4.359	5	3.69E-16	6035291	6185310	0.001	0.998
hypothyroidism/myxoedema	hyperthyroidism/thyrotoxicosis	rs10905716	10	6114010	10p15.1	rs10905716	RP11-414H17.2	upstream	2.399	2c	3.69E-16	6035291	6185310	0.001	0.998
hypothyroidism/myxoedema	hyperthyroidism/thyrotoxicosis	rs10905719	10	6115171	10p15.1	rs10905719	RP11-414H17.2	intergenic	0.008	7	3.69E-16	6035291	6185310	0.001	0.998

hypothyroidism/myxoedema	hyperthyroidism/thyrotoxicosis	rs11598494	10	6178941	10p15.1	rs11598494	PFKFB3	intergenic	5.754	4	3.69E-16	6035291	6185310	0.001	0.998
hypothyroidism/myxoedema	hyperthyroidism/thyrotoxicosis	rs11593331	10	6184190	10p15.1	rs11593331	PFKFB3	intergenic	1.186	4	3.69E-16	6035291	6185310	0.001	0.998
hypothyroidism/myxoedema	hyperthyroidism/thyrotoxicosis	rs12761779	10	63782043	10q21.2	rs12761779	ARID5B	intronic	1.64	5	1.29E-19	63779871	64057202	0.003	0.978
hypothyroidism/myxoedema	hyperthyroidism/thyrotoxicosis	rs12781780	10	63790794	10q21.2	rs12781780	ARID5B	intronic	1.526	7	1.29E-19	63779871	64057202	0.003	0.978
hypothyroidism/myxoedema	hyperthyroidism/thyrotoxicosis	rs10821948	10	63809624	10q21.2	rs10821948	ARID5B	intronic	3.399	3a	1.29E-19	63779871	64057202	0.003	0.978
hypothyroidism/myxoedema	hyperthyroidism/thyrotoxicosis	rs10761616	10	64009076	10q21.2	rs10761616	RTKN2	intronic	15.21	7	1.29E-19	63779871	64057202	0.003	0.978

hypothyroidism/myxoedema	hyperthyroidism/thyrotoxicosis	rs10831382	11	95318180	11q21	rs10831382	RP11-338H14.1	intergenic	2.43	6	3.15E-21	95298828	95327374	0.000	1.000
hypothyroidism/myxoedema	hyperthyroidism/thyrotoxicosis	rs11021232	11	95320808	11q21	rs11021232	RP11-338H14.1	intergenic	1.81	5	3.15E-21	95298828	95327374	0.000	1.000
hypothyroidism/myxoedema	hyperthyroidism/thyrotoxicosis	rs12418638	11	128163457	11q24.3	rs12418638	RP11-702B10.1	intergenic	0.014	7	2.83E-08	128131013	128178798	0.005	0.295
hypothyroidism/myxoedema	hyperthyroidism/thyrotoxicosis	rs11065784	12	111412463	12q24.11	rs11065784	RP1-46F2.2	intergenic	0.138	7	1.73E-36	111359712	112985328	0.008	0.943
hypothyroidism/myxoedema	hyperthyroidism/thyrotoxicosis	rs10849925	12	111495518	12q24.11	rs10849925	CUX2	intronic	16.4	4	1.73E-36	111359712	112985328	0.008	0.943
hypothyroidism/myxoedema	hyperthyroidism/thyrotoxicosis	rs10774616	12	111662885	12q24.11	rs10774616	CUX2	intronic	2.738	5	1.73E-36	111359712	112985328	0.008	0.943

hypothyroidism/myxoedema	hyperthyroidism/thyrotoxicosis	rs11065837	12	111684253	12q24.11	rs11065837	CUX2	intronic	0.581	6	1.73E-36	111359712	112985328	0.008	0.943
hypothyroidism/myxoedema	hyperthyroidism/thyrotoxicosis	rs1265564	12	111708458	12q24.11	rs1265564	CUX2	intronic	10.23	5	1.73E-36	111359712	112985328	0.008	0.943
hypothyroidism/myxoedema	hyperthyroidism/thyrotoxicosis	rs11611514	12	111718417	12q24.11	rs11611514	CUX2	intronic	0.592	5	1.73E-36	111359712	112985328	0.008	0.943
hypothyroidism/myxoedema	hyperthyroidism/thyrotoxicosis	rs11065857	12	111739500	12q24.11	rs11065857	CUX2	intronic	2.89	7	1.73E-36	111359712	112985328	0.008	0.943
hypothyroidism/myxoedema	hyperthyroidism/thyrotoxicosis	rs10849932	12	111755901	12q24.11	rs10849932	CUX2	intronic	4.464	7	1.73E-36	111359712	112985328	0.008	0.943
hypothyroidism/myxoedema	hyperthyroidism/thyrotoxicosis	rs10849937	12	111792427	12q24.12	rs10849937	CUX2	intergenic	1.172	3a	1.73E-36	111359712	112985328	0.008	0.943

hypothyroidism/myxoedema	hyperthyroidism/thyrotoxicosis	rs10849943	12	111819589	12q24.12	rs10849943	RP3-473L9.4	ncRNA_intronic	1.609	7	1.73E-36	111359712	112985328	0.008	0.943
hypothyroidism/myxoedema	hyperthyroidism/thyrotoxicosis	rs10774625	12	111910219	12q24.12	rs10774625	ATXN2	intronic	2.748	5	1.73E-36	111359712	112985328	0.008	0.943
hypothyroidism/myxoedema	hyperthyroidism/thyrotoxicosis	rs11065961	12	112023067	12q24.12	rs11065961	ATXN2	intronic	0.366	2b	1.73E-36	111359712	112985328	0.008	0.943
hypothyroidism/myxoedema	hyperthyroidism/thyrotoxicosis	rs10744774	12	112090022	12q24.12	rs10744774	BRAP	intronic	3.377	7	1.73E-36	111359712	112985328	0.008	0.943
hypothyroidism/myxoedema	hyperthyroidism/thyrotoxicosis	rs11066085	12	112355949	12q24.12	rs11066085	ADAM1B	intergenic	1.58	6	1.73E-36	111359712	112985328	0.008	0.943
hypothyroidism/myxoedema	hyperthyroidism/thyrotoxicosis	rs113703611	14	81133909	14q31.1	rs113703611	CEP128	intronic	4.926	6	8.66E-11	81127548	81490742	0.003	0.001

hypothyroidism/myxoedema	hyperthyroidism/thyrotoxicosis	rs1031299	14	81170137	14q31.1	rs1031299	CEP128	intronic	2.267	5	8.66E-11	81127548	81490742	0.003	0.001
hypothyroidism/myxoedema	hyperthyroidism/thyrotoxicosis	rs12896436	14	81382533	14q31.1	rs12896436	CEP128	intronic	2.262	4	8.66E-11	81127548	81490742	0.003	0.001
hypothyroidism/myxoedema	hyperthyroidism/thyrotoxicosis	rs10146516	14	81414747	14q31.1	rs10146516	CEP128	intronic	5.14	7	8.66E-11	81127548	81490742	0.003	0.001
hypothyroidism/myxoedema	hyperthyroidism/thyrotoxicosis	rs112069648	14	81461632	14q31.1	rs112069648	TSHR	intronic	3.535	7	8.66E-11	81127548	81490742	0.003	0.001
hypothyroidism/myxoedema	hyperthyroidism/thyrotoxicosis	rs1023586	14	81462283	14q31.1	rs1023586	TSHR	intronic	5.135	6	8.66E-11	81127548	81490742	0.003	0.001
hypothyroidism/myxoedema	hyperthyroidism/thyrotoxicosis	rs11631591	15	38850262	15q14	rs11631591	RASGRP1	intronic	1.844	3a	1.43E-13	38820606	38909464	0.012	0.541

hypothyroidism/myxoedema	hyperthyroidism/thyrotoxicosis	rs12325861	17	40289412	17q21.2	rs12325861	CTD-2132N18.3:RAB5C	intronic	1.754	6	8.91E-10	40271970	40300126	0.000	0.071
hypothyroidism/myxoedema	hyperthyroidism/thyrotoxicosis	rs10422141	19	4833410	19p13.3	rs10422141	TICAM1	intergenic	0.032	5	1.62E-18	4789176	4838056	0.007	0.980
hypothyroidism/myxoedema	hyperthyroidism/thyrotoxicosis	rs10424978	19	4837557	19p13.3	rs10424978	CTC-518P12.6	upstream:downstream	3.465	4	1.62E-18	4789176	4838056	0.007	0.980
hypothyroidism/myxoedema	hyperthyroidism/thyrotoxicosis	rs12482947	21	43852037	21q22.3	rs12482947	UBASH3A	intronic	2.756	5	1.22E-08	43852037	43852037	0.000	0.074
hypothyroidism/myxoedema	hyperthyroidism/thyrotoxicosis	rs1003342	22	30570022	22q12.2	rs1003342	HORMAD2	intronic	6.356	6	3.73E-08	30223888	30592487	0.001	0.011
hypothyroidism/myxoedema	hyperthyroidism/thyrotoxicosis	rs112578407	22	37575469	22q12.3	rs112578407	RP1-151B14.6	ncRNA_intronic	0.526	5	1.83E-12	37573712	37609342	0.001	0.028

Analysis of MAGMA

Trait pair	Trait pair	GENE	CHR	START	STOP	NSNPS	NPARAM	N	ZSTAT	P	SYMBOL
Type 2 diabetes	hypoglycemia	ENSG00000081026	1	113933371	114228545	3	1	817867	5.69	6.1855E-09	MAGI3
Type 2 diabetes	hypoglycemia	ENSG00000116793	1	114239453	114302111	5	1	817867	5.42	2.9811E-08	PHTF1
Type 2 diabetes	hypoglycemia	ENSG00000081019	1	114304454	114355098	2	1	817867	5.7	5.8479E-09	RSBN1
Type 2 diabetes	hypoglycemia	ENSG00000134242	1	114356433	114414381	5	1	817867	6.11	5E-10	PTPN22
Type 2 diabetes	hypoglycemia	ENSG00000188761	1	114420790	114430169	1	1	817867	5.36	4.1313E-08	BCL2L15
Type 2 diabetes	hypoglycemia	ENSG00000084734	2	27719709	27746554	2	1	817867	4.91	4.6163E-07	GCKR
Type 2 diabetes	hypoglycemia	ENSG00000132170	3	12328867	12475855	26	1	817867	6.04	7.5819E-10	PPARG
Type 2 diabetes	hypoglycemia	ENSG00000173175	3	123001143	123168605	5	1	817867	5.64	8.6201E-09	ADCY5
Type 2 diabetes	hypoglycemia	ENSG00000073792	3	185361527	185542844	60	1	817867	6.11	5E-10	IGF2BP2

Type 2 diabetes	hypoglycemia	ENSG00000109501	4	6271576	6304992	96	2	817867	6.29	1.6353E-10	WFS1
Type 2 diabetes	hypoglycemia	ENSG00000109670	4	153242410	153457253	7	1	817867	4.38	6.0367E-06	FBXW7
Type 2 diabetes	hypoglycemia	ENSG00000225940	5	55807394	55902059	6	1	817867	5.52	1.6788E-08	AC022431.2
Type 2 diabetes	hypoglycemia	ENSG00000145730	5	102089685	102366809	1	1	817867	5.76	4.3146E-09	PAM
Type 2 diabetes	hypoglycemia	ENSG00000124783	6	7268539	7347679	2	1	817867	5.35	4.415E-08	SSR1
Type 2 diabetes	hypoglycemia	ENSG00000145996	6	20534688	21232635	83	4	817867	6.11	5E-10	CDKAL1
Type 2 diabetes	hypoglycemia	ENSG00000204540	6	31082527	31107869	22	5	817867	7.81	2.8311E-15	PSORS1C1
Type 2 diabetes	hypoglycemia	ENSG00000204539	6	31082867	31088223	3	1	817867	5.83	2.8531E-09	CDSN
Type 2 diabetes	hypoglycemia	ENSG00000204538	6	31105313	31107127	4	2	817867	6.94	2.0234E-12	PSORS1C2
Type 2 diabetes	hypoglycemia	ENSG00000204516	6	31462658	31478901	54	5	817867	6.7	1.0365E-11	MICB

Type 2 diabetes	hypoglycemia	ENSG00000198563	6	31497996	31510225	2	1	817867	6.11	5E-10	DDX39B
Type 2 diabetes	hypoglycemia	ENSG00000254870	6	31497996	31514385	8	2	817867	6.81	4.9836E-12	ATP6V1G2-DDX39B
Type 2 diabetes	hypoglycemia	ENSG00000213760	6	31512239	31516204	6	2	817867	7.75	4.6629E-15	ATP6V1G2
Type 2 diabetes	hypoglycemia	ENSG00000204498	6	31514647	31526606	9	3	817867	8.1	2.7756E-16	NFKBIL1
Type 2 diabetes	hypoglycemia	ENSG00000226979	6	31539831	31542101	5	1	817867	6.48	4.5809E-11	LTA
Type 2 diabetes	hypoglycemia	ENSG00000204482	6	31553901	31556686	2	1	817867	6.11	5E-10	LST1
Type 2 diabetes	hypoglycemia	ENSG00000204475	6	31556672	31560762	5	1	817867	6.58	2.3827E-11	NCR3
Type 2 diabetes	hypoglycemia	ENSG00000213719	6	31698358	31707540	2	1	817867	8.29	5.778E-17	CLIC1
Type 2 diabetes	hypoglycemia	ENSG00000204410	6	31707725	31732622	14	2	817867	6.11	5E-10	MSH5
Type 2 diabetes	hypoglycemia	ENSG00000255152	6	31707797	31732628	14	2	817867	6.11	5E-10	MSH5-SAPCD1

Type 2 diabetes	hypoglycemia	ENSG00000204390	6	31777396	31783437	2	1	817867	6.11	5E-10	HSPA1L
Type 2 diabetes	hypoglycemia	ENSG00000204385	6	31830969	31846823	23	3	817867	7.72	5.7176E-15	SLC44A4
Type 2 diabetes	hypoglycemia	ENSG00000204371	6	31847536	31865464	1	1	817867	7.29	1.4956E-13	EHMT2
Type 2 diabetes	hypoglycemia	ENSG00000166278	6	31865562	31913449	2	1	817867	6.11	5E-10	C2
Type 2 diabetes	hypoglycemia	ENSG00000244255	6	31895475	31919825	3	2	817867	6.19	3.0471E-10	CFB
Type 2 diabetes	hypoglycemia	ENSG00000243649	6	31895475	31919861	3	2	817867	6.19	3.0471E-10	CFB
Type 2 diabetes	hypoglycemia	ENSG00000204344	6	31938868	31950598	2	1	817867	4.29	0.000009083	STK19
Type 2 diabetes	hypoglycemia	ENSG00000168477	6	32008931	32083111	23	4	817867	7.96	8.8818E-16	TNXB
Type 2 diabetes	hypoglycemia	ENSG00000213676	6	32065953	32096030	7	3	817867	6.11	5E-10	ATF6B
Type 2 diabetes	hypoglycemia	ENSG00000179344	6	32627244	32636160	2	1	817867	6.11	5E-10	HLA-DQB1

Type 2 diabetes	hypoglycemia	ENSG00000241106	6	32780540	32784825	6	3	817867	6.11	5E-10	HLA-DOB
Type 2 diabetes	hypoglycemia	ENSG00000250264	6	32781544	32806599	56	8	817867	6.11	5E-10	TAP2
Type 2 diabetes	hypoglycemia	ENSG00000204267	6	32789610	32806557	37	7	817867	6.11	5E-10	TAP2
Type 2 diabetes	hypoglycemia	ENSG00000204264	6	32808494	32812480	5	1	817867	6.49	4.2289E-11	PSMB8
Type 2 diabetes	hypoglycemia	ENSG00000240065	6	32811913	32827362	7	2	817867	7.71	6.1617E-15	PSMB9
Type 2 diabetes	hypoglycemia	ENSG00000168394	6	32812986	32821755	6	2	817867	6.11	5E-10	TAP1
Type 2 diabetes	hypoglycemia	ENSG00000204252	6	32971955	32977389	1	1	817867	6.32	1.318E-10	HLA-DOA
Type 2 diabetes	hypoglycemia	ENSG00000030110	6	33540329	33548019	2	1	817867	5.44	2.6683E-08	BAK1
Type 2 diabetes	hypoglycemia	ENSG00000204188	6	33551515	33556803	3	1	817867	5.9	1.8022E-09	GGNBP1
Type 2 diabetes	hypoglycemia	ENSG00000203760	6	126661320	126670021	3	1	817867	3.69	0.00011061	CENPW

Type 2 diabetes	hypoglycemia	ENSG00000153814	7	27870192	28220362	28	3	817867	6.96	1.7145E-12	JAZF1
Type 2 diabetes	hypoglycemia	ENSG00000009335	7	156931607	157062066	3	1	817867	5.57	1.2849E-08	UBE3C
Type 2 diabetes	hypoglycemia	ENSG00000029534	8	41510739	41754280	13	1	817867	5.76	4.2565E-09	ANK1
Type 2 diabetes	hypoglycemia	ENSG00000164756	8	117962512	118188953	3	1	817867	5.45	2.4975E-08	SLC30A8
Type 2 diabetes	hypoglycemia	ENSG00000151465	10	12237964	12292588	1	1	817867	5.37	4.038E-08	CDC123
Type 2 diabetes	hypoglycemia	ENSG00000119912	10	94211441	94333833	11	1	817867	4.86	5.8571E-07	IDE
Type 2 diabetes	hypoglycemia	ENSG00000138160	10	94353043	94415150	29	2	817867	5.59	1.1521E-08	KIF11
Type 2 diabetes	hypoglycemia	ENSG00000152804	10	94447945	94455403	1	1	817867	6.11	5.0575E-10	HHEX
Type 2 diabetes	hypoglycemia	ENSG00000148737	10	114710009	114927437	86	5	817867	7.6	1.4322E-14	TCF7L2
Type 2 diabetes	hypoglycemia	ENSG00000188211	11	17373273	17398888	6	1	817867	6.11	5E-10	NCR3LG1

Type 2 diabetes	hypoglycemia	ENSG00000187486	11	17407406	17410878	3	1	817867	5.07	2.0169E-07	KCNJ11
Type 2 diabetes	hypoglycemia	ENSG00000006071	11	17414432	17498449	4	1	817867	5.89	1.8896E-09	ABCC8
Type 2 diabetes	hypoglycemia	ENSG00000186635	11	72396114	72504644	15	1	817867	5.56	1.3458E-08	ARAP1
Type 2 diabetes	hypoglycemia	ENSG00000214530	11	72465774	72504726	4	1	817867	6.53	3.3407E-11	STARD10
Type 2 diabetes	hypoglycemia	ENSG00000137478	11	72547790	72853306	4	1	817867	4.09	0.000021587	FCHSD2
Type 2 diabetes	hypoglycemia	ENSG00000118971	12	4382938	4414516	1	1	817867	5.71	5.7318E-09	CCND2
Type 2 diabetes	hypoglycemia	ENSG00000205693	12	27915671	27924209	14	1	817867	5.55	1.4154E-08	MANSC4
Type 2 diabetes	hypoglycemia	ENSG00000087448	12	27932953	27955973	7	1	817867	5.83	2.7285E-09	KLHL42
Type 2 diabetes	hypoglycemia	ENSG00000149948	12	66217911	66360075	3	1	817867	4.88	5.3674E-07	HMGA2
Type 2 diabetes	hypoglycemia	ENSG00000135100	12	121416346	121440315	2	1	817867	5.88	2.0326E-09	HNF1A

Type 2 diabetes	hypoglycemia	ENSG00000140718	16	53737875	54155853	104	2	817867	6.11	5E-10	FTO
Type 2 diabetes	osteoporosis	ENSG00000084734	2	27719709	27746554	1	1	1118599	6.81	5.03E-12	GCKR
Type 2 diabetes	osteoporosis	ENSG00000132170	3	12328867	12475855	4	1	1118599	4.05	0.000025768	PPARG
Type 2 diabetes	osteoporosis	ENSG00000073792	3	185361527	185542844	7	1	1118599	6.04	7.791E-10	IGF2BP2
Type 2 diabetes	osteoporosis	ENSG00000225940	5	55807394	55902059	1	1	1118599	5.36	4.13E-08	AC022431.2
Type 2 diabetes	osteoporosis	ENSG00000145996	6	20534688	21232635	1	1	1118599	5.36	4.15E-08	CDKAL1
Type 2 diabetes	osteoporosis	ENSG00000148737	10	114710009	114927437	20	2	1118599	6.88	2.9792E-12	TCF7L2
Type 2 diabetes	osteoporosis	ENSG00000134640	11	92702886	92718232	1	1	1118599	5.4	3.33E-08	MTNR1B
Type 2 diabetes	osteoporosis	ENSG00000198901	15	91509270	91538859	1	1	1118599	5.55	1.41E-08	PRC1
Type 2 diabetes	osteoporosis	ENSG00000140718	16	53737875	54155853	16	1	1118599	7.82	2.5535E-15	FTO

Type 2 diabetes	gout	ENSG00000115204	2	27532360	27548547	1	1	1118599	6.87	3.3102E-12	MPV17
Type 2 diabetes	gout	ENSG00000115207	2	27548716	27579868	10	1	1118599	7.3	1.4433E-13	GTF3C2
Type 2 diabetes	gout	ENSG00000115211	2	27587219	27593353	2	1	1118599	6.11	5E-10	EIF2B4
Type 2 diabetes	gout	ENSG00000115234	2	27593389	27599995	4	1	1118599	5.77	3.9021E-09	SNX17
Type 2 diabetes	gout	ENSG00000163795	2	27600098	27603657	1	1	1118599	7.23	2.4046E-13	ZNF513
Type 2 diabetes	gout	ENSG00000115241	2	27604061	27632554	17	1	1118599	6.99	1.4067E-12	PPM1G
Type 2 diabetes	gout	ENSG00000115216	2	27650657	27665126	11	1	1118599	6.11	5E-10	NRBP1
Type 2 diabetes	gout	ENSG00000157992	2	27665233	27669348	2	1	1118599	4.99	3.0443E-07	KRTCAP3
Type 2 diabetes	gout	ENSG00000138002	2	27667238	27712656	32	1	1118599	5.47	2.2631E-08	IFT172
Type 2 diabetes	gout	ENSG00000115226	2	27714750	27718112	4	1	1118599	5.62	9.5256E-09	FNDC4

Type 2 diabetes	gout	ENSG00000084734	2	27719709	27746554	23	2	1118599	6.29	1.6323E-10	GCKR
Type 2 diabetes	gout	ENSG00000233438	2	27760253	27790011	9	1	1118599	6.11	5E-10	AC109829.1
Type 2 diabetes	gout	ENSG00000221843	2	27799389	27805588	2	1	1118599	7.87	1.8355E-15	C2orf16
Type 2 diabetes	gout	ENSG00000243943	2	27805897	27858041	18	1	1118599	6.92	2.2619E-12	ZNF512
Type 2 diabetes	gout	ENSG00000198522	2	27851114	27874375	4	1	1118599	5.76	4.0851E-09	GPN1
Type 2 diabetes	gout	ENSG00000163798	2	27886338	27917840	3	1	1118599	5.86	2.2568E-09	SLC4A1AP
Type 2 diabetes	gout	ENSG00000205334	2	27928653	27938599	4	1	1118599	5.53	1.596E-08	AC074091.13
Type 2 diabetes	gout	ENSG00000082438	2	165510134	165700189	3	1	1118599	4.19	0.000013912	COBLL1
Type 2 diabetes	gout	ENSG00000132170	3	12328867	12475855	26	1	1118599	6.73	8.5778E-12	PPARG
Type 2 diabetes	gout	ENSG00000163935	3	52937588	53080766	1	1	1118599	5.42	2.9387E-08	SFMBT1

Type 2 diabetes	gout	ENSG00000272305	3	53003135	53133469	26	1	1118599	5.57	1.2436E-08	RP11-894J14.5
Type 2 diabetes	gout	ENSG00000163933	3	53122499	53164478	9	1	1118599	4.19	0.000013844	RFT1
Type 2 diabetes	gout	ENSG00000158186	3	138066539	138124375	2	1	1118599	3.89	0.000050078	MRAS
Type 2 diabetes	gout	ENSG00000073792	3	185361527	185542844	42	1	1118599	5.07	2.0165E-07	IGF2BP2
Type 2 diabetes	gout	ENSG00000109667	4	9772777	10056560	119	7	1118599	6.11	5E-10	SLC2A9
Type 2 diabetes	gout	ENSG00000071127	4	10075963	10118573	6	1	1118599	6.3	1.533E-10	WDR1
Type 2 diabetes	gout	ENSG00000178163	4	10441498	10459034	3	1	1118599	6.34	1.1212E-10	ZNF518B
Type 2 diabetes	gout	ENSG00000109684	4	10488019	10686489	23	2	1118599	6.12	4.6444E-10	CLNK
Type 2 diabetes	gout	ENSG00000163644	4	89178772	89205921	3	1	1118599	5.55	1.3897E-08	PPM1K
Type 2 diabetes	gout	ENSG00000225940	5	55807394	55902059	1	1	1118599	5.53	1.5831E-08	AC022431.2

Type 2 diabetes	gout	ENSG00000124782	6	7107830	7252213	8	1	1118599	5.35	4.3449E-08	RREB1
Type 2 diabetes	gout	ENSG00000145996	6	20534688	21232635	11	1	1118599	4.93	4.0631E-07	CDKAL1
Type 2 diabetes	gout	ENSG00000079689	6	25652464	25702011	2	1	1118599	4.97	3.3701E-07	SCGN
Type 2 diabetes	gout	ENSG00000146039	6	25754927	25781419	9	1	1118599	6.35	1.072E-10	SLC17A4
Type 2 diabetes	gout	ENSG00000124568	6	25783125	25832287	37	2	1118599	6.51	3.7814E-11	SLC17A1
Type 2 diabetes	gout	ENSG00000124564	6	25833294	25882514	22	1	1118599	5.7	5.9849E-09	SLC17A3
Type 2 diabetes	gout	ENSG00000226979	6	31539831	31542101	2	1	1118599	4.14	0.000017411	LTA
Type 2 diabetes	gout	ENSG00000146477	6	160769300	160876014	16	1	1118599	4.16	0.000016243	SLC22A3
Type 2 diabetes	gout	ENSG00000009954	7	72854728	72936608	17	1	1118599	6.42	6.648E-11	BAZ1B
Type 2 diabetes	gout	ENSG00000106635	7	72950686	72972332	1	1	1118599	6.98	1.5051E-12	BCL7B

Type 2 diabetes	gout	ENSG00000106638	7	72983262	72993121	5	1	1118599	6.11	5E-10	TBL2
Type 2 diabetes	gout	ENSG00000009950	7	73007524	73038873	24	2	1118599	7.04	9.4541E-13	MLXIPL
Type 2 diabetes	gout	ENSG00000119912	10	94211441	94333833	1	1	1118599	5.52	1.6524E-08	IDE
Type 2 diabetes	gout	ENSG00000148737	10	114710009	114927437	9	2	1118599	5.97	1.1827E-09	TCF7L2
Type 2 diabetes	gout	ENSG00000140718	16	53737875	54155853	66	1	1118599	5.78	3.6771E-09	FTO
Type 2 diabetes	gout	ENSG00000102908	16	69598997	69738569	17	1	1118599	6.45	5.6547E-11	NFAT5
Type 2 diabetes	gout	ENSG00000181019	16	69740899	69760854	1	1	1118599	6.19	3.0318E-10	NQO1
Type 2 diabetes	gout	ENSG00000198373	16	69796209	69975644	13	1	1118599	5.51	1.7813E-08	WWP2
Type 2 diabetes	gout	ENSG00000100344	22	44319619	44360368	63	1	1118599	6.25	2.0952E-10	PNPLA3
Type 2 diabetes	gout	ENSG00000100347	22	44351301	44406411	25	2	1118599	6.69	1.1495E-11	SAMM50

Type 2 diabetes	gout	ENSG00000188677	22	44395091	44568829	2	1	1118599	6.11	5E-10	PARVB
Type 2 diabetes	hypothyroidism/myxoedema	ENSG00000127603	1	39546988	39952849	17	1	1118599	5.8	3.2388E-09	MACF1
Type 2 diabetes	hypothyroidism/myxoedema	ENSG00000134215	1	108113782	108507766	80	2	1118599	6.27	1.77E-10	VAV3
Type 2 diabetes	hypothyroidism/myxoedema	ENSG00000081026	1	113933371	114228545	96	3	1118599	7.04	9.7183E-13	MAGI3
Type 2 diabetes	hypothyroidism/myxoedema	ENSG00000116793	1	114239453	114302111	44	2	1118599	6.11	5E-10	PHTF1
Type 2 diabetes	hypothyroidism/myxoedema	ENSG00000081019	1	114304454	114355098	20	2	1118599	7.78	3.6082E-15	RSBN1
Type 2 diabetes	hypothyroidism/myxoedema	ENSG00000134242	1	114356433	114414381	28	2	1118599	6.11	5E-10	PTPN22
Type 2 diabetes	hypothyroidism/myxoedema	ENSG00000188761	1	114420790	114430169	6	2	1118599	6.69	1.1121E-11	BCL2L15
Type 2 diabetes	hypothyroidism/myxoedema	ENSG00000134262	1	114437370	114447823	5	1	1118599	6.11	5E-10	AP4B1
Type 2 diabetes	hypothyroidism/myxoedema	ENSG00000118655	1	114447763	114456708	5	1	1118599	6.94	2.0206E-12	DCLRE1B

Type 2 diabetes	hypothyroidism/myxoedema	ENSG00000163349	1	114471814	114520426	2	1	1118599	5.42	2.9363E-08	HIPK1
Type 2 diabetes	hypothyroidism/myxoedema	ENSG00000115970	2	43393800	43823185	294	4	1118599	7.12	5.2674E-13	THADA
Type 2 diabetes	hypothyroidism/myxoedema	ENSG00000152518	2	43449541	43453748	1	1	1118599	6.89	2.8389E-12	ZFP36L2
Type 2 diabetes	hypothyroidism/myxoedema	ENSG00000123636	2	160175490	160473203	13	1	1118599	4.76	9.5445E-07	BAZ2B
Type 2 diabetes	hypothyroidism/myxoedema	ENSG00000136536	2	160569000	160627538	7	1	1118599	3.91	0.000045454	MARCH7
Type 2 diabetes	hypothyroidism/myxoedema	ENSG00000241399	2	160625364	160654753	14	1	1118599	4.87	5.5292E-07	CD302
Type 2 diabetes	hypothyroidism/myxoedema	ENSG00000248672	2	160628362	160761221	19	1	1118599	5.09	1.7623E-07	LY75-CD302
Type 2 diabetes	hypothyroidism/myxoedema	ENSG00000054219	2	160628362	160761260	19	1	1118599	5.09	1.7623E-07	LY75
Type 2 diabetes	hypothyroidism/myxoedema	ENSG00000138378	2	191894302	192016322	9	1	1118599	5.4	3.3655E-08	STAT4
Type 2 diabetes	hypothyroidism/myxoedema	ENSG00000157150	3	12194551	12200851	1	1	1118599	7.37	8.4125E-14	TIMP4

Type 2 diabetes	hypothyroidism/myxoedema	ENSG00000073792	3	185361527	185542844	68	2	1118599	6.11	5E-10	IGF2BP2
Type 2 diabetes	hypothyroidism/myxoedema	ENSG00000145012	3	187871072	188608460	1	1	1118599	5.82	2.9535E-09	LPP
Type 2 diabetes	hypothyroidism/myxoedema	ENSG00000109501	4	6271576	6304992	72	2	1118599	6.14	4.1877E-10	WFS1
Type 2 diabetes	hypothyroidism/myxoedema	ENSG00000113231	5	76506274	76725632	24	2	1118599	6.19	3.0253E-10	PDE8B
Type 2 diabetes	hypothyroidism/myxoedema	ENSG00000145996	6	20534688	21232635	13	3	1118599	7.05	8.7436E-13	CDKAL1
Type 2 diabetes	hypothyroidism/myxoedema	ENSG00000204542	6	31079000	31080336	7	2	1118599	7.31	1.3306E-13	C6orf15
Type 2 diabetes	hypothyroidism/myxoedema	ENSG00000204540	6	31082527	31107869	37	7	1118599	8.13	2.2204E-16	PSORS1C1
Type 2 diabetes	hypothyroidism/myxoedema	ENSG00000204539	6	31082867	31088223	4	1	1118599	5.97	1.221E-09	CDSN
Type 2 diabetes	hypothyroidism/myxoedema	ENSG00000204538	6	31105313	31107127	6	3	1118599	7.96	8.8818E-16	PSORS1C2
Type 2 diabetes	hypothyroidism/myxoedema	ENSG00000204536	6	31110216	31126015	2	1	1118599	5.76	4.2304E-09	CCHCR1

Type 2 diabetes	hypothyroidism/myxoedema	ENSG00000137310	6	31126319	31134936	2	1	1118599	5.81	3.0877E-09	TCF19
Type 2 diabetes	hypothyroidism/myxoedema	ENSG00000204531	6	31132119	31148508	3	1	1118599	5.83	2.8478E-09	POU5F1
Type 2 diabetes	hypothyroidism/myxoedema	ENSG00000234745	6	31321649	31324965	1	1	1118599	9.03	8.8364E-20	HLA-B
Type 2 diabetes	hypothyroidism/myxoedema	ENSG00000204516	6	31462658	31478901	56	5	1118599	7	1.2494E-12	MICB
Type 2 diabetes	hypothyroidism/myxoedema	ENSG00000198563	6	31497996	31510225	2	1	1118599	6.11	5E-10	DDX39B
Type 2 diabetes	hypothyroidism/myxoedema	ENSG00000254870	6	31497996	31514385	5	2	1118599	6.7	1.0182E-11	ATP6V1G2-DDX39B
Type 2 diabetes	hypothyroidism/myxoedema	ENSG00000213760	6	31512239	31516204	3	1	1118599	6.19	2.9484E-10	ATP6V1G2
Type 2 diabetes	hypothyroidism/myxoedema	ENSG00000204498	6	31514647	31526606	7	2	1118599	7.35	1.0175E-13	NFKBIL1
Type 2 diabetes	hypothyroidism/myxoedema	ENSG00000226979	6	31539831	31542101	5	1	1118599	6.11	5E-10	LTA
Type 2 diabetes	hypothyroidism/myxoedema	ENSG00000204482	6	31553901	31556686	2	1	1118599	6.11	5E-10	LST1

Type 2 diabetes	hypothyroidism/myxoedema	ENSG00000213719	6	31698358	31707540	2	1	1118599	8.18	1.4384E-16	CLIC1
Type 2 diabetes	hypothyroidism/myxoedema	ENSG00000204410	6	31707725	31732622	14	2	1118599	6.11	5E-10	MSH5
Type 2 diabetes	hypothyroidism/myxoedema	ENSG00000255152	6	31707797	31732628	14	2	1118599	6.11	5E-10	MSH5-SAPCD1
Type 2 diabetes	hypothyroidism/myxoedema	ENSG00000204390	6	31777396	31783437	2	1	1118599	6.11	5E-10	HSPA1L
Type 2 diabetes	hypothyroidism/myxoedema	ENSG00000204389	6	31783291	31785723	1	1	1118599	5.46	2.3632E-08	HSPA1A
Type 2 diabetes	hypothyroidism/myxoedema	ENSG00000204386	6	31825436	31830683	1	1	1118599	6.65	1.4894E-11	NEU1
Type 2 diabetes	hypothyroidism/myxoedema	ENSG00000204385	6	31830969	31846823	11	3	1118599	7.89	1.5543E-15	SLC44A4
Type 2 diabetes	hypothyroidism/myxoedema	ENSG00000204371	6	31847536	31865464	2	1	1118599	6.11	5E-10	EHMT2
Type 2 diabetes	hypothyroidism/myxoedema	ENSG00000166278	6	31865562	31913449	3	1	1118599	6.27	1.753E-10	C2
Type 2 diabetes	hypothyroidism/myxoedema	ENSG00000244255	6	31895475	31919825	3	2	1118599	6.43	6.5779E-11	CFB

Type 2 diabetes	hypothyroidism/myxoedema	ENSG00000243649	6	31895475	31919861	3	2	1118599	6.43	6.5779E-11	CFB
Type 2 diabetes	hypothyroidism/myxoedema	ENSG00000204344	6	31938868	31950598	4	2	1118599	6.46	5.1486E-11	STK19
Type 2 diabetes	hypothyroidism/myxoedema	ENSG00000168477	6	32008931	32083111	30	5	1118599	6.11	5E-10	TNXB
Type 2 diabetes	hypothyroidism/myxoedema	ENSG00000213676	6	32065953	32096030	6	2	1118599	7.55	2.1483E-14	ATF6B
Type 2 diabetes	hypothyroidism/myxoedema	ENSG00000196735	6	32595956	32614839	1	1	1118599	14.9	1E-50	HLA-DQA1
Type 2 diabetes	hypothyroidism/myxoedema	ENSG00000179344	6	32627244	32636160	1	1	1118599	14.9	1E-50	HLA-DQB1
Type 2 diabetes	hypothyroidism/myxoedema	ENSG00000241106	6	32780540	32784825	1	1	1118599	5.62	9.2947E-09	HLA-DOB
Type 2 diabetes	hypothyroidism/myxoedema	ENSG00000250264	6	32781544	32806599	20	4	1118599	6.11	5E-10	TAP2
Type 2 diabetes	hypothyroidism/myxoedema	ENSG00000204267	6	32789610	32806557	17	4	1118599	7.83	2.4425E-15	TAP2
Type 2 diabetes	hypothyroidism/myxoedema	ENSG00000204264	6	32808494	32812480	4	1	1118599	6.11	5E-10	PSMB8

Type 2 diabetes	hypothyroidism/myxoedema	ENSG00000240065	6	32811913	32827362	6	2	1118599	7.32	1.1963E-13	PSMB9
Type 2 diabetes	hypothyroidism/myxoedema	ENSG00000168394	6	32812986	32821755	4	1	1118599	6.11	5E-10	TAP1
Type 2 diabetes	hypothyroidism/myxoedema	ENSG00000112511	6	33378176	33384230	1	1	1118599	5.74	4.6977E-09	PHF1
Type 2 diabetes	hypothyroidism/myxoedema	ENSG00000112514	6	33384219	33386094	1	1	1118599	6.54	2.9945E-11	CUTA
Type 2 diabetes	hypothyroidism/myxoedema	ENSG00000197283	6	33387847	33421466	12	1	1118599	5.8	3.3161E-09	SYNGAP1
Type 2 diabetes	hypothyroidism/myxoedema	ENSG00000213588	6	33422356	33425325	1	1	1118599	5.58	1.1759E-08	ZBTB9
Type 2 diabetes	hypothyroidism/myxoedema	ENSG00000112182	6	90636248	91006627	17	1	1118599	5.71	5.7373E-09	BACH2
Type 2 diabetes	hypothyroidism/myxoedema	ENSG00000118520	6	131894284	131905472	2	1	1118599	5.22	9.0012E-08	ARG1
Type 2 diabetes	hypothyroidism/myxoedema	ENSG00000112282	6	131895106	131949369	10	1	1118599	5.73	5.0682E-09	MED23
Type 2 diabetes	hypothyroidism/myxoedema	ENSG00000154269	6	131949582	132068553	2	1	1118599	5.66	7.4987E-09	ENPP3

Type 2 diabetes	hypothyroidism/myxoedema	ENSG00000213066	6	167412670	167466201	50	2	1118599	6.46	5.2791E-11	FGFR1OP
Type 2 diabetes	hypothyroidism/myxoedema	ENSG00000136267	7	14184674	15014402	1	1	1118599	5.4	3.333E-08	DGKB
Type 2 diabetes	hypothyroidism/myxoedema	ENSG00000146830	7	100277130	100287071	2	1	1118599	5.64	8.5466E-09	GIGYF1
Type 2 diabetes	hypothyroidism/myxoedema	ENSG00000130427	7	100318423	100321323	1	1	1118599	6.13	4.5031E-10	EPO
Type 2 diabetes	hypothyroidism/myxoedema	ENSG00000182319	8	8175258	8244008	5	1	1118599	6.18	3.221E-10	SGK223
Type 2 diabetes	hypothyroidism/myxoedema	ENSG00000253958	8	8559448	8561616	1	1	1118599	5.35	4.4854E-08	CLDN23
Type 2 diabetes	hypothyroidism/myxoedema	ENSG00000147324	8	8640864	8751155	67	2	1118599	6.57	2.4593E-11	MFHAS1
Type 2 diabetes	hypothyroidism/myxoedema	ENSG00000104626	8	8859657	8974256	48	2	1118599	6.39	8.1854E-11	ERI1
Type 2 diabetes	hypothyroidism/myxoedema	ENSG00000173273	8	9413424	9639856	36	1	1118599	5.53	1.6459E-08	TNKS
Type 2 diabetes	hypothyroidism/myxoedema	ENSG00000175806	8	9911778	10286401	40	3	1118599	6.67	1.32E-11	MSRA

Type 2 diabetes	hypothyroidism/myxoedema	ENSG00000183638	8	10463859	10569697	13	1	1118599	5.41	3.182E-08	RP1L1
Type 2 diabetes	hypothyroidism/myxoedema	ENSG00000171056	8	10581278	10697357	30	1	1118599	5.77	3.9468E-09	SOX7
Type 2 diabetes	hypothyroidism/myxoedema	ENSG00000258724	8	10582909	10697357	30	1	1118599	5.77	3.9468E-09	SOX7
Type 2 diabetes	hypothyroidism/myxoedema	ENSG00000254093	8	10622473	10697394	19	1	1118599	5.76	4.1174E-09	PINX1
Type 2 diabetes	hypothyroidism/myxoedema	ENSG00000171044	8	10753555	11058875	150	4	1118599	6.86	3.5653E-12	XKR6
Type 2 diabetes	hypothyroidism/myxoedema	ENSG00000215346	8	10983980	10987745	2	1	1118599	5.99	1.0713E-09	AF131215.5
Type 2 diabetes	hypothyroidism/myxoedema	ENSG00000104643	8	11141925	11185646	28	2	1118599	6.13	4.3341E-10	MTMR9
Type 2 diabetes	hypothyroidism/myxoedema	ENSG00000184608	8	11225911	11296167	83	2	1118599	6.89	2.8644E-12	C8orf12
Type 2 diabetes	hypothyroidism/myxoedema	ENSG00000154319	8	11278972	11332224	2	1	1118599	5.25	7.5124E-08	FAM167A
Type 2 diabetes	hypothyroidism/myxoedema	ENSG00000136573	8	11351510	11422113	35	2	1118599	7.24	2.2787E-13	BLK

Type 2 diabetes	hypothyroidism/myxoedema	ENSG00000136574	8	11534468	11617511	7	1	1118599	5.41	3.111E-08	GATA4
Type 2 diabetes	hypothyroidism/myxoedema	ENSG00000205884	8	11831446	11832108	2	1	1118599	6.9	2.6817E-12	DEFB136
Type 2 diabetes	hypothyroidism/myxoedema	ENSG00000205882	8	11850692	11853821	3	1	1118599	5.63	8.7946E-09	DEFB134
Type 2 diabetes	hypothyroidism/myxoedema	ENSG00000255098	8	11870545	11873043	1	1	1118599	5.73	4.9824E-09	RP11-481A20.11
Type 2 diabetes	hypothyroidism/myxoedema	ENSG00000107249	9	3824127	4348392	9	1	1118599	5.99	1.0299E-09	GLIS3
Type 2 diabetes	hypothyroidism/myxoedema	ENSG00000178919	9	100615536	100618986	4	1	1118599	4.45	0.0000043	FOXE1
Type 2 diabetes	hypothyroidism/myxoedema	ENSG00000108175	10	80828792	81076276	2	1	1118599	5.11	1.6194E-07	ZMIZ1
Type 2 diabetes	hypothyroidism/myxoedema	ENSG00000148737	10	114710009	114927437	62	3	1118599	7.14	4.618E-13	TCF7L2
Type 2 diabetes	hypothyroidism/myxoedema	ENSG00000107679	10	124134212	124191867	40	2	1118599	5.46	2.4253E-08	PLEKHA1
Type 2 diabetes	hypothyroidism/myxoedema	ENSG00000149187	11	47487496	47587121	1	1	1118599	5.49	2.0055E-08	CELF1

Type 2 diabetes	hypothyroidism/myxoedema	ENSG00000124920	11	61520114	61555990	5	1	1118599	4.61	2.0539E-06	MYRF
Type 2 diabetes	hypothyroidism/myxoedema	ENSG00000134825	11	61535973	61560274	8	1	1118599	5.13	1.4133E-07	TMEM258
Type 2 diabetes	hypothyroidism/myxoedema	ENSG00000168496	11	61560109	61564716	1	1	1118599	5.82	2.943E-09	FEN1
Type 2 diabetes	hypothyroidism/myxoedema	ENSG00000134824	11	61560452	61634826	11	1	1118599	5.74	4.8367E-09	FADS2
Type 2 diabetes	hypothyroidism/myxoedema	ENSG00000149485	11	61567099	61596790	4	1	1118599	5.71	5.7398E-09	FADS1
Type 2 diabetes	hypothyroidism/myxoedema	ENSG00000186635	11	72396114	72504644	17	1	1118599	6.79	5.4557E-12	ARAP1
Type 2 diabetes	hypothyroidism/myxoedema	ENSG00000214530	11	72465774	72504726	5	1	1118599	5.97	1.1555E-09	STARD10
Type 2 diabetes	hypothyroidism/myxoedema	ENSG00000137478	11	72547790	72853306	11	1	1118599	5.28	6.5492E-08	FCHSD2
Type 2 diabetes	hypothyroidism/myxoedema	ENSG00000134640	11	92702886	92718232	1	1	1118599	6.1	5.2249E-10	MTNR1B
Type 2 diabetes	hypothyroidism/myxoedema	ENSG00000069493	12	9817565	9848413	12	2	1118599	5.18	1.0874E-07	CLEC2D

Type 2 diabetes	hypothyroidism/myxoedema	ENSG00000184293	12	9868456	9885895	29	2	1118599	5.46	2.4238E-08	CLECL1
Type 2 diabetes	hypothyroidism/myxoedema	ENSG00000110848	12	9905082	9913497	2	1	1118599	5.93	1.4797E-09	CD69
Type 2 diabetes	hypothyroidism/myxoedema	ENSG00000179088	12	103631369	103889749	9	1	1118599	5.61	1.0203E-08	C12orf42
Type 2 diabetes	hypothyroidism/myxoedema	ENSG00000111249	12	111471828	111788358	19	1	1118599	6.3	1.533E-10	CUX2
Type 2 diabetes	hypothyroidism/myxoedema	ENSG00000111252	12	111843752	111889427	2	1	1118599	6.11	5E-10	SH2B3
Type 2 diabetes	hypothyroidism/myxoedema	ENSG00000204842	12	111890018	112037480	8	2	1118599	7.05	8.6831E-13	ATXN2
Type 2 diabetes	hypothyroidism/myxoedema	ENSG00000089234	12	112079950	112123790	1	1	1118599	8.91	2.6301E-19	BRAP
Type 2 diabetes	hypothyroidism/myxoedema	ENSG00000111271	12	112123857	112194903	2	1	1118599	6.11	5E-10	ACAD10
Type 2 diabetes	hypothyroidism/myxoedema	ENSG00000111300	12	112464500	112546826	1	1	1118599	8.81	6.4211E-19	NAA25
Type 2 diabetes	hypothyroidism/myxoedema	ENSG00000173064	12	112597992	112819896	1	1	1118599	8.64	2.7955E-18	HECTD4

Type 2 diabetes	hypothyroidism/myxoedema	ENSG00000179295	12	112856155	112947717	2	1	1118599	6.11	5E-10	PTPN11
Type 2 diabetes	hypothyroidism/myxoedema	ENSG00000157837	12	121200313	121342174	6	1	1118599	5.63	8.9151E-09	SPPL3
Type 2 diabetes	hypothyroidism/myxoedema	ENSG00000135100	12	121416346	121440315	1	1	1118599	5.59	1.1085E-08	HNF1A
Type 2 diabetes	hypothyroidism/myxoedema	ENSG00000135114	12	121458095	121477045	4	1	1118599	5.56	1.325E-08	OASL
Type 2 diabetes	hypothyroidism/myxoedema	ENSG00000172575	15	38780304	38857776	19	1	1118599	6.45	5.674E-11	RASGRP1
Type 2 diabetes	hypothyroidism/myxoedema	ENSG00000108771	17	40253422	40264751	5	1	1118599	5.9	1.8386E-09	DHX58
Type 2 diabetes	hypothyroidism/myxoedema	ENSG00000267261	17	40271692	40306934	6	1	1118599	5.85	2.4944E-09	CTD-2132N18.3
Type 2 diabetes	hypothyroidism/myxoedema	ENSG00000108774	17	40276994	40307035	6	1	1118599	5.85	2.4944E-09	RAB5C
Type 2 diabetes	hypothyroidism/myxoedema	ENSG00000168610	17	40465342	40540586	26	1	1118599	5.99	1.0631E-09	STAT3
Type 2 diabetes	Hypertension	ENSG00000115970	2	43393800	43823185	1	1	1016860	5.36	4.2788E-08	THADA

Type 2 diabetes	Hypertension	ENSG00000073792	3	185361527	185542844	29	1	1016860	5.48	2.172E-08	IGF2BP2
Type 2 diabetes	Hypertension	ENSG00000109501	4	6271576	6304992	8	1	1016860	6.03	8.02E-10	WFS1
Type 2 diabetes	Hypertension	ENSG00000145996	6	20534688	21232635	73	3	1016860	7.03	1.0477E-12	CDKAL1
Type 2 diabetes	Hypertension	ENSG00000148737	10	114710009	114927437	70	3	1016860	7.45	4.5353E-14	TCF7L2
hypoglycemia	hypothyroidism/myxoedema	ENSG00000162631	1	107682629	108026080	2	1	625134	6.17	3.3505E-10	NTNG1
hypoglycemia	hypothyroidism/myxoedema	ENSG00000198130	2	191054461	191208919	1	1	625134	5.64	8.6841E-09	HIBCH
hypoglycemia	hypothyroidism/myxoedema	ENSG00000138442	2	203739505	203879521	1	1	625134	8.87	3.7826E-19	WDR12
hypoglycemia	hypothyroidism/myxoedema	ENSG00000144426	2	203879602	204091101	1	1	625134	9.63	2.8659E-22	NBEAL1
hypoglycemia	hypothyroidism/myxoedema	ENSG00000079689	6	25652464	25702011	1	1	625134	5.62	9.4251E-09	SCGN
hypoglycemia	hypothyroidism/myxoedema	ENSG00000112763	6	26458150	26476849	1	1	625134	5.97	1.2097E-09	BTN2A1

hypoglycemia	hypothyroidism/myxoedema	ENSG00000233822	6	27806323	27823487	1	1	625134	7.87	1.7088E-15	HIST1H2BN
hypoglycemia	hypothyroidism/myxoedema	ENSG00000235109	6	28292470	28324048	1	1	625134	8.53	7.0694E-18	ZSCAN31
hypoglycemia	hypothyroidism/myxoedema	ENSG00000204681	6	29523406	29601753	2	1	625134	5.26	7.0697E-08	GABBR1
hypoglycemia	hypothyroidism/myxoedema	ENSG00000204614	6	30103885	30116512	3	2	625134	6.19	2.9287E-10	TRIM40
hypoglycemia	hypothyroidism/myxoedema	ENSG00000261272	6	30978251	31003179	1	1	625134	7.5	3.1402E-14	MUC22
hypoglycemia	hypothyroidism/myxoedema	ENSG00000204540	6	31082527	31107869	2	1	625134	6.11	5E-10	PSORS1C1
hypoglycemia	hypothyroidism/myxoedema	ENSG00000204536	6	31110216	31126015	4	2	625134	6.89	2.7809E-12	CCHCR1
hypoglycemia	hypothyroidism/myxoedema	ENSG00000204531	6	31132119	31148508	1	1	625134	11.3	9.2833E-30	POU5F1
hypoglycemia	hypothyroidism/myxoedema	ENSG00000206344	6	31165537	31171745	1	1	625134	9.91	1.9689E-23	HCG27
hypoglycemia	hypothyroidism/myxoedema	ENSG00000204525	6	31236526	31239907	4	2	625134	7.09	6.5509E-13	HLA-C

hypoglycemia	hypothyroidism/myxoedema	ENSG00000234745	6	31321649	31324965	1	1	625134	8.25	7.9989E-17	HLA-B	
hypoglycemia	hypothyroidism/myxoedema	ENSG00000204520	6	31371356	31383092	4	2	625134	6.75	7.5948E-12	MICA	
hypoglycemia	hypothyroidism/myxoedema	ENSG00000198563	6	31497996	31510225	4	2	625134	6.84	4.0051E-12	DDX39B	
hypoglycemia	hypothyroidism/myxoedema	ENSG00000254870	6	31497996	31514385	4	2	625134	6.84	4.0051E-12	ATP6V1G2-DDX39B	
hypoglycemia	hypothyroidism/myxoedema	ENSG00000204498	6	31514647	31526606	2	1	625134	6.11	5E-10	NFKBIL1	
hypoglycemia	hypothyroidism/myxoedema	ENSG00000204428	6	31644461	31651817	1	1	625134	12.8	4.6437E-38	LY6G5C	
hypoglycemia	hypothyroidism/myxoedema	ENSG00000204390	6	31777396	31783437	1	1	625134	14.9	1E-50	HSPA1L	
hypoglycemia	hypothyroidism/myxoedema	ENSG00000166278	6	31865562	31913449	1	1	625134	5.85	2.4346E-09	C2	
hypoglycemia	hypothyroidism/myxoedema	ENSG00000204366	6	31867384	31869769	1	1	625134	5.85	2.4346E-09	ZBTB12	
hypoglycemia	hypothyroidism/myxoedema	ENSG00000213676	6	32065953	32096030	1	1	625134	14.9	1E-50	ATF6B	

hypoglycemia	hypothyroidism/myxoedema	ENSG00000221988	6	32121218	32134011	1	1	625134	13.2	2.5168E-40	PPT2
hypoglycemia	hypothyroidism/myxoedema	ENSG00000258388	6	32121622	32139755	1	1	625134	13.2	2.5168E-40	PPT2-EGFL8
hypoglycemia	hypothyroidism/myxoedema	ENSG00000204296	6	32256303	32339684	4	2	625134	6.92	2.1881E-12	C6orf10
hypoglycemia	hypothyroidism/myxoedema	ENSG00000204290	6	32361740	32374905	7	2	625134	6.11	5E-10	BTNL2
hypoglycemia	hypothyroidism/myxoedema	ENSG00000204287	6	32407619	32412823	1	1	625134	14.9	1E-50	HLA-DRA
hypoglycemia	hypothyroidism/myxoedema	ENSG00000196735	6	32595956	32614839	22	6	625134	6.11	5E-10	HLA-DQA1
hypoglycemia	hypothyroidism/myxoedema	ENSG00000179344	6	32627244	32636160	9	4	625134	7.94	9.992E-16	HLA-DQB1
hypoglycemia	hypothyroidism/myxoedema	ENSG00000237541	6	32709119	32714992	1	1	625134	8.57	5.4044E-18	HLA-DQA2
hypoglycemia	hypothyroidism/myxoedema	ENSG00000241106	6	32780540	32784825	3	2	625134	6.44	5.9134E-11	HLA-DOB
hypoglycemia	hypothyroidism/myxoedema	ENSG00000250264	6	32781544	32806599	5	3	625134	7.68	8.1046E-15	TAP2

hypoglycemia	hypothyroidism/myxoedema	ENSG00000204267	6	32789610	32806557	1	1	625134	11.1	9.1028E-29	TAP2
hypoglycemia	hypothyroidism/myxoedema	ENSG00000240065	6	32811913	32827362	1	1	625134	14.9	1E-50	PSMB9
hypoglycemia	hypothyroidism/myxoedema	ENSG00000204257	6	32916390	32936871	1	1	625134	7.3	1.3986E-13	HLA-DMA
hypoglycemia	hypothyroidism/myxoedema	ENSG00000231389	6	33032346	33048552	3	1	625134	5.4	3.2512E-08	HLA-DPA1
hypoglycemia	hypothyroidism/myxoedema	ENSG00000223865	6	33043703	33054978	1	1	625134	8.24	8.8939E-17	HLA-DPB1
hypoglycemia	hypothyroidism/myxoedema	ENSG00000204248	6	33130458	33160276	1	1	625134	8.4	2.2279E-17	COL11A2
hypoglycemia	hypothyroidism/myxoedema	ENSG00000096433	6	33588142	33664351	2	1	625134	6.11	5E-10	ITPR3
hypoglycemia	hypothyroidism/myxoedema	ENSG00000135299	6	90142889	90343553	1	1	625134	5.88	2.0035E-09	ANKRD6
hypoglycemia	hypothyroidism/myxoedema	ENSG00000071242	6	166822852	167319939	2	1	625134	6.11	5E-10	RPS6KA2
hypoglycemia	hypothyroidism/myxoedema	ENSG00000026508	11	35160417	35253949	1	1	625134	6.15	3.9191E-10	CD44

gout	hypothyroidism/myxoedema	ENSG00000134215	1	108113782	108507766	24	1	925866	6.05	7.2569E-10	VAV3
gout	hypothyroidism/myxoedema	ENSG00000138378	2	191894302	192016322	5	1	925866	4.87	5.5888E-07	STAT4
gout	hypothyroidism/myxoedema	ENSG00000109667	4	9772777	10056560	105	4	925866	6.11	5E-10	SLC2A9
gout	hypothyroidism/myxoedema	ENSG00000071127	4	10075963	10118573	7	2	925866	7.53	2.4647E-14	WDR1
gout	hypothyroidism/myxoedema	ENSG00000118762	4	88928820	88998929	27	3	925866	7.63	1.2101E-14	PKD2
gout	hypothyroidism/myxoedema	ENSG00000118777	4	89011416	89152474	35	6	925866	6.11	5E-10	ABCG2
gout	hypothyroidism/myxoedema	ENSG00000079691	6	25279306	25620758	13	2	925866	7.08	7.162E-13	LRRC16A
gout	hypothyroidism/myxoedema	ENSG00000079689	6	25652464	25702011	2	1	925866	6.11	5E-10	SCGN
gout	hypothyroidism/myxoedema	ENSG00000146039	6	25754927	25781419	13	1	925866	7.37	8.7652E-14	SLC17A4
gout	hypothyroidism/myxoedema	ENSG00000124568	6	25783125	25832287	37	3	925866	6.11	5E-10	SLC17A1

gout	hypothyroidism/myxoedema	ENSG00000124564	6	25833294	25882514	42	3	925866	6.59	2.1543E-11	SLC17A3
gout	hypothyroidism/myxoedema	ENSG00000112337	6	25912982	25930946	11	1	925866	6.11	5E-10	SLC17A2
gout	hypothyroidism/myxoedema	ENSG00000112343	6	25963030	25987384	9	1	925866	7.22	2.6551E-13	TRIM38
gout	hypothyroidism/myxoedema	ENSG00000124610	6	26017260	26018040	1	1	925866	7.1	6.2352E-13	HIST1H1A
gout	hypothyroidism/myxoedema	ENSG00000196176	6	26021907	26022278	1	1	925866	7.09	6.4913E-13	HIST1H4A
gout	hypothyroidism/myxoedema	ENSG00000196532	6	26045639	26046097	1	1	925866	7.12	5.2393E-13	HIST1H3C
gout	hypothyroidism/myxoedema	ENSG00000010704	6	26087509	26098571	4	1	925866	6.11	5E-10	HFE
gout	hypothyroidism/myxoedema	ENSG00000180596	6	26115101	26124154	4	1	925866	5.47	0.000000022	HIST1H2BC
gout	hypothyroidism/myxoedema	ENSG00000180573	6	26124373	26139344	4	2	925866	6.41	7.2923E-11	HIST1H2AC
gout	hypothyroidism/myxoedema	ENSG00000158373	6	26158349	26171577	1	1	925866	6.04	7.5391E-10	HIST1H2BD

gout	hypothyroidism/myxoedema	ENSG00000197846	6	26199748	26200942	1	1	925866	7.94	9.9765E-16	HIST1H2BF
gout	hypothyroidism/myxoedema	ENSG00000186470	6	26365387	26378546	48	1	925866	5.05	2.2483E-07	BTN3A2
gout	hypothyroidism/myxoedema	ENSG00000124508	6	26383324	26395102	3	1	925866	5.29	6.0154E-08	BTN2A2
gout	hypothyroidism/myxoedema	ENSG00000026950	6	26402465	26415444	5	1	925866	6.17	3.3564E-10	BTN3A1
gout	hypothyroidism/myxoedema	ENSG00000111801	6	26440700	26453643	3	1	925866	5.56	1.3237E-08	BTN3A3
gout	hypothyroidism/myxoedema	ENSG00000112763	6	26458150	26476849	19	2	925866	7.09	6.5625E-13	BTN2A1
gout	hypothyroidism/myxoedema	ENSG00000124557	6	26501449	26510650	2	1	925866	6.11	5E-10	BTN1A1
gout	hypothyroidism/myxoedema	ENSG00000182952	6	26538633	26546482	1	1	925866	6.36	9.894E-11	HMGN4
gout	hypothyroidism/myxoedema	ENSG00000146109	6	26597180	26600278	2	1	925866	6.11	5E-10	ABT1
gout	hypothyroidism/myxoedema	ENSG00000181315	6	26636518	26659980	1	1	925866	7.41	6.274E-14	ZNF322

gout	hypothyroidism/myxoedema	ENSG00000112812	6	27215480	27224403	1	1	925866	5.46	2.366E-08	PRSS16
gout	hypothyroidism/myxoedema	ENSG00000158553	6	27253682	27279949	6	1	925866	4.87	5.5746E-07	POM121L2
gout	hypothyroidism/myxoedema	ENSG00000124613	6	27342394	27371683	4	1	925866	6.11	5E-10	ZNF391
gout	hypothyroidism/myxoedema	ENSG00000096654	6	27418522	27440897	21	2	925866	6.82	4.6967E-12	ZNF184
gout	hypothyroidism/myxoedema	ENSG00000185130	6	27775257	27775709	2	1	925866	5.4	3.3241E-08	HIST1H2BL
gout	hypothyroidism/myxoedema	ENSG00000182611	6	27782112	27782607	2	1	925866	7.23	2.3913E-13	HIST1H2AJ
gout	hypothyroidism/myxoedema	ENSG00000233822	6	27806323	27823487	13	1	925866	7.74	4.9405E-15	HIST1H2BN
gout	hypothyroidism/myxoedema	ENSG00000198374	6	27833034	27833606	1	1	925866	7.47	3.8844E-14	HIST1H2AL
gout	hypothyroidism/myxoedema	ENSG00000184357	6	27834570	27835359	2	1	925866	6.11	5E-10	HIST1H1B
gout	hypothyroidism/myxoedema	ENSG00000182572	6	27839623	27840099	1	1	925866	6.86	3.3821E-12	HIST1H3I

gout	hypothyroidism/myxoedema	ENSG00000198558	6	27840926	27841289	1	1	925866	7.7	6.8146E-15	HIST1H4L
gout	hypothyroidism/myxoedema	ENSG00000168131	6	27878963	27880174	2	1	925866	7.91	1.3146E-15	OR2B2
gout	hypothyroidism/myxoedema	ENSG00000197279	6	28048753	28057341	1	1	925866	6.51	3.7161E-11	ZNF165
gout	hypothyroidism/myxoedema	ENSG00000196812	6	28092338	28097860	2	1	925866	6.55	2.8162E-11	ZSCAN16
gout	hypothyroidism/myxoedema	ENSG00000198315	6	28109688	28127250	4	1	925866	6.11	5E-10	ZKSCAN8
gout	hypothyroidism/myxoedema	ENSG00000137185	6	28192664	28201260	1	1	925866	7.89	1.5345E-15	ZSCAN9
gout	hypothyroidism/myxoedema	ENSG00000187626	6	28212401	28227011	10	2	925866	7.46	4.2466E-14	ZKSCAN4
gout	hypothyroidism/myxoedema	ENSG00000189134	6	28227098	28228736	1	1	925866	6.46	5.2667E-11	NKAPL
gout	hypothyroidism/myxoedema	ENSG00000137338	6	28249314	28270326	10	1	925866	6.11	5E-10	PGBD1
gout	hypothyroidism/myxoedema	ENSG00000235109	6	28292470	28324048	12	1	925866	6.11	5E-10	ZSCAN31

gout	hypothyroidism/myxoedema	ENSG00000189298	6	28317691	28336947	4	1	925866	7.77	4.0738E-15	ZKSCAN3
gout	hypothyroidism/myxoedema	ENSG00000158691	6	28346732	28367511	9	1	925866	7.77	4.0016E-15	ZSCAN12
gout	hypothyroidism/myxoedema	ENSG00000198704	6	28471073	28495992	1	1	925866	7.63	1.1494E-14	GPX6
gout	hypothyroidism/myxoedema	ENSG00000232040	6	28539407	28583989	1	1	925866	7.61	1.3703E-14	SCAND3
gout	hypothyroidism/myxoedema	ENSG00000204713	6	28870779	28891766	5	1	925866	7.5	3.1741E-14	TRIM27
gout	hypothyroidism/myxoedema	ENSG00000243729	6	29323007	29399744	12	2	925866	6.18	3.2193E-10	OR5V1
gout	hypothyroidism/myxoedema	ENSG00000112462	6	29341200	29343068	1	1	925866	7.19	3.2388E-13	OR12D3
gout	hypothyroidism/myxoedema	ENSG00000204694	6	29393281	29424848	3	1	925866	6.11	5E-10	OR11A1
gout	hypothyroidism/myxoedema	ENSG00000213886	6	29523292	29527702	2	1	925866	7.66	8.9557E-15	UBD
gout	hypothyroidism/myxoedema	ENSG00000204681	6	29523406	29601753	9	2	925866	7.96	8.3267E-16	GABBR1

gout	hypothyroidism/myxoedema	ENSG00000066379	6	30026676	30032686	1	1	925866	6.87	3.2281E-12	ZNRD1
gout	hypothyroidism/myxoedema	ENSG00000204618	6	30038043	30043664	1	1	925866	6.86	3.4535E-12	RNF39
gout	hypothyroidism/myxoedema	ENSG00000204616	6	30070674	30080883	11	1	925866	6.18	3.2141E-10	TRIM31
gout	hypothyroidism/myxoedema	ENSG00000204614	6	30103885	30116512	1	1	925866	7.33	1.1312E-13	TRIM40
gout	hypothyroidism/myxoedema	ENSG00000204613	6	30119722	30128711	4	1	925866	5.98	1.1296E-09	TRIM10
gout	hypothyroidism/myxoedema	ENSG00000204610	6	30130993	30140473	2	1	925866	5.34	4.721E-08	TRIM15
gout	hypothyroidism/myxoedema	ENSG00000234127	6	30152232	30181204	15	1	925866	6.77	6.4627E-12	TRIM26
gout	hypothyroidism/myxoedema	ENSG00000204599	6	30294256	30311506	4	1	925866	6.11	5E-10	TRIM39
gout	hypothyroidism/myxoedema	ENSG00000248167	6	30297359	30314631	4	1	925866	5.23	8.3773E-08	TRIM39-RPP21
gout	hypothyroidism/myxoedema	ENSG00000204590	6	30509154	30524951	1	1	925866	6.84	4.0832E-12	GNL1

gout	hypothyroidism/myxoedema	ENSG00000204574	6	30539153	30564956	1	1	925866	6.79	5.5983E-12	ABCF1
gout	hypothyroidism/myxoedema	ENSG00000137343	6	30594619	30614600	3	1	925866	6.11	5E-10	ATAT1
gout	hypothyroidism/myxoedema	ENSG00000204564	6	30614816	30620987	1	1	925866	6.82	4.6447E-12	C6orf136
gout	hypothyroidism/myxoedema	ENSG00000146112	6	30644166	30655672	2	1	925866	6.79	5.6557E-12	PPP1R18
gout	hypothyroidism/myxoedema	ENSG00000137404	6	30655824	30659197	1	1	925866	6.8	5.3064E-12	NRM
gout	hypothyroidism/myxoedema	ENSG00000196230	6	30687978	30693203	4	1	925866	6.47	4.7981E-11	TUBB
gout	hypothyroidism/myxoedema	ENSG00000137312	6	30695486	30710510	8	1	925866	6.11	5E-10	FLOT1
gout	hypothyroidism/myxoedema	ENSG00000204580	6	30844198	30867933	8	1	925866	6.08	5.9184E-10	DDR1
gout	hypothyroidism/myxoedema	ENSG00000213780	6	30875961	30881883	2	1	925866	6.11	5E-10	GTF2H4
gout	hypothyroidism/myxoedema	ENSG00000137411	6	30876019	30894236	3	1	925866	6.11	5E-10	VARS2

gout	hypothyroidism/myxoedema	ENSG00000196260	6	30899130	30899952	2	1	925866	6.46	5.1918E-11	SFTA2
gout	hypothyroidism/myxoedema	ENSG00000168631	6	30908749	30921998	5	1	925866	6.18	3.2263E-10	DPCR1
gout	hypothyroidism/myxoedema	ENSG00000204544	6	30951495	30957680	3	1	925866	6.11	5E-10	MUC21
gout	hypothyroidism/myxoedema	ENSG00000261272	6	30978251	31003179	8	2	925866	7.88	1.6098E-15	MUC22
gout	hypothyroidism/myxoedema	ENSG00000204542	6	31079000	31080336	8	2	925866	7.82	2.72E-15	C6orf15
gout	hypothyroidism/myxoedema	ENSG00000204540	6	31082527	31107869	104	8	925866	6.11	5E-10	PSORS1C1
gout	hypothyroidism/myxoedema	ENSG00000204539	6	31082867	31088223	32	3	925866	7.87	1.8319E-15	CDSN
gout	hypothyroidism/myxoedema	ENSG00000204538	6	31105313	31107127	2	1	925866	6.11	5E-10	PSORS1C2
gout	hypothyroidism/myxoedema	ENSG00000204536	6	31110216	31126015	39	3	925866	6.47	4.7451E-11	CCHCR1
gout	hypothyroidism/myxoedema	ENSG00000137310	6	31126319	31134936	20	3	925866	5.49	2.0426E-08	TCF19

gout	hypothyroidism/myxoedema	ENSG00000204531	6	31132119	31148508	23	3	925866	7.33	1.1846E-13	POU5F1
gout	hypothyroidism/myxoedema	ENSG00000204525	6	31236526	31239907	3	1	925866	6.05	7.3285E-10	HLA-C
gout	hypothyroidism/myxoedema	ENSG00000234745	6	31321649	31324965	4	1	925866	6.11	5E-10	HLA-B
gout	hypothyroidism/myxoedema	ENSG00000204520	6	31371356	31383092	2	1	925866	6.11	5E-10	MICA
gout	hypothyroidism/myxoedema	ENSG00000204516	6	31462658	31478901	41	5	925866	6.77	6.6561E-12	MICB
gout	hypothyroidism/myxoedema	ENSG00000204511	6	31496494	31498009	1	1	925866	7.36	8.9194E-14	MCCD1
gout	hypothyroidism/myxoedema	ENSG00000198563	6	31497996	31510225	8	2	925866	7.23	2.3836E-13	DDX39B
gout	hypothyroidism/myxoedema	ENSG00000254870	6	31497996	31514385	11	2	925866	7.68	7.8826E-15	ATP6V1G2-DDX39B
gout	hypothyroidism/myxoedema	ENSG00000213760	6	31512239	31516204	4	1	925866	6.43	6.595E-11	ATP6V1G2
gout	hypothyroidism/myxoedema	ENSG00000204498	6	31514647	31526606	7	2	925866	6.74	8.0217E-12	NFKBIL1

gout	hypothyroidism/myxoedema	ENSG00000226979	6	31539831	31542101	5	1	925866	6.78	5.8964E-12	LTA
gout	hypothyroidism/myxoedema	ENSG00000204482	6	31553901	31556686	2	1	925866	6.11	5E-10	LST1
gout	hypothyroidism/myxoedema	ENSG00000204475	6	31556672	31560762	1	1	925866	7.32	1.2427E-13	NCR3
gout	hypothyroidism/myxoedema	ENSG00000204469	6	31588497	31605548	3	2	925866	6.41	7.1404E-11	PRRC2A
gout	hypothyroidism/myxoedema	ENSG00000204463	6	31606805	31620482	3	1	925866	5.02	2.6004E-07	BAG6
gout	hypothyroidism/myxoedema	ENSG00000204444	6	31620193	31625987	2	1	925866	6.79	5.5269E-12	APOM
gout	hypothyroidism/myxoedema	ENSG00000204435	6	31633013	31638120	1	1	925866	6.84	3.9435E-12	CSNK2B
gout	hypothyroidism/myxoedema	ENSG00000263020	6	31633879	31641323	2	1	925866	6.11	5E-10	CSNK2B-LY6G5B-1181
gout	hypothyroidism/myxoedema	ENSG00000240053	6	31637944	31641553	2	1	925866	5.36	4.1381E-08	LY6G5B
gout	hypothyroidism/myxoedema	ENSG00000204428	6	31644461	31651817	1	1	925866	5.43	2.8285E-08	LY6G5C

gout	hypothyroidism/myxoedema	ENSG00000204427	6	31654726	31671221	1	1	925866	6.8	5.1384E-12	ABHD16A
gout	hypothyroidism/myxoedema	ENSG00000204422	6	31654739	31681849	3	1	925866	6.78	6.0215E-12	XXbac-BPG32J3.20
gout	hypothyroidism/myxoedema	ENSG00000204424	6	31674640	31685581	1	1	925866	6.76	6.8814E-12	LY6G6F
gout	hypothyroidism/myxoedema	ENSG00000250641	6	31674681	31685695	2	1	925866	6.11	5E-10	MEGT1
gout	hypothyroidism/myxoedema	ENSG00000213722	6	31694815	31698394	1	1	925866	6.4	7.6318E-11	DDAH2
gout	hypothyroidism/myxoedema	ENSG00000213719	6	31698358	31707540	2	1	925866	6.66	1.4164E-11	CLIC1
gout	hypothyroidism/myxoedema	ENSG00000204410	6	31707725	31732622	14	1	925866	5.45	2.5021E-08	MSH5
gout	hypothyroidism/myxoedema	ENSG00000255152	6	31707797	31732628	14	1	925866	5.45	2.5021E-08	MSH5-SAPCD1
gout	hypothyroidism/myxoedema	ENSG00000204396	6	31733367	31745108	3	1	925866	6.66	1.3616E-11	VWA7
gout	hypothyroidism/myxoedema	ENSG00000204394	6	31745295	31763730	4	1	925866	5.22	9.0832E-08	VARS

gout	hypothyroidism/myxoedema	ENSG00000204392	6	31765173	31774761	4	1	925866	6.11	5E-10	LSM2
gout	hypothyroidism/myxoedema	ENSG00000204390	6	31777396	31783437	1	1	925866	6.06	6.8801E-10	HSPA1L
gout	hypothyroidism/myxoedema	ENSG00000204387	6	31802385	31807541	2	1	925866	6.64	1.5604E-11	C6orf48
gout	hypothyroidism/myxoedema	ENSG00000204385	6	31830969	31846823	4	2	925866	6.11	5E-10	SLC44A4
gout	hypothyroidism/myxoedema	ENSG00000166278	6	31865562	31913449	5	1	925866	6.6	2.1007E-11	C2
gout	hypothyroidism/myxoedema	ENSG00000244255	6	31895475	31919825	2	1	925866	6.23	2.2754E-10	CFB
gout	hypothyroidism/myxoedema	ENSG00000243649	6	31895475	31919861	2	1	925866	6.23	2.2754E-10	CFB
gout	hypothyroidism/myxoedema	ENSG00000204344	6	31938868	31950598	1	1	925866	6.65	1.4812E-11	STK19
gout	hypothyroidism/myxoedema	ENSG00000168477	6	32008931	32083111	15	1	925866	5.19	1.0537E-07	TNXB
gout	hypothyroidism/myxoedema	ENSG00000213676	6	32065953	32096030	3	1	925866	6.11	5E-10	ATF6B

gout	hypothyroidism/myxoedema	ENSG00000221988	6	32121218	32134011	2	1	925866	6.11	5E-10	PPT2
gout	hypothyroidism/myxoedema	ENSG00000258388	6	32121622	32139755	2	1	925866	6.11	5E-10	PPT2-EGFL8
gout	hypothyroidism/myxoedema	ENSG00000204296	6	32256303	32339684	19	1	925866	5.97	1.1617E-09	C6orf10
gout	hypothyroidism/myxoedema	ENSG00000204290	6	32361740	32374905	1	1	925866	6.22	2.5631E-10	BTNL2
gout	hypothyroidism/myxoedema	ENSG00000204287	6	32407619	32412823	1	1	925866	6.25	2.0868E-10	HLA-DRA
gout	hypothyroidism/myxoedema	ENSG00000198502	6	32485120	32498064	5	2	925866	6.11	5E-10	HLA-DRB5
gout	hypothyroidism/myxoedema	ENSG00000196126	6	32546546	32557625	1	1	925866	5.5	1.9145E-08	HLA-DRB1
gout	hypothyroidism/myxoedema	ENSG00000196735	6	32595956	32614839	97	5	925866	6.11	5E-10	HLA-DQA1
gout	hypothyroidism/myxoedema	ENSG00000179344	6	32627244	32636160	19	4	925866	7.71	6.4393E-15	HLA-DQB1
gout	hypothyroidism/myxoedema	ENSG00000250264	6	32781544	32806599	1	1	925866	5.8	3.3573E-09	TAP2

gout	hypothyroidism/myxoedema	ENSG00000204267	6	32789610	32806557	1	1	925866	5.8	3.3573E-09	TAP2
gout	hypothyroidism/myxoedema	ENSG00000242574	6	32902406	32908847	1	1	925866	6.23	2.3387E-10	HLA-DMB
gout	hypothyroidism/myxoedema	ENSG00000231389	6	33032346	33048552	119	2	925866	5.7	5.8466E-09	HLA-DPA1
gout	hypothyroidism/myxoedema	ENSG00000223865	6	33043703	33054978	105	2	925866	6.85	3.772E-12	HLA-DPB1
gout	hypothyroidism/myxoedema	ENSG00000223501	6	33218049	33239824	7	1	925866	4.64	1.7388E-06	VPS52
gout	hypothyroidism/myxoedema	ENSG00000231500	6	33239787	33244287	5	1	925866	5.83	2.8321E-09	RPS18
gout	hypothyroidism/myxoedema	ENSG00000235863	6	33244917	33252609	2	1	925866	5.53	1.6068E-08	B3GALT4
gout	hypothyroidism/myxoedema	ENSG00000227057	6	33246885	33257304	4	1	925866	5.9	1.8491E-09	WDR46
gout	hypothyroidism/myxoedema	ENSG00000231925	6	33267471	33282164	2	1	925866	5.21	9.4908E-08	TAPBP
gout	hypothyroidism/myxoedema	ENSG00000236104	6	33282183	33285719	1	1	925866	5.76	4.2856E-09	ZBTB22

gout	hypothyroidism/myxoedema	ENSG00000204209	6	33286335	33297046	2	1	925866	5.43	2.7454E-08	DAXX
gout	hypothyroidism/myxoedema	ENSG00000237649	6	33359313	33377701	5	1	925866	6.11	5E-10	KIFC1
gout	hypothyroidism/myxoedema	ENSG00000112511	6	33378176	33384230	4	1	925866	6.17	3.3593E-10	PHF1
gout	hypothyroidism/myxoedema	ENSG00000112514	6	33384219	33386094	1	1	925866	5.5	1.9254E-08	CUTA
gout	hypothyroidism/myxoedema	ENSG00000197283	6	33387847	33421466	12	1	925866	6.07	6.5295E-10	SYNGAP1
gout	hypothyroidism/myxoedema	ENSG00000213588	6	33422356	33425325	1	1	925866	5.85	2.4162E-09	ZBTB9
gout	hypothyroidism/myxoedema	ENSG00000112182	6	90636248	91006627	24	2	925866	6.34	1.1142E-10	BACH2
gout	hypothyroidism/myxoedema	ENSG00000249141	6	167271582	167369612	1	1	925866	5.53	1.6478E-08	RP11-514O12.4
gout	hypothyroidism/myxoedema	ENSG00000026297	6	167342992	167370679	1	1	925866	5.53	1.6478E-08	RNASET2
gout	hypothyroidism/myxoedema	ENSG00000162302	11	64126620	64139687	3	1	925866	3.91	0.000046357	RPS6KA4

gout	hypothyroidism/myxoedema	ENSG00000168065	11	64323098	64340347	13	1	925866	5.41	3.0754E-08	SLC22A11
gout	hypothyroidism/myxoedema	ENSG00000197891	11	64358113	64369820	1	1	925866	6	9.7508E-10	SLC22A12
gout	hypothyroidism/myxoedema	ENSG00000065361	12	56473641	56497289	9	1	925866	4.71	1.2327E-06	ERBB3
gout	hypothyroidism/myxoedema	ENSG00000257411	12	56495115	56503073	1	1	925866	5.49	2.0136E-08	RP11-603J24.9
gout	hypothyroidism/myxoedema	ENSG00000111249	12	111471828	111788358	71	4	925866	7.39	7.3663E-14	CUX2
gout	hypothyroidism/myxoedema	ENSG00000198324	12	111798455	111806925	6	1	925866	6.99	1.3361E-12	FAM109A
gout	hypothyroidism/myxoedema	ENSG00000111252	12	111843752	111889427	23	1	925866	6.11	5E-10	SH2B3
gout	hypothyroidism/myxoedema	ENSG00000204842	12	111890018	112037480	70	2	925866	7.67	8.3267E-15	ATXN2
gout	hypothyroidism/myxoedema	ENSG00000089234	12	112079950	112123790	16	1	925866	5.5	1.8706E-08	BRAP
gout	hypothyroidism/myxoedema	ENSG00000111271	12	112123857	112194903	36	2	925866	6.03	8.0401E-10	ACAD10

gout	hypothyroidism/myxoedema	ENSG00000257767	12	112191694	112229222	19	2	925866	5.86	2.384E-09	RP11-162P23.2
gout	hypothyroidism/myxoedema	ENSG00000111275	12	112204691	112247782	20	1	925866	5.78	3.7846E-09	ALDH2
gout	hypothyroidism/myxoedema	ENSG00000089022	12	112279782	112334343	71	1	925866	5.63	9.1441E-09	MAPKAPK5
gout	hypothyroidism/myxoedema	ENSG00000198270	12	112369086	112450970	57	1	925866	6.18	3.1988E-10	TMEM116
gout	hypothyroidism/myxoedema	ENSG00000111300	12	112464500	112546826	10	2	925866	6.92	2.2158E-12	NAA25
gout	hypothyroidism/myxoedema	ENSG00000135148	12	112563305	112591407	2	1	925866	6.23	2.3563E-10	TRAFD1
gout	hypothyroidism/myxoedema	ENSG00000173064	12	112597992	112819896	19	2	925866	6.1	5.1731E-10	HECTD4
gout	hypothyroidism/myxoedema	ENSG00000089009	12	112842994	112856642	2	1	925866	5.27	6.8074E-08	RPL6
gout	hypothyroidism/myxoedema	ENSG00000179295	12	112856155	112947717	9	2	925866	7.51	2.8533E-14	PTPN11
gout	hypothyroidism/myxoedema	ENSG00000089169	12	113008184	113336686	34	3	925866	6.65	1.4546E-11	RPH3A

gout	hypothyroidism/myxoedema	ENSG00000175727	12	122516628	122631894	114	1	925866	5.89	1.9306E-09	MLXIP
gout	hypothyroidism/myxoedema	ENSG00000072818	17	7239848	7254797	1	1	925866	5.33	4.8866E-08	ACAP1
gout	hypothyroidism/myxoedema	ENSG00000127666	19	4815944	4831716	1	1	925866	5.51	1.793E-08	TICAM1
gout	hypothyroidism/myxoedema	ENSG00000171105	19	7112266	7294045	1	1	925866	5.4	3.3709E-08	INSR
hypothyroidism/myxoedema	hyperthyroidism/thyrotoxicosis	ENSG00000077549	1	19665267	19812066	27	1	925866	5.72	5.3093E-09	CAPZB
hypothyroidism/myxoedema	hyperthyroidism/thyrotoxicosis	ENSG00000081026	1	113933371	114228545	77	3	925866	6.11	5E-10	MAGI3
hypothyroidism/myxoedema	hyperthyroidism/thyrotoxicosis	ENSG00000116793	1	114239453	114302111	27	2	925866	6.11	5E-10	PHTF1
hypothyroidism/myxoedema	hyperthyroidism/thyrotoxicosis	ENSG00000081019	1	114304454	114355098	10	1	925866	6.11	5E-10	RSBN1
hypothyroidism/myxoedema	hyperthyroidism/thyrotoxicosis	ENSG00000134242	1	114356433	114414381	19	2	925866	6.11	5E-10	PTPN22

hypothyroidism/myxoedema	hyperthyroidism/thyrotoxicosis	ENSG00000188761	1	114420790	114430169	4	2	925866	6.39	8.389E-11	BCL2L15
hypothyroidism/myxoedema	hyperthyroidism/thyrotoxicosis	ENSG00000134262	1	114437370	114447823	4	1	925866	6.41	7.1938E-11	AP4B1
hypothyroidism/myxoedema	hyperthyroidism/thyrotoxicosis	ENSG00000118655	1	114447763	114456708	6	2	925866	8.06	3.8858E-16	DCLRE1B
hypothyroidism/myxoedema	hyperthyroidism/thyrotoxicosis	ENSG00000160856	1	157644111	157670647	1	1	925866	5.33	4.89E-08	FCRL3
hypothyroidism/myxoedema	hyperthyroidism/thyrotoxicosis	ENSG00000115705	2	1377995	1547483	5	1	925866	5.68	6.7535E-09	TPO
hypothyroidism/myxoedema	hyperthyroidism/thyrotoxicosis	ENSG00000078098	2	163027194	163101661	1	1	925866	5.54	1.55E-08	FAP
hypothyroidism/myxoedema	hyperthyroidism/thyrotoxicosis	ENSG00000115267	2	163123589	163175213	1	1	925866	5.86	2.33E-09	IFIH1
hypothyroidism/myxoedema	hyperthyroidism/thyrotoxicosis	ENSG00000138378	2	191894302	192016322	11	2	925866	6.5	3.9618E-11	STAT4

hypothyroidism/myxoedema	hyperthyroidism/thyrotoxicosis	ENSG00000178562	2	204571198	204603635	1	1	925866	5.73	5.07E-09	CD28
hypothyroidism/myxoedema	hyperthyroidism/thyrotoxicosis	ENSG00000163600	2	204801471	204826300	1	1	925866	5.86	2.29E-09	ICOS
hypothyroidism/myxoedema	hyperthyroidism/thyrotoxicosis	ENSG00000145012	3	187871072	188608460	21	2	925866	6.15	3.968E-10	LPP
hypothyroidism/myxoedema	hyperthyroidism/thyrotoxicosis	ENSG00000113231	5	76506274	76725632	4	1	925866	4.69	1.3551E-06	PDE8B
hypothyroidism/myxoedema	hyperthyroidism/thyrotoxicosis	ENSG00000137265	6	391739	411447	2	1	925866	4.9	4.8056E-07	IRF4
hypothyroidism/myxoedema	hyperthyroidism/thyrotoxicosis	ENSG00000124568	6	25783125	25832287	3	1	925866	6.11	5E-10	SLC17A1
hypothyroidism/myxoedema	hyperthyroidism/thyrotoxicosis	ENSG00000124564	6	25833294	25882514	4	1	925866	6.11	5E-10	SLC17A3
hypothyroidism/myxoedema	hyperthyroidism/thyrotoxicosis	ENSG00000112337	6	25912982	25930946	1	1	925866	7.75	4.42E-15	SLC17A2

hypothyroidism/myxoedema	hyperthyroidism/thyrotoxicosis	ENSG00000112343	6	25963030	25987384	2	1	925866	6.11	5E-10	TRIM38
hypothyroidism/myxoedema	hyperthyroidism/thyrotoxicosis	ENSG00000010704	6	26087509	26098571	2	1	925866	7.89	1.5487E-15	HFE
hypothyroidism/myxoedema	hyperthyroidism/thyrotoxicosis	ENSG00000180596	6	26115101	26124154	3	1	925866	5.74	4.7354E-09	HIST1H2BC
hypothyroidism/myxoedema	hyperthyroidism/thyrotoxicosis	ENSG00000186470	6	26365387	26378546	6	1	925866	6.11	5E-10	BTN3A2
hypothyroidism/myxoedema	hyperthyroidism/thyrotoxicosis	ENSG00000112763	6	26458150	26476849	3	1	925866	6.11	5E-10	BTN2A1
hypothyroidism/myxoedema	hyperthyroidism/thyrotoxicosis	ENSG00000181315	6	26636518	26659980	1	1	925866	10.3	5.37E-25	ZNF322
hypothyroidism/myxoedema	hyperthyroidism/thyrotoxicosis	ENSG00000158553	6	27253682	27279949	1	1	925866	5.84	2.63E-09	POM121L2
hypothyroidism/myxoedema	hyperthyroidism/thyrotoxicosis	ENSG00000124613	6	27342394	27371683	1	1	925866	11.1	1.06E-28	ZNF391

hypothyroidism/myxoedema	hyperthyroidism/thyrotoxicosis	ENSG00000096654	6	27418522	27440897	5	1	925866	6.11	5E-10	ZNF184
hypothyroidism/myxoedema	hyperthyroidism/thyrotoxicosis	ENSG00000233822	6	27806323	27823487	1	1	925866	10.9	4.17E-28	HIST1H2BN
hypothyroidism/myxoedema	hyperthyroidism/thyrotoxicosis	ENSG00000198315	6	28109688	28127250	2	1	925866	6.11	5E-10	ZKSCAN8
hypothyroidism/myxoedema	hyperthyroidism/thyrotoxicosis	ENSG00000137185	6	28192664	28201260	2	1	925866	5.4	3.2901E-08	ZSCAN9
hypothyroidism/myxoedema	hyperthyroidism/thyrotoxicosis	ENSG00000187626	6	28212401	28227011	3	1	925866	6.11	5E-10	ZKSCAN4
hypothyroidism/myxoedema	hyperthyroidism/thyrotoxicosis	ENSG00000137338	6	28249314	28270326	2	1	925866	6.11	5E-10	PGBD1
hypothyroidism/myxoedema	hyperthyroidism/thyrotoxicosis	ENSG00000235109	6	28292470	28324048	5	1	925866	6.92	2.2841E-12	ZSCAN31
hypothyroidism/myxoedema	hyperthyroidism/thyrotoxicosis	ENSG00000189298	6	28317691	28336947	1	1	925866	11.9	4.68E-33	ZKSCAN3

hypothyroidism/myxoedema	hyperthyroidism/thyrotoxicosis	ENSG00000198704	6	28471073	28495992	1	1	925866	12.1	9.43E-34	GPX6
hypothyroidism/myxoedema	hyperthyroidism/thyrotoxicosis	ENSG00000232040	6	28539407	28583989	1	1	925866	5.37	3.95E-08	SCAND3
hypothyroidism/myxoedema	hyperthyroidism/thyrotoxicosis	ENSG00000204681	6	29523406	29601753	13	3	925866	7.91	1.3323E-15	GABBR1
hypothyroidism/myxoedema	hyperthyroidism/thyrotoxicosis	ENSG00000204657	6	29555683	29556745	1	1	925866	6.07	6.21E-10	OR2H2
hypothyroidism/myxoedema	hyperthyroidism/thyrotoxicosis	ENSG00000204642	6	29690552	29706305	1	1	925866	5.43	2.88E-08	HLA-F
hypothyroidism/myxoedema	hyperthyroidism/thyrotoxicosis	ENSG00000206503	6	29909037	29913661	6	3	925866	8.01	5.5511E-16	HLA-A
hypothyroidism/myxoedema	hyperthyroidism/thyrotoxicosis	ENSG00000204616	6	30070674	30080883	1	1	925866	5.93	1.48E-09	TRIM31
hypothyroidism/myxoedema	hyperthyroidism/thyrotoxicosis	ENSG00000204610	6	30130993	30140473	1	1	925866	6.3	1.46E-10	TRIM15

hypothyroidism/myxoedema	hyperthyroidism/thyrotoxicosis	ENSG00000234127	6	30152232	30181204	5	2	925866	6.26	1.914E-10	TRIM26
hypothyroidism/myxoedema	hyperthyroidism/thyrotoxicosis	ENSG00000137312	6	30695486	30710510	2	1	925866	6.11	5E-10	FLOT1
hypothyroidism/myxoedema	hyperthyroidism/thyrotoxicosis	ENSG00000204580	6	30844198	30867933	10	2	925866	7.11	5.6466E-13	DDR1
hypothyroidism/myxoedema	hyperthyroidism/thyrotoxicosis	ENSG00000213780	6	30875961	30881883	2	1	925866	6.11	5E-10	GTF2H4
hypothyroidism/myxoedema	hyperthyroidism/thyrotoxicosis	ENSG00000137411	6	30876019	30894236	4	2	925866	6.58	2.3001E-11	VARS2
hypothyroidism/myxoedema	hyperthyroidism/thyrotoxicosis	ENSG00000168631	6	30908749	30921998	1	1	925866	5.34	4.54E-08	DPCR1
hypothyroidism/myxoedema	hyperthyroidism/thyrotoxicosis	ENSG00000261272	6	30978251	31003179	2	1	925866	8.39	2.48E-17	MUC22
hypothyroidism/myxoedema	hyperthyroidism/thyrotoxicosis	ENSG00000204542	6	31079000	31080336	3	1	925866	6.11	5E-10	C6orf15

hypothyroidism/myxoedema	hyperthyroidism/thyrotoxicosis	ENSG00000204540	6	31082527	31107869	26	6	925866	6.11	5E-10	PSORS1C1
hypothyroidism/myxoedema	hyperthyroidism/thyrotoxicosis	ENSG00000204539	6	31082867	31088223	11	2	925866	6.11	5E-10	CDSN
hypothyroidism/myxoedema	hyperthyroidism/thyrotoxicosis	ENSG00000204538	6	31105313	31107127	4	2	925866	6.75	7.5468E-12	PSORS1C2
hypothyroidism/myxoedema	hyperthyroidism/thyrotoxicosis	ENSG00000204536	6	31110216	31126015	31	5	925866	6.11	5E-10	CCHCR1
hypothyroidism/myxoedema	hyperthyroidism/thyrotoxicosis	ENSG00000137310	6	31126319	31134936	4	2	925866	7	1.3262E-12	TCF19
hypothyroidism/myxoedema	hyperthyroidism/thyrotoxicosis	ENSG00000204531	6	31132119	31148508	11	3	925866	7.83	2.4425E-15	POU5F1
hypothyroidism/myxoedema	hyperthyroidism/thyrotoxicosis	ENSG00000206344	6	31165537	31171745	3	1	925866	6.11	5E-10	HCG27
hypothyroidism/myxoedema	hyperthyroidism/thyrotoxicosis	ENSG00000204525	6	31236526	31239907	11	4	925866	7.71	6.1617E-15	HLA-C

hypothyroidism/myxoedema	hyperthyroidism/thyrotoxicosis	ENSG00000234745	6	31321649	31324965	7	3	925866	7.92	1.1657E-15	HLA-B
hypothyroidism/myxoedema	hyperthyroidism/thyrotoxicosis	ENSG00000204520	6	31371356	31383092	3	2	925866	6.11	5E-10	MICA
hypothyroidism/myxoedema	hyperthyroidism/thyrotoxicosis	ENSG00000204516	6	31462658	31478901	3	1	925866	6.23	2.2967E-10	MICB
hypothyroidism/myxoedema	hyperthyroidism/thyrotoxicosis	ENSG00000198563	6	31497996	31510225	4	2	925866	6.83	4.2596E-12	DDX39B
hypothyroidism/myxoedema	hyperthyroidism/thyrotoxicosis	ENSG00000254870	6	31497996	31514385	4	2	925866	6.83	4.2596E-12	ATP6V1G2-DDX39B
hypothyroidism/myxoedema	hyperthyroidism/thyrotoxicosis	ENSG00000204498	6	31514647	31526606	1	1	925866	5.95	1.38E-09	NFKBIL1
hypothyroidism/myxoedema	hyperthyroidism/thyrotoxicosis	ENSG00000226979	6	31539831	31542101	1	1	925866	11.1	4.44E-29	LTA
hypothyroidism/myxoedema	hyperthyroidism/thyrotoxicosis	ENSG00000204469	6	31588497	31605548	4	2	925866	6.11	5E-10	PRRC2A

hypothyroidism/myxoedema	hyperthyroidism/thyrotoxicosis	ENSG00000204463	6	31606805	31620482	1	1	925866	6.85	3.7E-12	BAG6
hypothyroidism/myxoedema	hyperthyroidism/thyrotoxicosis	ENSG00000263020	6	31633879	31641323	1	1	925866	12.2	1.05E-34	CSNK2B-LY6G5B-1181
hypothyroidism/myxoedema	hyperthyroidism/thyrotoxicosis	ENSG00000240053	6	31637944	31641553	1	1	925866	12.2	1.05E-34	LY6G5B
hypothyroidism/myxoedema	hyperthyroidism/thyrotoxicosis	ENSG00000204427	6	31654726	31671221	1	1	925866	8.15	1.78E-16	ABHD16A
hypothyroidism/myxoedema	hyperthyroidism/thyrotoxicosis	ENSG00000204422	6	31654739	31681849	1	1	925866	8.15	1.78E-16	XXbac-BPG32J3.20
hypothyroidism/myxoedema	hyperthyroidism/thyrotoxicosis	ENSG00000204410	6	31707725	31732622	1	1	925866	11.7	5.61E-32	MSH5
hypothyroidism/myxoedema	hyperthyroidism/thyrotoxicosis	ENSG00000255152	6	31707797	31732628	1	1	925866	11.7	5.61E-32	MSH5-SAPCD1
hypothyroidism/myxoedema	hyperthyroidism/thyrotoxicosis	ENSG00000204392	6	31765173	31774761	2	1	925866	6.11	5E-10	LSM2

hypothyroidism/myxoedema	hyperthyroidism/thyrotoxicosis	ENSG00000204390	6	31777396	31783437	1	1	925866	11.7	8E-32	HSPA1L
hypothyroidism/myxoedema	hyperthyroidism/thyrotoxicosis	ENSG00000204389	6	31783291	31785723	1	1	925866	11.8	1.15E-32	HSPA1A
hypothyroidism/myxoedema	hyperthyroidism/thyrotoxicosis	ENSG00000204371	6	31847536	31865464	1	1	925866	6.23	2.28E-10	EHMT2
hypothyroidism/myxoedema	hyperthyroidism/thyrotoxicosis	ENSG00000166278	6	31865562	31913449	2	1	925866	6.11	5E-10	C2
hypothyroidism/myxoedema	hyperthyroidism/thyrotoxicosis	ENSG00000244255	6	31895475	31919825	2	1	925866	14.9	9.9999E-51	CFB
hypothyroidism/myxoedema	hyperthyroidism/thyrotoxicosis	ENSG00000243649	6	31895475	31919861	2	1	925866	14.9	9.9999E-51	CFB
hypothyroidism/myxoedema	hyperthyroidism/thyrotoxicosis	ENSG00000204344	6	31938868	31950598	1	1	925866	8.57	5.3E-18	STK19
hypothyroidism/myxoedema	hyperthyroidism/thyrotoxicosis	ENSG00000168477	6	32008931	32083111	11	2	925866	7	1.2493E-12	TNXB

hypothyroidism/myxoedema	hyperthyroidism/thyrotoxicosis	ENSG00000213676	6	32065953	32096030	4	2	925866	6.63	1.65E-11	ATF6B
hypothyroidism/myxoedema	hyperthyroidism/thyrotoxicosis	ENSG00000258388	6	32121622	32139755	1	1	925866	6.05	7.27E-10	PPT2-EGFL8
hypothyroidism/myxoedema	hyperthyroidism/thyrotoxicosis	ENSG00000204310	6	32135989	32145873	1	1	925866	6.05	7.27E-10	AGPAT1
hypothyroidism/myxoedema	hyperthyroidism/thyrotoxicosis	ENSG00000204308	6	32146131	32151930	1	1	925866	13.5	1.2E-41	RNF5
hypothyroidism/myxoedema	hyperthyroidism/thyrotoxicosis	ENSG00000204301	6	32162620	32191844	2	1	925866	6.11	5E-10	NOTCH4
hypothyroidism/myxoedema	hyperthyroidism/thyrotoxicosis	ENSG00000204296	6	32256303	32339684	29	4	925866	6.11	5E-10	C6orf10
hypothyroidism/myxoedema	hyperthyroidism/thyrotoxicosis	ENSG00000204287	6	32407619	32412823	3	1	925866	12	1.814E-33	HLA-DRA
hypothyroidism/myxoedema	hyperthyroidism/thyrotoxicosis	ENSG00000198502	6	32485120	32498064	6	2	925866	6.11	5E-10	HLA-DRB5

hypothyroidism/myxoedema	hyperthyroidism/thyrotoxicosis	ENSG00000196126	6	32546546	32557625	6	3	925866	7.71	6.3283E-15	HLA-DRB1
hypothyroidism/myxoedema	hyperthyroidism/thyrotoxicosis	ENSG00000196735	6	32595956	32614839	16	4	925866	7.1	6.3982E-13	HLA-DQA1
hypothyroidism/myxoedema	hyperthyroidism/thyrotoxicosis	ENSG00000179344	6	32627244	32636160	23	5	925866	8.13	2.2204E-16	HLA-DQB1
hypothyroidism/myxoedema	hyperthyroidism/thyrotoxicosis	ENSG00000232629	6	32723875	32731311	2	1	925866	6.11	5E-10	HLA-DQB2
hypothyroidism/myxoedema	hyperthyroidism/thyrotoxicosis	ENSG00000241106	6	32780540	32784825	1	1	925866	12.4	1.27E-35	HLA-DOB
hypothyroidism/myxoedema	hyperthyroidism/thyrotoxicosis	ENSG00000250264	6	32781544	32806599	2	1	925866	6.11	5E-10	TAP2
hypothyroidism/myxoedema	hyperthyroidism/thyrotoxicosis	ENSG00000204267	6	32789610	32806557	2	1	925866	6.11	5E-10	TAP2
hypothyroidism/myxoedema	hyperthyroidism/thyrotoxicosis	ENSG00000240065	6	32811913	32827362	2	1	925866	6.11	5E-10	PSMB9

hypothyroidism/myxoedema	hyperthyroidism/thyrotoxicosis	ENSG00000168394	6	32812986	32821755	2	1	925866	6.11	5E-10	TAP1
hypothyroidism/myxoedema	hyperthyroidism/thyrotoxicosis	ENSG00000242574	6	32902406	32908847	1	1	925866	7.04	9.39E-13	HLA-DMB
hypothyroidism/myxoedema	hyperthyroidism/thyrotoxicosis	ENSG00000248993	6	32905141	32920899	2	1	925866	5.72	5.4197E-09	XXbac-BPG181M17.5
hypothyroidism/myxoedema	hyperthyroidism/thyrotoxicosis	ENSG00000204257	6	32916390	32936871	2	1	925866	5.72	5.4197E-09	HLA-DMA
hypothyroidism/myxoedema	hyperthyroidism/thyrotoxicosis	ENSG00000231389	6	33032346	33048552	23	1	925866	6.11	5E-10	HLA-DPA1
hypothyroidism/myxoedema	hyperthyroidism/thyrotoxicosis	ENSG00000223865	6	33043703	33054978	6	3	925866	7.8	3.2196E-15	HLA-DPB1
hypothyroidism/myxoedema	hyperthyroidism/thyrotoxicosis	ENSG00000112182	6	90636248	91006627	19	2	925866	7.79	3.3307E-15	BACH2
hypothyroidism/myxoedema	hyperthyroidism/thyrotoxicosis	ENSG00000026297	6	167342992	167370679	1	1	925866	8.62	3.39E-18	RNASET2

hypothyroidism/myxoedema	hyperthyroidism/thyrotoxicosis	ENSG00000213066	6	167412670	167466201	20	1	925866	6.75	7.5846E-12	FGFR1OP
hypothyroidism/myxoedema	hyperthyroidism/thyrotoxicosis	ENSG00000104415	8	134203282	134242587	4	1	925866	6.11	4.9213E-10	WISP1
hypothyroidism/myxoedema	hyperthyroidism/thyrotoxicosis	ENSG00000134460	10	6052652	6104288	9	2	925866	6.54	3.1372E-11	IL2RA
hypothyroidism/myxoedema	hyperthyroidism/thyrotoxicosis	ENSG00000150347	10	63661059	63856703	12	2	925866	7.75	4.7184E-15	ARID5B
hypothyroidism/myxoedema	hyperthyroidism/thyrotoxicosis	ENSG00000182010	10	63942794	64028466	5	1	925866	4.2	0.000013201	RTKN2
hypothyroidism/myxoedema	hyperthyroidism/thyrotoxicosis	ENSG00000111249	12	111471828	111788358	24	4	925866	7.73	5.4401E-15	CUX2
hypothyroidism/myxoedema	hyperthyroidism/thyrotoxicosis	ENSG00000198324	12	111798455	111806925	1	1	925866	6.39	8.22E-11	FAM109A
hypothyroidism/myxoedema	hyperthyroidism/thyrotoxicosis	ENSG00000111252	12	111843752	111889427	2	1	925866	4.27	0.000009865	SH2B3

hypothyroidism/myxoedema	hyperthyroidism/thyrotoxicosis	ENSG00000204842	12	111890018	112037480	10	1	925866	5.35	4.2862E-08	ATXN2	
hypothyroidism/myxoedema	hyperthyroidism/thyrotoxicosis	ENSG00000089234	12	112079950	112123790	2	1	925866	6.11	5E-10	BRAP	
hypothyroidism/myxoedema	hyperthyroidism/thyrotoxicosis	ENSG00000111275	12	112204691	112247782	1	1	925866	6.46	5.31E-11	ALDH2	
hypothyroidism/myxoedema	hyperthyroidism/thyrotoxicosis	ENSG00000173064	12	112597992	112819896	1	1	925866	11.2	2.57E-29	HECTD4	
hypothyroidism/myxoedema	hyperthyroidism/thyrotoxicosis	ENSG00000179295	12	112856155	112947717	3	1	925866	6.11	5E-10	PTPN11	
hypothyroidism/myxoedema	hyperthyroidism/thyrotoxicosis	ENSG00000100629	14	80943330	81425861	15	3	925866	6.99	1.4043E-12	CEP128	
hypothyroidism/myxoedema	hyperthyroidism/thyrotoxicosis	ENSG00000165409	14	81421333	81612646	5	1	925866	5.2	9.7933E-08	TSHR	
hypothyroidism/myxoedema	hyperthyroidism/thyrotoxicosis	ENSG00000172575	15	38780304	38857776	5	1	925866	6.11	5E-10	RASGRP1	

hypothyroidism/myxoedema	hyperthyroidism/thyrotoxicosis	ENSG00000267261	17	40271692	40306934	2	1	925866	6.01	9.287E-10	CTD-2132N18.3
hypothyroidism/myxoedema	hyperthyroidism/thyrotoxicosis	ENSG00000108774	17	40276994	40307035	2	1	925866	6.01	9.287E-10	RAB5C
hypothyroidism/myxoedema	hyperthyroidism/thyrotoxicosis	ENSG00000127666	19	4815944	4831716	4	1	925866	6.11	5E-10	TICAM1
hypothyroidism/myxoedema	hyperthyroidism/thyrotoxicosis	ENSG00000160185	21	43824008	43867791	1	1	925866	5.58	1.22E-08	UBASH3A
hypothyroidism/myxoedema	hyperthyroidism/thyrotoxicosis	ENSG00000176635	22	30476163	30573064	1	1	925866	5.38	3.73E-08	HORMAD2

Analysis of tissue-specific enrichment analysis

Trait pair	Trait pair	VARIABLE	TYPE	NGENES	BETA	BETA_STD	SE	P	FULL_NAME	SampleGroup
gout	hypothyroidism/myxoedema	Artery_Coronary	COVAR	158	0.37079	0.77617	0.11935	0.0012472	Artery_Coronary	Trait pair1
gout	hypothyroidism/myxoedema	Adipose_Subcutaneous	COVAR	158	0.29554	0.61618	0.11179	0.0047961	Adipose_Subcutaneous	Trait pair1
Type 2 diabetes	hypothyroidism/myxoedema	Cells_Cultured_fibroblasts	COVAR	49	0.3208	0.68106	0.1205	0.0059492	Cells_Cultured_fibroblasts	Trait pair2
gout	hypothyroidism/myxoedema	Artery_Tibial	COVAR	158	0.29402	0.62293	0.11977	0.0079546	Artery_Tibial	Trait pair1
gout	hypothyroidism/myxoedema	Artery_Aorta	COVAR	158	0.25936	0.5465	0.10589	0.0080723	Artery_Aorta	Trait pair1
gout	hypothyroidism/myxoedema	Adipose_Visceral_Omentum	COVAR	158	0.27068	0.55288	0.12275	0.014932	Adipose_Visceral_Omentum	Trait pair1
hypothyroidism/myxoedema	hyperthyroidism/thyrotoxicosis	Brain_Spinal_cord_cervical_c...	COVAR	120	0.66771	1.3191	0.31705	0.018843	Brain_Spinal_cord_cervical_c-1	Trait pair3
gout	hypothyroidism/myxoedema	Esophagus_Gastroesophageal_J...	COVAR	158	0.28761	0.58876	0.14284	0.023443	Esophagus_Gastroesophageal_Junction	Trait pair1
Type 2 diabetes	hypothyroidism/myxoedema	Cells_EBV-transformed_lympho...	COVAR	49	0.14665	0.34179	0.071944	0.024792	Cells_EBV-transformed_lymphocytes	Trait pair2

Type 2 diabetes	hypothyroidism/myxoedema	Whole_Blood	COVAR	49	0.25764	0.42335	0.13827	0.035675	Whole_Blood	Trait pair2
hypoglycemia	hypothyroidism/myxoedema	Muscle_Skeletal	COVAR	42	1.1214	1.8913	0.60607	0.036032	Muscle_Skeletal	Trait pair4
gout	hypothyroidism/myxoedema	Esophagus_Muscularis	COVAR	158	0.24215	0.49482	0.13485	0.037865	Esophagus_Muscularis	Trait pair1
hypothyroidism/myxoedema	hyperthyroidism/thyrotoxicosis	Liver	COVAR	120	0.26325	0.47176	0.1474	0.038558	Liver	Trait pair3
hypoglycemia	hypothyroidism/myxoedema	Brain_Caudate_basal_ganglia	COVAR	42	0.93173	1.6186	0.53551	0.044984	Brain_Caudate_basal_ganglia	Trait pair4
Type 2 diabetes	hypoglycemia	Thyroid	COVAR	66	0.28255	0.52273	0.16366	0.045047	Thyroid	Trait pair5
hypoglycemia	hypothyroidism/myxoedema	Brain_Putamen_basal_ganglia	COVAR	42	0.86626	1.485	0.50202	0.046278	Brain_Putamen_basal_ganglia	Trait pair4
Type 2 diabetes-hypothyroidism/myxoedema-hypoglycemia		Thyroid	COVAR	66	0.28255	0.52273	0.16366	0.045047	Thyroid	Trait pair6
		Brain_Caudate_basal_ganglia	COVAR	42	0.93173	1.6186	0.53551	0.044984	Brain_Caudate_basal_ganglia	Trait pair6
		Brain_Putamen_basal_ganglia	COVAR	42	0.86626	1.485	0.50202	0.046278	Brain_Putamen_basal_ganglia	Trait pair6
		Muscle_Skeletal	COVAR	42	1.1214	1.8913	0.60607	0.036032	Muscle_Skeletal	Trait pair6

Analysis of pathways

ID	Description	GeneRatio	BgRatio	pvalue	p.adjust	qvalue	geneID	Count	one_type	two_type
hsa04940	Type I diabetes mellitus	18/149	43/7914	1.27E-20	3.11E-18	2.64E-18	LTA/HLA-DQB1/HLA-DOB/HLA-DOA/HLA-B/HLA-DQA1/HLA-C/HLA-DRA/HLA-DQA2/HLA-DMA/HLA-DPA1/HLA-DPB1/HLA-DRB5/HLA-DRB1/HLA-DMB/CD28/HLA-F/HLA-A	18	Human Diseases	Endocrine and metabolic disease

hsa05320	Autoimmune thyroid disease	19/149	53/7914	3.57E-20	3.12E-18	2.65E-18	HLA-DQB1/HLA-DOB/HLA-DOA/HLA-B/HLA-DQA1/HLA-C/HLA-DRA/HLA-DQA2/HLA-DMA/HLA-DPA1/HLA-DPB1/HLA-DRB5/HLA-DRB1/HLA-DMB/TPO/CD28/HLA-F/HLA-A/TSHR	19	Human Diseases	Immune disease

hsa05330	Allograft rejection	17/149	38/7914	3.81E-20	3.12E-18	2.65E-18	HLA-DQB1/HLA-DOB/HLA-DOA/HLA-B/HLA-DQA1/HLA-C/HLA-DRA/HLA-DQA2/HLA-DMA/HLA-DPA1/HLA-DPB1/HLA-DRB5/HLA-DRB1/HLA-DMB/CD28/HLA-F/HLA-A	17	Human Diseases	Immune disease

hsa05322	Systemic lupus erythematosus	26/149	133/7914	8.46E-20	5.20E-18	4.41E-18	C2/HLA-DQB1/HLA-DOB/HLA-DOA/HLA-DQA1/HIST1H2BN/HLA-DRA/HLA-DQA2/HLA-DMA/HLA-DPA1/HLA-DPB1/HIST1H4A/HIST1H3C/HIST1H2BC/HIST1H2AC/HIST1H2BD/HIST1H2BF/HIST1H2BL/HIST1H2AJ/HIST1H2AL/HIST1H3I/HIST1H4L/HLA-DRB5/HLA-DRB1/HLA-DMB/CD28	26	Human Diseases	Immune disease

hsa05332	Graft-versus-host disease	17/149	41/7914	1.91E-19	9.41E-18	7.97E-18	HLA-DQB1/HLA-DOB/HLA-DOA/HLA-B/HLA-DQA1/HLA-C/HLA-DRA/HLA-DQA2/HLA-DMA/HLA-DPA1/HLA-DPB1/HLA-DRB5/HLA-DRB1/HLA-DMB/CD28/HLA-F/HLA-A	17	Human Diseases	Immune disease

hsa04612	Antigen processing and presentation	21/149	78/7914	3.09E-19	1.27E-17	1.08E-17	HSPA1L/HLA-DQB1/HLA-DOB/TAP2/TAP1/HLA-DOA/HLA-B/HSPA1A/HLA-DQA1/HLA-C/HLA-DRA/HLA-DQA2/HLA-DMA/HLA-DPA1/HLA-DPB1/HLA-DRB5/HLA-DRB1/HLA-DMB/TAPBP/HLA-F/HLA-A	21	Organismal Systems	Immune system

hsa05416	Viral myocarditis	17/149	60/7914	3.61E-16	1.27E-14	1.07E-14	HLA-DQB1/HLA-DOB/HLA-DOA/HLA-B/HLA-DQA1/HLA-C/HLA-DRA/HLA-DQA2/HLA-DMA/HLA-DPA1/HLA-DPB1/HLA-DRB5/HLA-DRB1/HLA-DMB/CD28/HLA-F/HLA-A	17	Human Diseases	Cardiovascular disease

hsa05310	Asthma	12/149	31/7914	1.32E-13	3.98E-12	3.37E-12	HLA-DQB1/HLA-DOB/HLA-DOA/HLA-DQA1/HLA-DRA/HLA-DQA2/HLA-DMA/HLA-DPA1/HLA-DPB1/HLA-DRB5/HLA-DRB1/HLA-DMB	12	Human Diseases	Immune disease

hsa04672	Intestinal immune network for IgA production	14/149	49/7914	1.46E-13	3.98E-12	3.37E-12	HLA-DQB1/HLA-DOB/HLA-DOA/HLA-DQA1/HLA-DRA/HLA-DQA2/HLA-DMA/HLA-DPA1/HLA-DPB1/HLA-DRB5/HLA-DRB1/HLA-DMB/CD28/ICOS	14	Organismal Systems	Immune system

hsa04145	Phagosome	21/149	152/7914	5.35E-13	1.32E-11	1.12E-11	ATP6V1G2/HLA-DQB1/HLA-DOB/TAP2/TAP1/HLA-DOA/HLA-B/HLA-DQA1/RAB5C/HLA-C/HLA-DRA/HLA-DQA2/HLA-DMA/HLA-DPA1/HLA-DPB1/TUBB/HLA-DRB5/HLA-DRB1/HLA-DMB/HLA-F/HLA-A	21	Cellular Processes	Transport and catabolism

hsa05169	Epstein-Barr virus infection	23/149	201/7914	2.22E-12	4.97E-11	4.21E-11	HLA-DQB1/HLA-DOB/TAP2/TAP1/HLA-DOA/BAK1/CCND2/HLA-B/HLA-DQA1/STAT3/HLA-C/HLA-DRA/HLA-DQA2/HLA-DMA/HLA-DPA1/HLA-DPB1/CD44/HLA-DRB5/HLA-DRB1/HLA-DMB/TAPBP/HLA-F/HLA-A	23	Human Diseases	Infectious disease: viral

hsa04514	Cell adhesion molecules (CAMs)	20/149	147/7914	2.69E-12	5.51E-11	4.67E-11	HLA-DQB1/HLA-DOB/HLA-DOA/HLA-B/HLA-DQA1/CLDN23/NTNG1/HLA-C/HLA-DRA/HLA-DQA2/HLA-DMA/HLA-DPA1/HLA-DPB1/HLA-DRB5/HLA-DRB1/HLA-DMB/CD28/ICOS/HLA-F/HLA-A	20	Environmental Information Processing	Signaling molecules and interaction

hsa05321	Inflammatory bowel disease (IBD)	14/149	65/7914	1.02E-11	1.92E-10	1.63E-10	HLA-DQB1/HLA-DOB/HLA-DOA/STAT4/HLA-DQA1/STAT3/HLA-DRA/HLA-DQA2/HLA-DMA/HLA-DPA1/HLA-DPB1/HLA-DRB5/HLA-DRB1/HLA-DMB	14	Human Diseases	Immune disease

hsa04640	Hematopoietic cell lineage	15/149	98/7914	3.12E-10	5.48E-09	4.64E-09	HLA-DQB1/HLA-DOB/HLA-DOA/HLA-DQA1/EPO/HLA-DRA/HLA-DQA2/HLA-DMA/HLA-DPA1/HLA-DPB1/CD44/HLA-DRB5/HLA-DRB1/HLA-DMB/IL2RA	15	Organismal Systems	Immune system

hsa05166	Human T-cell leukemia virus 1 infection	21/149	219/7914	6.28E-10	1.03E-08	8.72E-09	ADCY5/LTA/ATF6B/HLA-DQB1/HLA-DOB/HLA-DOA/CCND2/HLA-B/HLA-DQA1/HLA-C/HLA-DRA/HLA-DQA2/HLA-DMA/HLA-DPA1/HLA-DPB1/HLA-DRB5/HLA-DRB1/HLA-DMB/HLA-F/HLA-A/IL2RA	21	Human Diseases	Infectious disease: viral

hsa04659	Th17 cell differentiation	15/149	107/7914	1.11E-09	1.71E-08	1.45E-08	HLA-DQB1/HLA-DOB/HLA-DOA/HLA-DQA1/STAT3/HLA-DRA/HLA-DQA2/HLA-DMA/HLA-DPA1/HLA-DPB1/HLA-DRB5/HLA-DRB1/HLA-DMB/IRF4/IL2RA	15	Organismal Systems	Immune system

hsa04658	Th1 and Th2 cell differentiation	14/149	92/7914	1.36E-09	1.97E-08	1.67E-08	HLA-DQB1/HLA-DOB/HLA-DOA/STAT4/HLA-DQA1/HLA-DRA/HLA-DQA2/HLA-DMA/HLA-DPA1/HLA-DPB1/HLA-DRB5/HLA-DRB1/HLA-DMB/IL2RA	14	Organismal Systems	Immune system

hsa05323	Rheumatoid arthritis	14/149	93/7914	1.57E-09	2.15E-08	1.82E-08	ATP6V1G2/HLA-DQB1/HLA-DOB/HLA-DOA/HLA-DQA1/HLA-DRA/HLA-DQA2/HLA-DMA/HLA-DPA1/HLA-DPB1/HLA-DRB5/HLA-DRB1/HLA-DMB/CD28	14	Human Diseases	Immune disease

hsa05145	Toxoplasmosis	15/149	112/7914	2.14E-09	2.77E-08	2.34E-08	HSPA1L/HLA-DQB1/HLA-DOB/HLA-DOA/HSPA1A/HLA-DQA1/STAT3/HLA-DRA/HLA-DQA2/HLA-DMA/HLA-DPA1/HLA-DPB1/HLA-DRB5/HLA-DRB1/HLA-DMB	15	Human Diseases	Infectious disease: parasitic

hsa05150	Staphylococcus aureus infection	14/149	96/7914	2.42E-09	2.98E-08	2.52E-08	C2/CFB/HLA-DQB1/HLA-DOB/HLA-DOA/HLA-DQA1/HLA-DRA/HLA-DQA2/HLA-DMA/HLA-DPA1/HLA-DPB1/HLA-DRB5/HLA-DRB1/HLA-DMB	14	Human Diseases	Infectious disease: bacterial

hsa05140	Leishmaniasis	12/149	76/7914	1.40E-08	1.64E-07	1.39E-07	HLA-DQB1/HLA-DOB/HLA-DOA/HLA-DQA1/HLA-DRA/HLA-DQA2/HLA-DMA/HLA-DPA1/HLA-DPB1/HLA-DRB5/HLA-DRB1/HLA-DMB	12	Human Diseases	Infectious disease: parasitic

hsa05168	Herpes simplex virus 1 infection	27/149	491/7914	3.59E-07	4.01E-06	3.40E-06	LTA/HLA-DQB1/HLA-DOB/TAP2/TAP1/HLA-DOA/BAK1/EIF2B4/HLA-B/HLA-DQA1/PTPN11/HLA-C/HLA-DRA/HLA-DQA2/HLA-DMA/HLA-DPA1/HLA-DPB1/ZNF184/HLA-DRB5/HLA-DRB1/HLA-DMB/TAPBP/DAXX/TICAM1/IFIH1/HLA-F/HLA-A	27	Human Diseases	Infectious disease: viral

hsa05164	Influenza A	15/149	170/7914	6.19E-07	6.62E-06	5.61E-06	HLA-DQB1/HLA-DOB/HLA-DOA/BAK1/HLA-DQA1/HLA-DRA/HLA-DQA2/HLA-DMA/HLA-DPA1/HLA-DPB1/HLA-DRB5/HLA-DRB1/HLA-DMB/TICAM1/IFIH1	15	Human Diseases	Infectious disease: viral

hsa05203	Viral carcinogenesis	16/149	201/7914	1.03E-06	1.06E-05	8.98E-06	ATF6B/BAK1/CCND2/HLA-B/STAT3/HIST1H2BN/HLA-C/HIST1H4A/HIST1H2BC/HIST1H2BD/HIST1H2BF/HIST1H2BL/HIST1H4L/GTF2H4/HLA-F/HLA-A	16	Human Diseases	Cancer: overview
hsa05034	Alcoholism	14/149	184/7914	8.60E-06	8.46E-05	7.17E-05	ADCY5/ATF6B/HIST1H2BN/HIST1H4A/HIST1H3C/HIST1H2BC/HIST1H2AC/HIST1H2BD/HIST1H2BF/HIST1H2BL/HIST1H2AJ/HIST1H2AL/HIST1H3I/HIST1H4L	14	Human Diseases	Substance dependence

hsa05152	Tuberculosis	13/149	179/7914	3.00E-05	0.000284239	0.000240819	HLA-DQB1/HLA-DOB/HLA-DOA/HLA-DQA1/RAB5C/HLA-DRA/HLA-DQA2/HLA-DMA/HLA-DPA1/HLA-DPB1/HLA-DRB5/HLA-DRB1/HLA-DMB	13	Human Diseases	Infectious disease: bacterial
hsa04218	Cellular senescence	10/149	160/7914	0.000844098	0.00769067	0.006515844	CCND2/MRAS/HIPK1/ZFP36L2/HLA-B/GATA4/HLA-C/ITPR3/HLA-F/HLA-A	10	Cellular Processes	Cell growth and death

hsa05163	Human cytomegalovirus infection	12/149	225/7914	0.001064677	0.009353948	0.007925039	ADCY5/ATF6B/TAP2/TAP1/BAK1/HLA-B/STAT3/HLA-C/ITPR3/TAPBP/HLA-F/HLA-A	12	Human Diseases	Infectious disease: viral
hsa05162	Measles	9/149	138/7914	0.001136336	0.009639261	0.008166768	HSPA1L/BAK1/CCND2/HSPA1A/STAT3/CSNK2B/IFIH1/CD28/IL2RA	9	Human Diseases	Infectious disease: viral
hsa04918	Thyroid hormone synthesis	6/149	74/7914	0.002603337	0.020762984	0.017591232	ADCY5/ATF6B/ITPR3/GPX6/TPO/TSHR	6	Organismal Systems	Endocrine system
hsa05167	Kaposi sarcoma-associated herpesvirus infection	10/149	186/7914	0.002616474	0.020762984	0.017591232	MICB/BAK1/HLA-B/STAT3/HLA-C/MICA/ITPR3/TICAM1/HLA-F/HLA-A	10	Human Diseases	Infectious disease: viral

hsa04650	Natural killer cell mediated cytotoxicity	8/149	131/7914	0.003215677	0.02472052	0.020944215	MICB/NCR3/VAV3/HLA-B/PTPN11/HLA-C/MICA/HLA-A	8	Organismal Systems	Immune system
hsa04144	Endocytosis	11/149	243/7914	0.006039508	0.045021785	0.038144259	HSPA1L/ARAP1/HLA-B/HSPA1A/RAB5C/HLA-C/ACAP1/CAPZB/HLA-F/HLA-A/IL2RA	11	Cellular Processes	Transport and catabolism
hsa05235	PD-L1 expression and PD-1 checkpoint pathway in cancer	6/149	89/7914	0.00649062	0.046961547	0.039787704	PTPN11/RASGRP1/STAT3/CSNK2B/TICAM1/CD28	6	Human Diseases	Cancer: overview
**ID**	**Description**	**GeneRatio**	**BgRatio**	**pvalue**	**p.adjust**	**qvalue**	**geneID**	**Count**	**one_type**	**two_type**

hsa04612	Antigen processing and presentation	15/83	78/7914	1.41E-15	3.00E-13	2.42E-13	HSPA1L/HLA-DQB1/HLA-DOB/TAP2/TAP1/HLA-DOA/HLA-B/HSPA1A/HLA-DQA1/HLA-C/HLA-DRA/HLA-DQA2/HLA-DMA/HLA-DPA1/HLA-DPB1	15	Organismal Systems	Immune system
hsa04940	Type I diabetes mellitus	30651	43/7914	9.18E-15	9.78E-13	7.88E-13	LTA/HLA-DQB1/HLA-DOB/HLA-DOA/HLA-B/HLA-DQA1/HLA-C/HLA-DRA/HLA-DQA2/HLA-DMA/HLA-DPA1/HLA-DPB1	12	Human Diseases	Endocrine and metabolic disease

hsa05330	Allograft rejection	30621	38/7914	8.16E-14	5.79E-12	4.67E-12	HLA-DQB1/HLA-DOB/HLA-DOA/HLA-B/HLA-DQA1/HLA-C/HLA-DRA/HLA-DQA2/HLA-DMA/HLA-DPA1/HLA-DPB1	11	Human Diseases	Immune disease
hsa05332	Graft-versus-host disease	30621	41/7914	2.09E-13	1.11E-11	8.96E-12	HLA-DQB1/HLA-DOB/HLA-DOA/HLA-B/HLA-DQA1/HLA-C/HLA-DRA/HLA-DQA2/HLA-DMA/HLA-DPA1/HLA-DPB1	11	Human Diseases	Immune disease

hsa05320	Autoimmune thyroid disease	30621	53/7914	4.56E-12	1.94E-10	1.56E-10	HLA-DQB1/HLA-DOB/HLA-DOA/HLA-B/HLA-DQA1/HLA-C/HLA-DRA/HLA-DQA2/HLA-DMA/HLA-DPA1/HLA-DPB1	11	Human Diseases	Immune disease
hsa05310	Asthma	30560	31/7914	1.65E-11	5.14E-10	4.14E-10	HLA-DQB1/HLA-DOB/HLA-DOA/HLA-DQA1/HLA-DRA/HLA-DQA2/HLA-DMA/HLA-DPA1/HLA-DPB1	9	Human Diseases	Immune disease

hsa05169	Epstein-Barr virus infection	17/83	201/7914	1.81E-11	5.14E-10	4.14E-10	HLA-DQB1/HLA-DOB/TAP2/TAP1/HLA-DOA/BAK1/CCND2/HLA-B/HLA-DQA1/STAT3/HLA-C/HLA-DRA/HLA-DQA2/HLA-DMA/HLA-DPA1/HLA-DPB1/CD44	17	Human Diseases	Infectious disease: viral

hsa05416	Viral myocarditis	30621	60/7914	1.93E-11	5.14E-10	4.14E-10	HLA-DQB1/HLA-DOB/HLA-DOA/HLA-B/HLA-DQA1/HLA-C/HLA-DRA/HLA-DQA2/HLA-DMA/HLA-DPA1/HLA-DPB1	11	Human Diseases	Cardiovascular disease
hsa04145	Phagosome	15/83	152/7914	3.53E-11	8.35E-10	6.73E-10	ATP6V1G2/HLA-DQB1/HLA-DOB/TAP2/TAP1/HLA-DOA/HLA-B/HLA-DQA1/RAB5C/HLA-C/HLA-DRA/HLA-DQA2/HLA-DMA/HLA-DPA1/HLA-DPB1	15	Cellular Processes	Transport and catabolism

hsa05321	Inflammatory bowel disease (IBD)	30621	65/7914	4.84E-11	1.03E-09	8.30E-10	HLA-DQB1/HLA-DOB/HLA-DOA/STAT4/HLA-DQA1/STAT3/HLA-DRA/HLA-DQA2/HLA-DMA/HLA-DPA1/HLA-DPB1	11	Human Diseases	Immune disease
hsa04672	Intestinal immune network for IgA production	30560	49/7914	1.44E-09	2.63E-08	2.12E-08	HLA-DQB1/HLA-DOB/HLA-DOA/HLA-DQA1/HLA-DRA/HLA-DQA2/HLA-DMA/HLA-DPA1/HLA-DPB1	9	Organismal Systems	Immune system

hsa05145	Toxoplasmosis	30651	112/7914	1.48E-09	2.63E-08	2.12E-08	HSPA1L/HLA-DQB1/HLA-DOB/HLA-DOA/HSPA1A/HLA-DQA1/STAT3/HLA-DRA/HLA-DQA2/HLA-DMA/HLA-DPA1/HLA-DPB1	12	Human Diseases	Infectious disease: parasitic
hsa04514	Cell adhesion molecules (CAMs)	13/83	147/7914	3.19E-09	5.22E-08	4.21E-08	HLA-DQB1/HLA-DOB/HLA-DOA/HLA-B/HLA-DQA1/CLDN23/NTNG1/HLA-C/HLA-DRA/HLA-DQA2/HLA-DMA/HLA-DPA1/HLA-DPB1	13	Environmental Information Processing	Signaling molecules and interaction

hsa05150	Staphylococcus aureus infection	30621	96/7914	3.67E-09	5.59E-08	4.50E-08	C2/CFB/HLA-DQB1/HLA-DOB/HLA-DOA/HLA-DQA1/HLA-DRA/HLA-DQA2/HLA-DMA/HLA-DPA1/HLA-DPB1	11	Human Diseases	Infectious disease: bacterial
hsa04640	Hematopoietic cell lineage	30621	98/7914	4.59E-09	6.51E-08	5.25E-08	HLA-DQB1/HLA-DOB/HLA-DOA/HLA-DQA1/EPO/HLA-DRA/HLA-DQA2/HLA-DMA/HLA-DPA1/HLA-DPB1/CD44	11	Organismal Systems	Immune system

hsa05166	Human T-cell leukemia virus 1 infection	15/83	219/7914	6.09E-09	8.10E-08	6.53E-08	ADCY5/LTA/ATF6B/HLA-DQB1/HLA-DOB/HLA-DOA/CCND2/HLA-B/HLA-DQA1/HLA-C/HLA-DRA/HLA-DQA2/HLA-DMA/HLA-DPA1/HLA-DPB1	15	Human Diseases	Infectious disease: viral
hsa04658	Th1 and Th2 cell differentiation	30590	92/7914	3.32E-08	4.16E-07	3.35E-07	HLA-DQB1/HLA-DOB/HLA-DOA/STAT4/HLA-DQA1/HLA-DRA/HLA-DQA2/HLA-DMA/HLA-DPA1/HLA-DPB1	10	Organismal Systems	Immune system

hsa05323	Rheumatoid arthritis	30590	93/7914	3.69E-08	4.37E-07	3.52E-07	ATP6V1G2/HLA-DQB1/HLA-DOB/HLA-DOA/HLA-DQA1/HLA-DRA/HLA-DQA2/HLA-DMA/HLA-DPA1/HLA-DPB1	10	Human Diseases	Immune disease
hsa05140	Leishmaniasis	30560	76/7914	7.95E-08	8.91E-07	7.18E-07	HLA-DQB1/HLA-DOB/HLA-DOA/HLA-DQA1/HLA-DRA/HLA-DQA2/HLA-DMA/HLA-DPA1/HLA-DPB1	9	Human Diseases	Infectious disease: parasitic

hsa05322	Systemic lupus erythematosus	30621	133/7914	1.15E-07	1.22E-06	9.86E-07	C2/HLA-DQB1/HLA-DOB/HLA-DOA/HLA-DQA1/HIST1H2BN/HLA-DRA/HLA-DQA2/HLA-DMA/HLA-DPA1/HLA-DPB1	11	Human Diseases	Immune disease
hsa04659	Th17 cell differentiation	30590	107/7914	1.43E-07	1.45E-06	1.17E-06	HLA-DQB1/HLA-DOB/HLA-DOA/HLA-DQA1/STAT3/HLA-DRA/HLA-DQA2/HLA-DMA/HLA-DPA1/HLA-DPB1	10	Organismal Systems	Immune system

hsa05164	Influenza A	30590	170/7914	1.01E-05	9.77E-05	7.87E-05	HLA-DQB1/HLA-DOB/HLA-DOA/BAK1/HLA-DQA1/HLA-DRA/HLA-DQA2/HLA-DMA/HLA-DPA1/HLA-DPB1	10	Human Diseases	Infectious disease: viral
hsa05152	Tuberculosis	30590	179/7914	1.59E-05	0.000147116	0.000118507	HLA-DQB1/HLA-DOB/HLA-DOA/HLA-DQA1/RAB5C/HLA-DRA/HLA-DQA2/HLA-DMA/HLA-DPA1/HLA-DPB1	10	Human Diseases	Infectious disease: bacterial

hsa05168	Herpes simplex virus 1 infection	16/83	491/7914	4.05E-05	0.000359381	0.000289494	LTA/HLA-DQB1/HLA-DOB/TAP2/TAP1/HLA-DOA/BAK1/HLA-B/HLA-DQA1/PTPN11/HLA-C/HLA-DRA/HLA-DQA2/HLA-DMA/HLA-DPA1/HLA-DPB1	16	Human Diseases	Infectious disease: viral
hsa04650	Natural killer cell mediated cytotoxicity	30498	131/7914	0.000423481	0.003608056	0.002906416	MICB/NCR3/VAV3/HLA-B/PTPN11/HLA-C/MICA	7	Organismal Systems	Immune system
hsa05163	Human cytomegalovirus infection	30560	225/7914	0.000546051	0.00447342	0.003603496	ADCY5/ATF6B/TAP2/TAP1/BAK1/HLA-B/STAT3/HLA-C/ITPR3	9	Human Diseases	Infectious disease: viral

hsa04218	Cellular senescence	30498	160/7914	0.001386841	0.010940632	0.008813061	CCND2/HIPK1/ZFP36L2/HLA-B/GATA4/HLA-C/ITPR3	7	Cellular Processes	Cell growth and death
hsa04911	Insulin secretion	30437	86/7914	0.002013382	0.015316084	0.012337641	ADCY5/ATF6B/KCNJ11/ABCC8/ITPR3	5	Organismal Systems	Endocrine system
hsa04915	Estrogen signaling pathway	30468	138/7914	0.003168404	0.023150933	0.018648886	ADCY5/HSPA1L/ATF6B/HSPA1A/GABBR1/ITPR3	6	Organismal Systems	Endocrine system
hsa05167	Kaposi sarcoma-associated herpesvirus infection	30498	186/7914	0.003260695	0.023150933	0.018648886	MICB/BAK1/HLA-B/STAT3/HLA-C/MICA/ITPR3	7	Human Diseases	Infectious disease: viral
hsa04927	Cortisol synthesis and secretion	30407	65/7914	0.004686823	0.032203009	0.02594065	ADCY5/ATF6B/PDE8B/ITPR3	4	Organismal Systems	Endocrine system
hsa05203	Viral carcinogenesis	30498	201/7914	0.004992522	0.033231472	0.026769113	ATF6B/BAK1/CCND2/HLA-B/STAT3/HIST1H2BN/HLA-C	7	Human Diseases	Cancer: overview

hsa05216	Thyroid cancer	30376	37/7914	0.006685585	0.043152411	0.034760776	PPARG/BAK1/TCF7L2	3	Human Diseases	Cancer: specific types
hsa04141	Protein processing in endoplasmic reticulum	30468	166/7914	0.00775974	0.04861249	0.03915906	WFS1/SSR1/HSPA1L/ATF6B/BAK1/HSPA1A	6	Genetic Information Processing	Folding, sorting and degradation

Mendelian Randomization Analysis of nine metabolic disorders and Mediation MR

exposure	outcome	method	SNP	pval	lo_ci	up_ci	or	or_lci95	or_uci95	FDR
Cushing syndrome	Type 2 diabetes	Wald ratio	1	0.416504787	-0.02465678	0.059573782	1.017611793	0.975644718	1.06138407	0.41650479
osteoporosis	Type 2 diabetes	MR Egger	12	0.661951404	-27.0041833	16.91030628	0.006428986	1.87E-12	22082727.1	0.95571186
osteoporosis	Type 2 diabetes	Weighted median	12	0.800140298	-3.83997506	2.961460254	0.644514859	0.021494137	19.3261723	0.95571186
osteoporosis	Type 2 diabetes	Inverse variance weighted	12	0.955711861	-3.14769517	3.331273047	1.096133446	0.042951008	27.9739312	0.95571186
osteoporosis	Type 2 diabetes	Simple mode	12	0.585534527	-7.23914017	4.013825552	0.199357156	0.000717929	55.3582418	0.95571186
osteoporosis	Type 2 diabetes	Weighted mode	12	0.702359111	-6.10418655	4.068329456	0.361342671	0.002233497	58.4592223	0.95571186
gout	Type 2 diabetes	MR Egger	13	0.761137732	-8.19673044	5.947728418	0.324814506	0.000275553	382.882601	0.83465444
gout	Type 2 diabetes	Weighted median	13	0.83465444	-1.83785747	1.484078313	0.8378723	0.159158063	4.41089807	0.83465444
gout	Type 2 diabetes	Inverse variance weighted	13	0.437516636	-6.08844407	2.633510609	0.177734088	0.002268936	13.9225609	0.83465444
gout	Type 2 diabetes	Simple mode	13	0.078229028	-0.06653796	7.431942872	39.75368165	0.9356274	1689.08607	0.39114514
gout	Type 2 diabetes	Weighted mode	13	0.717021925	-1.4468786	2.122768841	1.402063554	0.235303619	8.35423705	0.83465444
hypothyroidism/myxoedema	Type 2 diabetes	MR Egger	70	0.004761965	0.695820301	3.540972487	8.317788332	2.005353393	34.5004541	0.00793661

hypothyroidism/myxoedema	Type 2 diabetes	Weighted median	70	0.003882895	0.364913215	1.907151479	3.114387011	1.440388998	6.73387985	0.00793661
hypothyroidism/myxoedema	Type 2 diabetes	Inverse variance weighted	70	0.014271197	0.171368626	1.541300725	2.354514798	1.186928201	4.67066157	0.017839
hypothyroidism/myxoedema	Type 2 diabetes	Simple mode	70	0.081130459	-0.18598343	3.652885358	5.660153222	0.830287351	38.5858395	0.08113046
hypothyroidism/myxoedema	Type 2 diabetes	Weighted mode	70	6.38E-05	1.220824538	3.302932976	9.601110385	3.389981749	27.1922764	0.00031892
hyperthyroidism/thyrotoxicosis	Type 2 diabetes	MR Egger	8	0.835774946	-16.2304355	20.26117972	7.503518936	8.94E-08	629968089	0.9390261
hyperthyroidism/thyrotoxicosis	Type 2 diabetes	Weighted median	8	0.860006455	-5.29168611	4.417984451	0.646067798	0.005033266	82.9289694	0.9390261
hyperthyroidism/thyrotoxicosis	Type 2 diabetes	Inverse variance weighted	8	0.102864578	-1.03772768	11.33127417	171.8759924	0.354258756	83389.2069	0.51432289
hyperthyroidism/thyrotoxicosis	Type 2 diabetes	Simple mode	8	0.615461658	-6.58996854	11.41823961	11.18010137	0.001374083	90965.8653	0.9390261
hyperthyroidism/thyrotoxicosis	Type 2 diabetes	Weighted mode	8	0.9390261	-4.69138379	4.32659973	0.833274607	0.009173982	75.686494	0.9390261
Hypertension	Type 2 diabetes	MR Egger	3	0.90449976	-32.6598687	38.11795499	15.31822247	6.55E-15	3.5844E+16	0.90449976

Hypertension	Type 2 diabetes	Weighted median	3	0.33660569	-21.821869	7.464280858	0.000762587	3.33E-10	1744.60048	0.50898258
Hypertension	Type 2 diabetes	Inverse variance weighted	3	0.357262855	-18.5461127	6.692009014	0.002666331	8.82E-09	805.939771	0.50898258
Hypertension	Type 2 diabetes	Simple mode	3	0.407186066	-27.7748062	8.505406742	6.54E-05	8.66E-13	4941.41369	0.50898258
Hypertension	Type 2 diabetes	Weighted mode	3	0.387805046	-26.9946816	7.644049952	6.28E-05	1.89E-12	2088.18376	0.50898258
Disorders of lipoid metabolism	Type 2 diabetes	MR Egger	3	0.606647667	-0.3166004	0.148097949	0.919200302	0.728621856	1.15962647	0.75830958
Disorders of lipoid metabolism	Type 2 diabetes	Weighted median	3	0.049215937	0.000157238	0.091931326	1.047120779	1.000157251	1.09628953	0.24607968
Disorders of lipoid metabolism	Type 2 diabetes	Inverse variance weighted	3	0.227061786	-0.02558814	0.10779016	1.041957347	0.974736459	1.113814	0.37843631
Disorders of lipoid metabolism	Type 2 diabetes	Simple mode	3	0.21847657	-0.00909614	0.18013713	1.089283888	0.990945108	1.19738155	0.37843631
Disorders of lipoid metabolism	Type 2 diabetes	Weighted mode	3	0.796921281	-0.06204286	0.045890878	0.991956532	0.939842604	1.04696016	0.79692128
hypoglycemia	Type 2 diabetes	MR Egger	3	0.612853882	-2.48696733	1.183369347	0.521107466	0.083161787	3.26535781	0.61285388
hypoglycemia	Type 2 diabetes	Weighted median	3	4.29E-08	0.110908834	0.234471015	1.188497524	1.117293043	1.26423983	2.14E-07
hypoglycemia	Type 2 diabetes	Inverse variance weighted	3	0.210733266	-0.34364843	1.557833782	1.835088436	0.709178208	4.74852376	0.26341658

hypoglycemia	Type 2 diabetes	Simple mode	3	0.028369859	0.13406651	0.27057129	1.224238356	1.14346887	1.31071303	0.05817774
hypoglycemia	Type 2 diabetes	Weighted mode	3	0.034906645	0.102939376	0.227053845	1.179389121	1.10842421	1.25489744	0.05817774
Cushing syndrome	hypoglycemia	MR Egger	5	0.849632146	-0.19230684	0.155649864	0.981838458	0.825053671	1.16841703	0.9746397
Cushing syndrome	hypoglycemia	Weighted median	5	0.965403705	-0.06368944	0.060931649	0.998622055	0.938296352	1.06282627	0.9746397
Cushing syndrome	hypoglycemia	Inverse variance weighted	5	0.974639698	-0.05182056	0.050166404	0.999173265	0.949499231	1.05144605	0.9746397
Cushing syndrome	hypoglycemia	Simple mode	5	0.933468899	-0.08018351	0.087798767	1.003814884	0.922946956	1.0917684	0.9746397
Cushing syndrome	hypoglycemia	Weighted mode	5	0.637107091	-0.10628644	0.062416545	0.978303876	0.899167057	1.06440563	0.9746397
osteoporosis	hypoglycemia	MR Egger	14	0.160185681	-63.305578	8.475306335	1.24E-12	3.21E-28	4794.89139	0.73312696
osteoporosis	hypoglycemia	Weighted median	14	0.733126959	-9.58595396	6.744965917	0.241594634	6.87E-05	849.770164	0.73312696
osteoporosis	hypoglycemia	Inverse variance weighted	14	0.350974806	-3.8372977	10.80517208	32.58777411	0.021551762	49274.9973	0.73312696
osteoporosis	hypoglycemia	Simple mode	14	0.666597038	-20.9598214	13.26310121	0.021314662	7.89E-10	575561.412	0.73312696
osteoporosis	hypoglycemia	Weighted mode	14	0.622947957	-17.1717344	10.15104928	0.029886674	3.49E-08	25617.9686	0.73312696
gout	hypoglycemia	MR Egger	18	0.971078111	-10.2481054	9.870093558	0.827781621	3.54E-05	19343.1486	0.97107811
gout	hypoglycemia	Weighted median	18	0.344445375	-2.27072523	6.502587655	8.297308725	0.103237282	666.865019	0.65911786

gout	hypoglycemia	Inverse variance weighted	18	0.427841595	-3.38081513	7.974414041	9.942310681	0.034019713	2905.65478	0.65911786
gout	hypoglycemia	Simple mode	18	0.425191879	-12.1081081	4.983212145	0.028369292	5.51E-06	145.942418	0.65911786
gout	hypoglycemia	Weighted mode	18	0.527294286	-3.08795416	6.119884892	4.553815105	0.045595139	454.812339	0.65911786
hypothyroidism/myxoedema	hypoglycemia	MR Egger	98	9.72E-05	2.951966325	8.441009255	297.8195572	19.14355921	4633.22874	0.0001215
hypothyroidism/myxoedema	hypoglycemia	Weighted median	98	1.93E-09	3.543141288	6.977684917	192.5610223	34.57535994	1072.43272	3.21E-09
hypothyroidism/myxoedema	hypoglycemia	Inverse variance weighted	98	5.24E-11	3.057614245	5.660921392	78.19985695	21.27673548	287.413341	2.62E-10
hypothyroidism/myxoedema	hypoglycemia	Simple mode	98	0.776673527	-6.82583079	5.095700964	0.421024223	0.001085374	163.318285	0.77667353
hypothyroidism/myxoedema	hypoglycemia	Weighted mode	98	5.81E-10	5.94831222	10.68491563	4091.283135	383.106194	43691.796	1.45E-09
hyperthyroidism/thyrotoxicosis	hypoglycemia	MR Egger	9	0.66119682	-52.2047282	83.99345956	7995310.144	2.13E-23	3.01E+36	0.82649602
hyperthyroidism/thyrotoxicosis	hypoglycemia	Weighted median	9	0.177835414	-4.64038348	25.05563652	27109.1481	0.009653995	7.6125E+10	0.30697068

hyperthyroidism/thyrotoxicosis	hypoglycemia	Inverse variance weighted	9	0.017233741	4.883464193	50.28058571	9.52185E+11	132.0874486	6.86E+21	0.0861687
hyperthyroidism/thyrotoxicosis	hypoglycemia	Simple mode	9	0.184182408	-8.29429463	55.88760292	21615017084	0.000249939	1.87E+24	0.30697068
hyperthyroidism/thyrotoxicosis	hypoglycemia	Weighted mode	9	0.842529273	-11.6506163	14.37560663	3.905927137	8.71E-06	1750840.7	0.84252927
Hypertension	hypoglycemia	MR Egger	11	0.664346863	-59.3972863	37.27308762	1.57E-05	1.60E-26	1.5399E+16	0.66434686
Hypertension	hypoglycemia	Weighted median	11	0.090971339	-37.0253533	2.735637249	3.58E-08	8.32E-17	15.4195664	0.26275404
Hypertension	hypoglycemia	Inverse variance weighted	11	0.124290559	-27.1278539	3.281309863	6.63E-06	1.65E-12	26.6106061	0.26275404
Hypertension	hypoglycemia	Simple mode	11	0.210203235	-44.9555374	8.461955717	1.19E-08	2.99E-20	4731.30204	0.26275404
Hypertension	hypoglycemia	Weighted mode	11	0.186585067	-42.8761775	6.879147934	1.53E-08	2.39E-19	971.797971	0.26275404
Disorders of lipoid metabolism	hypoglycemia	MR Egger	6	0.101581012	0.018147308	0.468643762	1.275573058	1.018312971	1.59782569	0.49641089
Disorders of lipoid metabolism	hypoglycemia	Weighted median	6	0.524539477	-0.06418929	0.125909756	1.031341345	0.93782746	1.13417981	0.65567435
Disorders of lipoid metabolism	hypoglycemia	Inverse variance weighted	6	0.37970915	-0.0486126	0.127577377	1.040272179	0.952550079	1.13607277	0.63284858

Disorders of lipoid metabolism	hypoglycemia	Simple mode	6	0.832884117	-0.18654934	0.148545461	0.98117746	0.829817619	1.16014554	0.83288412
Disorders of lipoid metabolism	hypoglycemia	Weighted mode	6	0.198564357	-0.0365717	0.263032653	1.119890014	0.964088971	1.3008692	0.49641089
Type 2 diabetes	hypoglycemia	MR Egger	108	0.003645152	0.164232347	0.799438898	1.619043629	1.178488102	2.22429252	0.00364515
Type 2 diabetes	hypoglycemia	Weighted median	108	1.02E-26	0.369396941	0.535078703	1.571825719	1.446861808	1.70758263	5.10E-26
Type 2 diabetes	hypoglycemia	Inverse variance weighted	108	3.33E-16	0.40132539	0.655039346	1.69584708	1.493803258	1.92521827	8.33E-16
Type 2 diabetes	hypoglycemia	Simple mode	108	8.16E-07	0.289725448	0.636112165	1.588704344	1.336060619	1.88912199	1.02E-06
Type 2 diabetes	hypoglycemia	Weighted mode	108	3.89E-14	0.378068313	0.597185578	1.628447237	1.45946264	1.8169978	6.48E-14
hypoglycemia	Cushing syndrome	MR Egger	9	0.984993886	-0.50102932	0.511093908	1.005044976	0.605906666	1.66711387	0.98499389
hypoglycemia	Cushing syndrome	Weighted median	9	0.657197129	-0.23799413	0.3773129	1.072142933	0.788207322	1.45836056	0.82149641
hypoglycemia	Cushing syndrome	Inverse variance weighted	9	0.185180969	-0.07799189	0.403447403	1.176716294	0.92497193	1.49697649	0.82149641
hypoglycemia	Cushing syndrome	Simple mode	9	0.561051821	-0.31221069	0.591952256	1.150125174	0.731827323	1.8075137	0.82149641
hypoglycemia	Cushing syndrome	Weighted mode	9	0.543842191	-0.2005345	0.392251811	1.100603487	0.818293256	1.48031042	0.82149641
osteoporosis	Cushing syndrome	MR Egger	14	0.201999067	-41.3782813	224.4321357	5.62E+39	1.07E-18	2.95E+97	0.96901234

osteoporosis	Cushing syndrome	Weighted median	14	0.694589071	-26.8435109	40.29245557	832.5325469	2.20E-12	3.1535E+17	0.96901234
osteoporosis	Cushing syndrome	Inverse variance weighted	14	0.675307251	-19.888698	30.70013323	222.6759633	2.30E-09	2.1523E+13	0.96901234
osteoporosis	Cushing syndrome	Simple mode	14	0.969012337	-55.8523376	58.1558719	3.163778827	5.54E-25	1.81E+25	0.96901234
osteoporosis	Cushing syndrome	Weighted mode	14	0.876935299	-53.4212126	62.78496608	107.9725201	6.30E-24	1.85E+27	0.96901234
gout	Cushing syndrome	MR Egger	18	0.96465075	-26.0831243	27.30943958	1.846251984	4.70E-12	7.25E+11	0.96465075
gout	Cushing syndrome	Weighted median	18	0.694413491	-25.1419246	16.74574046	0.015024215	1.21E-11	18731936.5	0.96465075
gout	Cushing syndrome	Inverse variance weighted	18	0.450013773	-21.339832	9.466821105	0.002641244	5.40E-10	12923.7387	0.96465075
gout	Cushing syndrome	Simple mode	18	0.861177554	-32.1602059	38.56702449	24.61631202	1.08E-14	5.6162E+16	0.96465075
gout	Cushing syndrome	Weighted mode	18	0.78541831	-25.8418184	19.45000939	0.040929487	5.98E-12	279918596	0.96465075
hypothyroidism/myxoedema	Cushing syndrome	MR Egger	98	0.63764449	-12.2432495	7.486903652	0.092719828	4.82E-06	1784.51804	0.79705561
hypothyroidism/myxoedema	Cushing syndrome	Weighted median	98	0.527515562	-9.12644084	4.676941213	0.108094459	0.000108752	107.440931	0.79705561
hypothyroidism/myxoedema	Cushing syndrome	Inverse variance weighted	98	0.260439754	-7.24178913	1.959044409	0.071263403	0.00071603	7.09254625	0.79705561

hypothyroidism/myxoedema	Cushing syndrome	Simple mode	98	0.884445591	-14.4745396	16.79966542	3.198119267	5.17E-07	19769786.9	0.88444559
hypothyroidism/myxoedema	Cushing syndrome	Weighted mode	98	0.474843252	-11.4821774	5.328875199	0.046113428	1.03E-05	206.205903	0.79705561
hyperthyroidism/thyrotoxicosis	Cushing syndrome	MR Egger	9	0.922361904	-98.9604178	109.716534	216.6012531	1.05E-43	4.46E+47	0.92332579
hyperthyroidism/thyrotoxicosis	Cushing syndrome	Weighted median	9	0.923325787	-51.6907137	46.85181034	0.088970388	3.56E-23	2.23E+20	0.92332579
hyperthyroidism/thyrotoxicosis	Cushing syndrome	Inverse variance weighted	9	0.672759256	-44.6554039	28.82168602	0.000364546	4.04E-20	3.2893E+12	0.92332579
hyperthyroidism/thyrotoxicosis	Cushing syndrome	Simple mode	9	0.757036643	-65.1056484	90.52953617	331685.9492	5.31E-29	2.07E+39	0.92332579
hyperthyroidism/thyrotoxicosis	Cushing syndrome	Weighted mode	9	0.854710243	-67.3732623	55.51584825	0.002661921	5.50E-30	1.29E+24	0.92332579
Hypertension	Cushing syndrome	MR Egger	11	0.523845644	-356.85816	176.4229968	6.59E-40	1.04E-155	4.16E+76	0.83481834
Hypertension	Cushing syndrome	Weighted median	11	0.595344618	-73.7465957	128.5685249	8.02473E+11	9.38E-33	6.86E+55	0.83481834
Hypertension	Cushing syndrome	Inverse variance weighted	11	0.418027656	-50.6837699	122.0589142	3.15439E+15	9.73E-23	1.02E+53	0.83481834
Hypertension	Cushing syndrome	Simple mode	11	0.79119907	-175.28192	132.5689642	5.31E-10	7.52E-77	3.75E+57	0.83481834

Hypertension	Cushing syndrome	Weighted mode	11	0.83481834	-116.624393	145.2184892	1618560.886	2.24E-51	1.17E+63	0.83481834
Disorders of lipoid metabolism	Cushing syndrome	MR Egger	6	0.659022566	-1.14839456	0.699731226	0.799050071	0.317145519	2.01321154	0.73135131
Disorders of lipoid metabolism	Cushing syndrome	Weighted median	6	0.541097693	-0.46678375	0.244878158	0.894980996	0.627015669	1.27746565	0.73135131
Disorders of lipoid metabolism	Cushing syndrome	Inverse variance weighted	6	0.500647949	-0.39642262	0.193664346	0.903590382	0.672722328	1.21368883	0.73135131
Disorders of lipoid metabolism	Cushing syndrome	Simple mode	6	0.731351308	-0.54688151	0.375912865	0.918067536	0.578751831	1.45632023	0.73135131
Disorders of lipoid metabolism	Cushing syndrome	Weighted mode	6	0.578833351	-0.551548	0.295244631	0.879719929	0.576057382	1.34345497	0.73135131
Type 2 diabetes	Cushing syndrome	MR Egger	108	0.913238072	-0.60399174	0.675275293	1.036284558	0.546625288	1.96457374	0.91323807
Type 2 diabetes	Cushing syndrome	Weighted median	108	0.781638036	-0.35919944	0.477530578	1.060950887	0.698235082	1.61208856	0.91323807
Type 2 diabetes	Cushing syndrome	Inverse variance weighted	108	0.701493857	-0.2045525	0.304008542	1.05098521	0.815011951	1.35528063	0.91323807
Type 2 diabetes	Cushing syndrome	Simple mode	108	0.882912875	-0.97254726	0.836302427	0.934146113	0.378118645	2.30781785	0.91323807
Type 2 diabetes	Cushing syndrome	Weighted mode	108	0.817785918	-0.4911282	0.622335908	1.06780363	0.611935622	1.8632754	0.91323807
hypoglycemia	osteoporosis	MR Egger	7	0.258893627	-0.00104417	0.004916497	1.001938039	0.998956374	1.0049286	0.42252015

hypoglycemia	osteoporosis	Weighted median	7	0.092904664	-0.00014001	0.001821999	1.000841349	0.999860002	1.00182366	0.23226166
hypoglycemia	osteoporosis	Inverse variance weighted	7	0.338016118	-0.0007237	0.00210779	1.000692287	0.999276566	1.00211001	0.42252015
hypoglycemia	osteoporosis	Simple mode	7	0.584589951	-0.00375625	0.002046452	0.999145467	0.996250796	1.00204855	0.58458995
hypoglycemia	osteoporosis	Weighted mode	7	0.085457028	5.38E-05	0.002235634	1.001145358	1.000053772	1.00223813	0.23226166
Cushing syndrome	osteoporosis	MR Egger	3	0.613813422	-0.00832623	0.01745281	1.004573715	0.991708332	1.017606	0.61381342
Cushing syndrome	osteoporosis	Weighted median	3	0.279115175	-0.00045404	0.001573827	1.00056005	0.999546062	1.00157507	0.45896349
Cushing syndrome	osteoporosis	Inverse variance weighted	3	0.271479593	-0.00035029	0.001245735	1.000447825	0.999649776	1.00124651	0.45896349
Cushing syndrome	osteoporosis	Simple mode	3	0.351308313	-0.00048165	0.002020488	1.000769717	0.99951847	1.00202253	0.45896349
Cushing syndrome	osteoporosis	Weighted mode	3	0.367170795	-0.00052727	0.002042976	1.000758141	0.999472871	1.00204506	0.45896349
gout	osteoporosis	MR Egger	18	0.550907443	-0.07112332	0.135284274	1.032600604	0.931347035	1.14486219	0.55090744
gout	osteoporosis	Weighted median	18	0.325900361	-0.0298458	0.089831101	1.03044696	0.970595187	1.09398949	0.48659994
gout	osteoporosis	Inverse variance weighted	18	0.057970615	-0.00206041	0.123982394	1.062857453	0.997941709	1.13199594	0.28985308
gout	osteoporosis	Simple mode	18	0.389279956	-0.06733883	0.177875154	1.056823977	0.934878384	1.19467616	0.48659994
gout	osteoporosis	Weighted mode	18	0.272345704	-0.02611211	0.097878782	1.036534913	0.974225863	1.10282909	0.48659994

hypothyroidism/myxoedema	osteoporosis	MR Egger	98	0.424953669	-0.01892283	0.045093405	1.013171277	0.981255087	1.04612557	0.42495367
hypothyroidism/myxoedema	osteoporosis	Weighted median	98	0.019881547	0.004038775	0.046993297	1.025844357	1.004046942	1.04811498	0.04970387
hypothyroidism/myxoedema	osteoporosis	Inverse variance weighted	98	0.109675032	-0.0027338	0.027005758	1.01220992	0.997269936	1.02737372	0.18279172
hypothyroidism/myxoedema	osteoporosis	Simple mode	98	0.311414804	-0.02469537	0.078023783	1.027022877	0.975607066	1.08114837	0.3892685
hypothyroidism/myxoedema	osteoporosis	Weighted mode	98	0.009190404	0.010890233	0.072036473	1.042334963	1.010949747	1.07469454	0.04595202
hyperthyroidism/thyrotoxicosis	osteoporosis	MR Egger	12	0.107160276	-0.01671539	0.328067873	1.168447846	0.983423536	1.3882832	0.10716028
hyperthyroidism/thyrotoxicosis	osteoporosis	Weighted median	12	2.67E-05	0.103305573	0.284116207	1.213745327	1.108830186	1.32858731	0.00013365
hyperthyroidism/thyrotoxicosis	osteoporosis	Inverse variance weighted	12	0.000681703	0.067380968	0.251211107	1.172685054	1.069702923	1.28558145	0.00170426
hyperthyroidism/thyrotoxicosis	osteoporosis	Simple mode	12	0.023802003	0.046672266	0.323614281	1.203390842	1.04777856	1.3821141	0.0297525
hyperthyroidism/thyrotoxicosis	osteoporosis	Weighted mode	12	0.001872587	0.097507978	0.279235455	1.207282199	1.102420237	1.32211861	0.00312098

Hypertension	osteoporosis	Wald ratio	1	0.173999488	-0.23622671	1.305744544	1.707036659	0.789601646	3.69043576	0.17399949
Disorders of lipoid metabolism	osteoporosis	MR Egger	4	0.447973686	-0.00195555	0.005532472	1.001790063	0.998046364	1.0055478	0.64613166
Disorders of lipoid metabolism	osteoporosis	Weighted median	4	0.490610079	-0.00187622	0.000899864	0.999511939	0.998125534	1.00090027	0.64613166
Disorders of lipoid metabolism	osteoporosis	Inverse variance weighted	4	0.646131662	-0.00142506	0.000884117	0.999729567	0.998575959	1.00088451	0.64613166
Disorders of lipoid metabolism	osteoporosis	Simple mode	4	0.441961247	-0.00298151	0.001128535	0.99907394	0.997022927	1.00112917	0.64613166
Disorders of lipoid metabolism	osteoporosis	Weighted mode	4	0.522885688	-0.00245952	0.001136316	0.999338617	0.997543504	1.00113696	0.64613166
Type 2 diabetes	osteoporosis	MR Egger	109	0.4868219	-0.00336737	0.001599194	0.999116305	0.996638298	1.00160047	0.60852737
Type 2 diabetes	osteoporosis	Weighted median	109	0.267850557	-0.00224034	0.00062213	0.999191222	0.997762167	1.00062232	0.59962083
Type 2 diabetes	osteoporosis	Inverse variance weighted	109	0.005434024	-0.00247335	-0.000427927	0.998550414	0.997529707	0.99957216	0.02717012
Type 2 diabetes	osteoporosis	Simple mode	109	0.762429856	-0.00376138	0.00275397	0.999496423	0.996245688	1.00275777	0.76242986
Type 2 diabetes	osteoporosis	Weighted mode	109	0.359772499	-0.00243052	0.000878018	0.99922405	0.997572431	1.0008784	0.59962083
hypoglycemia	gout	MR Egger	6	0.564758012	-0.00114473	0.00222124	1.000538399	0.998855923	1.00222371	0.78429554

hypoglycemia	gout	Weighted median	6	0.627436431	-0.00068707	0.001139328	1.000226154	0.999313164	1.00113998	0.78429554
hypoglycemia	gout	Inverse variance weighted	6	0.803400994	-0.00070561	0.000910929	1.000102667	0.999294643	1.00091134	0.80340099
hypoglycemia	gout	Simple mode	6	0.558044881	-0.00219216	0.001129278	0.999468702	0.997810245	1.00112992	0.78429554
hypoglycemia	gout	Weighted mode	6	0.524712108	-0.00060535	0.001253512	1.000324135	0.999394835	1.0012543	0.78429554
Cushing syndrome	gout	MR Egger	3	0.868149139	-0.01070107	0.013271023	1.001285803	0.989355984	1.01335947	0.86814914
Cushing syndrome	gout	Weighted median	3	0.50992886	-0.0006064	0.001220642	1.00030717	0.999393789	1.00122139	0.67637098
Cushing syndrome	gout	Inverse variance weighted	3	0.495157606	-0.00048382	0.001000342	1.000258295	0.9995163	1.00100084	0.67637098
Cushing syndrome	gout	Simple mode	3	0.541096782	-0.00059276	0.001297045	1.000352203	0.999407413	1.00129789	0.67637098
Cushing syndrome	gout	Weighted mode	3	0.540160759	-0.00060788	0.00133311	1.00036268	0.999392303	1.001334	0.67637098
osteoporosis	gout	MR Egger	14	0.413155649	-0.80183277	0.317626129	0.784975069	0.448506202	1.37386252	0.66003179
osteoporosis	gout	Weighted median	14	0.550506146	-0.07101367	0.13322255	1.031593237	0.931449158	1.14250423	0.66003179
osteoporosis	gout	Inverse variance weighted	14	0.506531553	-0.0650992	0.131843486	1.033935239	0.93697451	1.14092973	0.66003179
osteoporosis	gout	Simple mode	14	0.541539316	-0.22772888	0.117346696	0.946304302	0.796340138	1.12450923	0.66003179
osteoporosis	gout	Weighted mode	14	0.660031789	-0.18462951	0.115664597	0.966105289	0.831412258	1.12261928	0.66003179

hypothyroidism/myxoedema	gout	MR Egger	98	0.325399366	-0.0155039	0.047053437	1.015899845	0.984615662	1.04817802	0.81349842
hypothyroidism/myxoedema	gout	Weighted median	98	0.813900802	-0.01789324	0.022777848	1.002445291	0.982265898	1.02303924	0.8139008
hypothyroidism/myxoedema	gout	Inverse variance weighted	98	0.160715247	-0.00413494	0.02494832	1.010461027	0.995873595	1.02526213	0.80357624
hypothyroidism/myxoedema	gout	Simple mode	98	0.574914068	-0.02837266	0.05122625	1.011492332	0.972026068	1.05256101	0.8139008
hypothyroidism/myxoedema	gout	Weighted mode	98	0.725200772	-0.01849332	0.026604948	1.004064049	0.981676631	1.02696202	0.8139008
hyperthyroidism/thyrotoxicosis	gout	MR Egger	12	0.031452011	0.032427866	0.267871962	1.162008431	1.032959379	1.30717976	0.03931501
hyperthyroidism/thyrotoxicosis	gout	Weighted median	12	0.005058685	0.038422749	0.217032998	1.136243759	1.039170448	1.2423851	0.01264671
hyperthyroidism/thyrotoxicosis	gout	Inverse variance weighted	12	3.58E-06	0.089807554	0.221451078	1.168393018	1.093963735	1.2478862	1.79E-05
hyperthyroidism/thyrotoxicosis	gout	Simple mode	12	0.087763725	-0.00673503	0.299214271	1.157473509	0.993287598	1.3487986	0.08776373
hyperthyroidism/thyrotoxicosis	gout	Weighted mode	12	0.018603934	0.040729875	0.240667801	1.151077935	1.041570713	1.27209838	0.03100656

Hypertension	gout	Wald ratio	1	0.555753411	-0.93247858	0.501450591	0.80612699	0.393576989	1.65111463	0.55575341
Disorders of lipoid metabolism	gout	MR Egger	4	0.841223775	-0.00577673	0.004575506	0.99939957	0.994239926	1.00458599	0.96032125
Disorders of lipoid metabolism	gout	Weighted median	4	0.594803137	-0.00108471	0.001892697	1.000404074	0.998915876	1.00189449	0.96032125
Disorders of lipoid metabolism	gout	Inverse variance weighted	4	0.321133503	-0.00068553	0.002090954	1.00070296	0.999314707	1.00209314	0.96032125
Disorders of lipoid metabolism	gout	Simple mode	4	0.96032125	-0.00223378	0.002113968	0.999940096	0.997768714	1.0021162	0.96032125
Disorders of lipoid metabolism	gout	Weighted mode	4	0.77791307	-0.00144059	0.001978704	1.000269094	0.998560448	1.00198066	0.96032125
Type 2 diabetes	gout	MR Egger	108	0.626673489	-0.00249154	0.004142795	1.00082597	0.997511564	1.00415139	0.77525736
Type 2 diabetes	gout	Weighted median	108	0.775257357	-0.00113649	0.001524044	1.000193793	0.998864151	1.00152521	0.77525736
Type 2 diabetes	gout	Inverse variance weighted	108	0.256795043	-0.00057348	0.002148119	1.000787629	0.999426684	1.00215043	0.77525736
Type 2 diabetes	gout	Simple mode	108	0.479144376	-0.00179877	0.003842945	1.001022608	0.998202842	1.00385034	0.77525736
Type 2 diabetes	gout	Weighted mode	108	0.362356362	-0.00083262	0.002290091	1.000729	0.999167725	1.00229272	0.77525736

hypoglycemia	hypothyroidism/myxoedema	MR Egger	8	0.700554328	-0.02553302	0.038771883	1.00664139	0.974790194	1.03953332	0.70055433
hypoglycemia	hypothyroidism/myxoedema	Weighted median	8	7.64E-07	0.00456218	0.010554598	1.007587026	1.004572603	1.01061049	3.82E-06
hypoglycemia	hypothyroidism/myxoedema	Inverse variance weighted	8	0.125455536	-0.00315473	0.025757415	1.011365441	0.996850236	1.026092	0.20909256
hypoglycemia	hypothyroidism/myxoedema	Simple mode	8	0.445308263	-0.00519699	0.002161096	0.998483202	0.994816486	1.00216343	0.55663533
hypoglycemia	hypothyroidism/myxoedema	Weighted mode	8	9.77E-05	0.007615936	0.012630528	1.010174645	1.007645011	1.01271063	0.00024423
Cushing syndrome	hypothyroidism/myxoedema	MR Egger	3	0.524610406	-0.06535218	0.023429049	0.9792566	0.936737504	1.02370566	0.52461041
Cushing syndrome	hypothyroidism/myxoedema	Weighted median	3	0.078433212	-0.00372697	0.000200533	0.998238336	0.996279968	1.00020055	0.3266512
Cushing syndrome	hypothyroidism/myxoedema	Inverse variance weighted	3	0.14219862	-0.00444796	0.000638904	0.998097286	0.995561921	1.00063911	0.3266512
Cushing syndrome	hypothyroidism/myxoedema	Simple mode	3	0.195990722	-0.00582004	7.18E-05	0.997130011	0.994196866	1.00007181	0.3266512
Cushing syndrome	hypothyroidism/myxoedema	Weighted mode	3	0.322330565	-0.00560532	0.001128098	0.997763895	0.994410364	1.00112873	0.40291321

osteoporosis	hypothyroidism/myxoedema	MR Egger	14	0.459629781	-0.89067475	2.028579165	1.766415247	0.410378757	7.60327568	0.45962978
osteoporosis	hypothyroidism/myxoedema	Weighted median	14	0.222914913	-0.07471588	0.320442874	1.130730061	0.928007112	1.37773779	0.45962978
osteoporosis	hypothyroidism/myxoedema	Inverse variance weighted	14	0.434851898	-0.15116656	0.351404009	1.105302137	0.859704494	1.42106133	0.45962978
osteoporosis	hypothyroidism/myxoedema	Simple mode	14	0.23395149	-0.11372919	0.512645659	1.220741226	0.892499625	1.66970282	0.45962978
osteoporosis	hypothyroidism/myxoedema	Weighted mode	14	0.309228723	-0.1400244	0.468646878	1.178581076	0.86933702	1.59783067	0.45962978
gout	hypothyroidism/myxoedema	MR Egger	18	0.391062781	-0.93842642	0.356152426	0.747413279	0.391243004	1.42782517	0.6517713
gout	hypothyroidism/myxoedema	Weighted median	18	0.102784231	-0.18175202	0.016623958	0.920752481	0.833808085	1.01676291	0.49549218
gout	hypothyroidism/myxoedema	Inverse variance weighted	18	0.658586567	-0.32168389	0.508941338	1.098151953	0.724927311	1.66352915	0.75479777
gout	hypothyroidism/myxoedema	Simple mode	18	0.754797774	-0.23446818	0.169110994	0.967849584	0.790991404	1.18425158	0.75479777
gout	hypothyroidism/myxoedema	Weighted mode	18	0.198196871	-0.1696905	0.031941529	0.933443831	0.843925967	1.03245713	0.49549218

hyperthyroidism/thyrotoxicosis	hypothyroidism/myxoedema	MR Egger	12	0.488885422	-1.18034348	2.546613166	1.980075243	0.307173214	12.7638016	0.48888542
hyperthyroidism/thyrotoxicosis	hypothyroidism/myxoedema	Weighted median	12	4.31E-25	0.833194557	1.222613547	2.795201095	2.300656592	3.39605189	2.16E-24
hyperthyroidism/thyrotoxicosis	hypothyroidism/myxoedema	Inverse variance weighted	12	0.000585794	0.84151481	3.073245031	7.080750613	2.319878492	21.6119204	0.00097632
hyperthyroidism/thyrotoxicosis	hypothyroidism/myxoedema	Simple mode	12	0.002188684	0.979603625	2.888199785	6.916443588	2.663400328	17.9609469	0.00273586
hyperthyroidism/thyrotoxicosis	hypothyroidism/myxoedema	Weighted mode	12	5.36E-08	0.85021633	1.153922079	2.723912333	2.340153041	3.17060392	1.34E-07
Hypertension	hypothyroidism/myxoedema	MR Egger	8	0.766721801	-1.31276642	1.806894684	1.280261214	0.269074651	6.09150199	0.9179396
Hypertension	hypothyroidism/myxoedema	Weighted median	8	0.870458434	-0.57638188	0.680998635	1.053700631	0.561927816	1.9758499	0.9179396
Hypertension	hypothyroidism/myxoedema	Inverse variance weighted	8	0.540760693	-0.36751819	0.700962012	1.181425679	0.692450731	2.01569089	0.9179396
Hypertension	hypothyroidism/myxoedema	Simple mode	8	0.901474798	-0.86102827	0.981707795	1.062197381	0.422727181	2.66901048	0.9179396
Hypertension	hypothyroidism/myxoedema	Weighted mode	8	0.917939601	-0.8967572	1.000123479	1.05304202	0.407890222	2.7186175	0.9179396

Disorders of lipoid metabolism	hypothyroidism/myxoedema	MR Egger	5	0.974041456	-0.00586232	0.005654762	0.999896227	0.994154831	1.00567078	0.97404146
Disorders of lipoid metabolism	hypothyroidism/myxoedema	Weighted median	5	0.737893627	-0.002631	0.001863604	0.999616377	0.99737246	1.00186534	0.97404146
Disorders of lipoid metabolism	hypothyroidism/myxoedema	Inverse variance weighted	5	0.968145771	-0.00178566	0.001859937	1.000037139	0.998215934	1.00186167	0.97404146
Disorders of lipoid metabolism	hypothyroidism/myxoedema	Simple mode	5	0.505581555	-0.00411876	0.001882232	0.998882364	0.995889715	1.001884	0.97404146
Disorders of lipoid metabolism	hypothyroidism/myxoedema	Weighted mode	5	0.604935557	-0.00381276	0.002116606	0.999152282	0.996194499	1.00211885	0.97404146
Type 2 diabetes	hypothyroidism/myxoedema	MR Egger	109	0.91111524	-0.00939577	0.008380904	0.999492694	0.990648228	1.00841612	0.91111524
Type 2 diabetes	hypothyroidism/myxoedema	Weighted median	109	0.302637112	-0.00126448	0.004069918	1.001403705	0.998736323	1.00407821	0.46950263
Type 2 diabetes	hypothyroidism/myxoedema	Inverse variance weighted	109	0.16742546	-0.00108381	0.006247847	1.002585357	0.998916781	1.00626741	0.41856365
Type 2 diabetes	hypothyroidism/myxoedema	Simple mode	109	0.11424643	-0.00986952	0.001021684	0.995585856	0.990179029	1.00102221	0.41856365
Type 2 diabetes	hypothyroidism/myxoedema	Weighted mode	109	0.375602106	-0.00153151	0.004077704	1.001273908	0.998469663	1.00408603	0.46950263

hypoglycemia	hyperthyroidism/thyrotoxicosis	MR Egger	6	0.066801904	0.001602229	0.013243901	1.007450684	1.001603513	1.01333199	0.08350238
hypoglycemia	hyperthyroidism/thyrotoxicosis	Weighted median	6	7.90E-32	0.00408759	0.005725878	1.004918792	1.004095955	1.0057423	3.95E-31
hypoglycemia	hyperthyroidism/thyrotoxicosis	Inverse variance weighted	6	0.010007306	0.000943882	0.006954533	1.003957016	1.000944327	1.00697877	0.01667884
hypoglycemia	hyperthyroidism/thyrotoxicosis	Simple mode	6	0.87226774	-0.00546472	0.004596175	0.99956582	0.994550181	1.00460675	0.87226774
hypoglycemia	hyperthyroidism/thyrotoxicosis	Weighted mode	6	5.73E-05	0.004116048	0.005641394	1.004890641	1.004124531	1.00565734	0.00014321
Cushing syndrome	hyperthyroidism/thyrotoxicosis	MR Egger	3	0.94879203	-0.00926196	0.008530205	0.999634187	0.990780796	1.00856669	0.94879203
Cushing syndrome	hyperthyroidism/thyrotoxicosis	Weighted median	3	0.317279384	-0.00101273	0.000328423	0.999657906	0.998987785	1.00032848	0.59409312
Cushing syndrome	hyperthyroidism/thyrotoxicosis	Inverse variance weighted	3	0.222339244	-0.00089384	0.000207886	0.999657082	0.999106559	1.00020791	0.59409312
Cushing syndrome	hyperthyroidism/thyrotoxicosis	Simple mode	3	0.475274496	-0.00110167	0.000423388	0.999660918	0.99889894	1.00042348	0.59409312
Cushing syndrome	hyperthyroidism/thyrotoxicosis	Weighted mode	3	0.458756692	-0.00106994	0.000391293	0.999660734	0.998930633	1.00039137	0.59409312

osteoporosis	hyperthyroidism/thyrotoxicosis	MR Egger	13	0.251737476	-0.14003995	0.591642796	1.253326758	0.869323504	1.80695444	0.76736589
osteoporosis	hyperthyroidism/thyrotoxicosis	Weighted median	13	0.592257971	-0.0510469	0.089432731	1.019378283	0.950234103	1.09355377	0.95068686
osteoporosis	hyperthyroidism/thyrotoxicosis	Inverse variance weighted	13	0.306946354	-0.0262609	0.083445269	1.029004864	0.974080918	1.08702572	0.76736589
osteoporosis	hyperthyroidism/thyrotoxicosis	Simple mode	13	0.933100203	-0.12506634	0.136506572	1.005736505	0.882438356	1.14626241	0.95068686
osteoporosis	hyperthyroidism/thyrotoxicosis	Weighted mode	13	0.950686861	-0.12550716	0.133863923	1.004187123	0.882049449	1.14323724	0.95068686
gout	hyperthyroidism/thyrotoxicosis	MR Egger	18	0.442177302	-0.11341751	0.048367714	0.967998348	0.892777838	1.04955652	0.55272163
gout	hyperthyroidism/thyrotoxicosis	Weighted median	18	0.272064143	-0.06036195	0.01700691	0.978555748	0.941423721	1.01715235	0.55272163
gout	hyperthyroidism/thyrotoxicosis	Inverse variance weighted	18	0.875193915	-0.0538169	0.045831569	0.996015294	0.947605596	1.04689807	0.87519391
gout	hyperthyroidism/thyrotoxicosis	Simple mode	18	0.376235781	-0.12183136	0.04465062	0.962144751	0.885297652	1.04566246	0.55272163
gout	hyperthyroidism/thyrotoxicosis	Weighted mode	18	0.282513672	-0.06207406	0.017190311	0.977808073	0.939813285	1.01733891	0.55272163

hypothyroidism/myxoedema	hyperthyroidism/thyrotoxicosis	MR Egger	97	1.73E-08	0.094755756	0.183161516	1.149076569	1.099390303	1.20100837	2.88E-08
hypothyroidism/myxoedema	hyperthyroidism/thyrotoxicosis	Weighted median	97	1.28E-34	0.099683868	0.137576589	1.125953496	1.104821593	1.14748959	6.38E-34
hypothyroidism/myxoedema	hyperthyroidism/thyrotoxicosis	Inverse variance weighted	97	3.69E-22	0.082363628	0.124188567	1.108797503	1.085850584	1.13222935	9.22E-22
hypothyroidism/myxoedema	hyperthyroidism/thyrotoxicosis	Simple mode	97	0.000194714	0.064753785	0.197283588	1.139989083	1.066896306	1.21808943	0.00019471
hypothyroidism/myxoedema	hyperthyroidism/thyrotoxicosis	Weighted mode	97	4.10E-06	0.07559927	0.176845793	1.13453461	1.078530288	1.19344704	5.12E-06
Hypertension	hyperthyroidism/thyrotoxicosis	Wald ratio	1	0.557268745	-0.37290152	0.69168032	1.172794544	0.688733056	1.99706843	0.55726875
Disorders of lipoid metabolism	hyperthyroidism/thyrotoxicosis	MR Egger	3	0.967258459	-0.00281107	0.00296271	1.000075822	0.997192874	1.0029671	0.96725846
Disorders of lipoid metabolism	hyperthyroidism/thyrotoxicosis	Weighted median	3	0.660151706	-0.00081235	0.001282243	1.000234976	0.999187984	1.00128307	0.96725846
Disorders of lipoid metabolism	hyperthyroidism/thyrotoxicosis	Inverse variance weighted	3	0.437259095	-0.00052535	0.0012152	1.000344985	0.99947479	1.00121594	0.96725846
Disorders of lipoid metabolism	hyperthyroidism/thyrotoxicosis	Simple mode	3	0.896503539	-0.00118967	0.001382812	1.000096575	0.998811037	1.00138377	0.96725846

Disorders of lipoid metabolism	hyperthyroidism/thyrotoxicosis	Weighted mode	3	0.861428644	-0.00110432	0.001352347	1.000124019	0.998896285	1.00135326	0.96725846
Type 2 diabetes	hyperthyroidism/thyrotoxicosis	MR Egger	99	0.8338768	-0.00250296	0.003104619	1.000300876	0.997500173	1.00310944	0.8338768
Type 2 diabetes	hyperthyroidism/thyrotoxicosis	Weighted median	99	0.689200143	-0.00116476	0.000769973	0.999802627	0.998835921	1.00077027	0.8338768
Type 2 diabetes	hyperthyroidism/thyrotoxicosis	Inverse variance weighted	99	0.188751422	-0.00038426	0.001948467	1.000782412	0.999615818	1.00195037	0.8338768
Type 2 diabetes	hyperthyroidism/thyrotoxicosis	Simple mode	99	0.476563812	-0.00143484	0.003081386	1.000823612	0.998566189	1.00308614	0.8338768
Type 2 diabetes	hyperthyroidism/thyrotoxicosis	Weighted mode	99	0.597501408	-0.00160477	0.000921892	0.999658622	0.998396521	1.00092232	0.8338768
hypoglycemia	Hypertension	MR Egger	9	0.199331182	-0.00023722	0.001476268	1.000619715	0.999762808	1.00147736	0.27613105
hypoglycemia	Hypertension	Weighted median	9	0.220904841	-0.00019089	0.000825973	1.000317593	0.99980913	1.00082631	0.27613105
hypoglycemia	Hypertension	Inverse variance weighted	9	0.136722173	-9.92E-05	0.000724966	1.000312924	0.99990079	1.00072523	0.27613105
hypoglycemia	Hypertension	Simple mode	9	0.097518918	-3.50E-05	0.001596455	1.000781031	0.999964998	1.00159773	0.27613105
hypoglycemia	Hypertension	Weighted mode	9	0.288302761	-0.00023765	0.000894794	1.000328626	0.999762377	1.00089519	0.28830276

Cushing syndrome	Hypertension	MR Egger	4	0.24129592	-0.00396422	0.000343896	0.998191477	0.996043631	1.00034396	0.58206563
Cushing syndrome	Hypertension	Weighted median	4	0.366458039	-0.00024427	0.000661755	1.000208762	0.999755755	1.00066197	0.58206563
Cushing syndrome	Hypertension	Inverse variance weighted	4	0.654558335	-0.00031389	0.000499593	1.000092857	0.999686162	1.00049972	0.65455833
Cushing syndrome	Hypertension	Simple mode	4	0.465652508	-0.00031571	0.000783019	1.000233684	0.999684344	1.00078333	0.58206563
Cushing syndrome	Hypertension	Weighted mode	4	0.454224223	-0.00030043	0.000767746	1.000233684	0.999699613	1.00076804	0.58206563
osteoporosis	Hypertension	MR Egger	14	0.098097657	-0.43677591	0.019364128	0.811633909	0.646116203	1.01955283	0.49048828
osteoporosis	Hypertension	Weighted median	14	0.621483082	-0.07267045	0.043424594	0.985483469	0.929907234	1.04438124	0.77685385
osteoporosis	Hypertension	Inverse variance weighted	14	0.920782534	-0.04231701	0.038230143	0.997958653	0.958565858	1.03897032	0.92078253
osteoporosis	Hypertension	Simple mode	14	0.341873434	-0.05833251	0.176565743	1.060898953	0.943336228	1.19311286	0.56978906
osteoporosis	Hypertension	Weighted mode	14	0.272852637	-0.17271082	0.045333192	0.938296941	0.841380894	1.04637645	0.56978906
gout	Hypertension	MR Egger	18	0.919881946	-0.03964796	0.044009315	1.002183058	0.961127737	1.04499209	0.93646439
gout	Hypertension	Weighted median	18	0.845261968	-0.02812982	0.034351396	1.003115632	0.97226214	1.03494822	0.93646439
gout	Hypertension	Inverse variance weighted	18	0.936464395	-0.02624689	0.024195374	0.998974767	0.974094564	1.02449046	0.93646439
gout	Hypertension	Simple mode	18	0.534919068	-0.08399039	0.042964871	0.979696194	0.919440088	1.04390122	0.93646439

gout	Hypertension	Weighted mode	18	0.892402045	-0.02591045	0.029814185	1.001953775	0.974422348	1.03026308	0.93646439
hypothyroidism/myxoedema	Hypertension	MR Egger	103	0.4974046	-0.00888133	0.018339858	1.004740466	0.991157994	1.01850907	0.4974046
hypothyroidism/myxoedema	Hypertension	Weighted median	103	0.105288867	-0.00188893	0.019872495	1.009032329	0.99811285	1.02007127	0.31264856
hypothyroidism/myxoedema	Hypertension	Inverse variance weighted	103	0.134921865	-0.00153011	0.011368093	1.004931109	0.99847106	1.01143295	0.31264856
hypothyroidism/myxoedema	Hypertension	Simple mode	103	0.187589134	-0.00835457	0.043353925	1.017653695	0.991680234	1.04430744	0.31264856
hypothyroidism/myxoedema	Hypertension	Weighted mode	103	0.293022677	-0.00742307	0.024800001	1.00872632	0.992604413	1.02511008	0.36627835
hyperthyroidism/thyrotoxicosis	Hypertension	MR Egger	10	0.83246692	-0.0646249	0.080846257	1.00814366	0.937419026	1.0842042	0.97607983
hyperthyroidism/thyrotoxicosis	Hypertension	Weighted median	10	0.911352137	-0.05005796	0.056087288	1.003019214	0.951174296	1.05769	0.97607983
hyperthyroidism/thyrotoxicosis	Hypertension	Inverse variance weighted	10	0.97173793	-0.04194348	0.043487726	1.000772419	0.958923973	1.04444718	0.97607983
hyperthyroidism/thyrotoxicosis	Hypertension	Simple mode	10	0.954151687	-0.08439192	0.089640946	1.00262796	0.919070983	1.09378149	0.97607983

hyperthyroidism/thyrotoxicosis	Hypertension	Weighted mode	10	0.976079825	-0.05261388	0.054295391	1.000841108	0.94874627	1.05579643	0.97607983
Disorders of lipoid metabolism	Hypertension	MR Egger	5	0.347257939	-0.00280204	0.000773561	0.998986276	0.997201884	1.00077386	0.9912672
Disorders of lipoid metabolism	Hypertension	Weighted median	5	0.87060173	-0.00079905	0.000676428	0.999938689	0.999201265	1.00067666	0.9912672
Disorders of lipoid metabolism	Hypertension	Inverse variance weighted	5	0.748292505	-0.00047305	0.000658271	1.000092614	0.999527061	1.00065849	0.9912672
Disorders of lipoid metabolism	Hypertension	Simple mode	5	0.959755339	-0.00107367	0.001134153	1.000030241	0.998926904	1.0011348	0.9912672
Disorders of lipoid metabolism	Hypertension	Weighted mode	5	0.991267196	-0.00099653	0.000984761	0.999994115	0.999003965	1.00098525	0.9912672
Type 2 diabetes	Hypertension	MR Egger	109	0.784319413	-0.00086049	0.001140633	1.00014008	0.999139877	1.00114128	0.78431941
Type 2 diabetes	Hypertension	Weighted median	109	0.350556236	-0.00039447	0.001111961	1.000358808	0.999605603	1.00111258	0.77351119
Type 2 diabetes	Hypertension	Inverse variance weighted	109	0.133980944	-9.74E-05	0.000730176	1.000316431	0.999902591	1.00073044	0.66990472
Type 2 diabetes	Hypertension	Simple mode	109	0.50335835	-0.00209964	0.001028119	0.999464384	0.997902563	1.00102865	0.77351119
Type 2 diabetes	Hypertension	Weighted mode	109	0.61880895	-0.00067157	0.001130282	1.000229384	0.99932866	1.00113092	0.77351119

hypoglycemia	Disorders of lipoid metabolism	MR Egger	8	0.04915491	-0.57149863	-0.064588207	0.727571199	0.564678562	0.93745342	0.24577455
hypoglycemia	Disorders of lipoid metabolism	Weighted median	8	0.582351444	-0.19517818	0.109647605	0.95813625	0.822688057	1.11588477	0.7279393
hypoglycemia	Disorders of lipoid metabolism	Inverse variance weighted	8	0.962217936	-0.16577811	0.157953954	0.996095563	0.847234203	1.17111227	0.96221794
hypoglycemia	Disorders of lipoid metabolism	Simple mode	8	0.157332146	-0.05909322	0.556109764	1.282111427	0.942618893	1.7438752	0.39333037
hypoglycemia	Disorders of lipoid metabolism	Weighted mode	8	0.521106251	-0.20050767	0.097736629	0.949912388	0.81831521	1.10267234	0.7279393
Cushing syndrome	Disorders of lipoid metabolism	MR Egger	3	0.335106825	-4.31598264	0.280276716	0.132940588	0.013353421	1.32349599	0.62577768
Cushing syndrome	Disorders of lipoid metabolism	Weighted median	3	0.344273408	-0.09934683	0.284623199	1.09706473	0.905428626	1.32926107	0.62577768
Cushing syndrome	Disorders of lipoid metabolism	Inverse variance weighted	3	0.860257095	-0.16834727	0.2015735	1.016751879	0.845060318	1.22332615	0.86025709
Cushing syndrome	Disorders of lipoid metabolism	Simple mode	3	0.500622144	-0.15205639	0.368585891	1.114342727	0.858939841	1.44568881	0.62577768
Cushing syndrome	Disorders of lipoid metabolism	Weighted mode	3	0.477975128	-0.13951698	0.360192524	1.116655182	0.869778255	1.43360539	0.62577768

osteoporosis	Disorders of lipoid metabolism	MR Egger	14	0.824547264	-66.454566	83.82939364	5927.831831	1.38E-29	2.55E+36	0.97770257
osteoporosis	Disorders of lipoid metabolism	Weighted median	14	0.792278208	-18.3526383	14.00494474	0.113739245	1.07E-08	1208565.57	0.97770257
osteoporosis	Disorders of lipoid metabolism	Inverse variance weighted	14	0.977702566	-12.1723222	11.83005166	0.842707582	5.17E-06	137317.584	0.97770257
osteoporosis	Disorders of lipoid metabolism	Simple mode	14	0.875741515	-29.3829732	24.96112565	0.109599355	1.73E-13	6.9259E+10	0.97770257
osteoporosis	Disorders of lipoid metabolism	Weighted mode	14	0.84829316	-27.053124	22.15379354	0.08632248	1.78E-12	4180904903	0.97770257
gout	Disorders of lipoid metabolism	MR Egger	18	0.388279306	-22.0818134	8.323631769	0.001029079	2.57E-10	4120.09611	0.48534913
gout	Disorders of lipoid metabolism	Weighted median	18	0.111072502	-18.0929104	1.866674283	0.000299583	1.39E-08	6.466754	0.33972509
gout	Disorders of lipoid metabolism	Inverse variance weighted	18	0.869011916	-8.64125125	10.2289862	2.211934509	0.000176666	27694.4193	0.86901192
gout	Disorders of lipoid metabolism	Simple mode	18	0.289473774	-39.167943	11.11657367	8.10E-07	9.76E-18	67276.998	0.48245629
gout	Disorders of lipoid metabolism	Weighted mode	18	0.135890037	-19.466769	2.178326737	0.000176142	3.51E-09	8.83151644	0.33972509

hypothyroidism/myxoedema	Disorders of lipoid metabolism	MR Egger	100	0.87648566	-4.33485892	5.083693512	1.454143835	0.013103723	161.368975	0.87648566
hypothyroidism/myxoedema	Disorders of lipoid metabolism	Weighted median	100	0.032521147	0.300390533	6.917749514	36.93169124	1.350386074	1010.04434	0.06704997
hypothyroidism/myxoedema	Disorders of lipoid metabolism	Inverse variance weighted	100	0.036264003	0.140623376	4.255768721	9.008747489	1.150991076	70.5109996	0.06704997
hypothyroidism/myxoedema	Disorders of lipoid metabolism	Simple mode	100	0.119612992	-1.30094782	11.77422025	188.0365228	0.272273604	129861.042	0.14951624
hypothyroidism/myxoedema	Disorders of lipoid metabolism	Weighted mode	100	0.040229982	0.226869339	7.718525692	53.12765036	1.254665921	2249.64047	0.06704997
hyperthyroidism/thyrotoxicosis	Disorders of lipoid metabolism	MR Egger	9	0.231155947	-21.2970466	107.3813509	4.93139E+18	5.63E-10	4.32E+46	0.70742558
hyperthyroidism/thyrotoxicosis	Disorders of lipoid metabolism	Weighted median	9	0.480401552	-15.7147622	33.39609604	6909.599166	1.50E-07	3.1896E+14	0.70742558
hyperthyroidism/thyrotoxicosis	Disorders of lipoid metabolism	Inverse variance weighted	9	0.489438922	-14.1213118	29.50700444	2192.606552	7.37E-07	6.5272E+12	0.70742558
hyperthyroidism/thyrotoxicosis	Disorders of lipoid metabolism	Simple mode	9	0.707425583	-53.5981562	35.84620545	0.000139705	5.28E-24	3.6967E+15	0.70742558
hyperthyroidism/thyrotoxicosis	Disorders of lipoid metabolism	Weighted mode	9	0.592683778	-22.034348	39.53531913	6313.753005	2.70E-10	1.479E+17	0.70742558

Hypertension	Disorders of lipoid metabolism	MR Egger	8	0.385529966	-76.1862794	215.3857565	1.69E+30	8.18E-34	3.47E+93	0.83146562
Hypertension	Disorders of lipoid metabolism	Weighted median	8	0.665172492	-61.2049112	39.06463317	1.56E-05	2.62E-27	9.2375E+16	0.83146562
Hypertension	Disorders of lipoid metabolism	Inverse variance weighted	8	0.860341072	-43.4691468	36.30793245	0.027858778	1.32E-19	5.8659E+15	0.86034107
Hypertension	Disorders of lipoid metabolism	Simple mode	8	0.551136328	-100.615054	51.89928667	2.64E-11	2.01E-44	3.46E+22	0.83146562
Hypertension	Disorders of lipoid metabolism	Weighted mode	8	0.417959207	-91.7891871	35.77734211	6.87E-13	1.37E-40	3.4507E+15	0.83146562
Type 2 diabetes	Disorders of lipoid metabolism	MR Egger	109	0.777970263	-0.34763372	0.259999011	0.957128757	0.706357554	1.2969288	0.77797026
Type 2 diabetes	Disorders of lipoid metabolism	Weighted median	109	0.159270279	-0.05964079	0.363534966	1.16409864	0.942102885	1.43840515	0.31415674
Type 2 diabetes	Disorders of lipoid metabolism	Inverse variance weighted	109	0.000226514	0.111973923	0.366069981	1.270006415	1.118483693	1.44205616	0.00113257
Type 2 diabetes	Disorders of lipoid metabolism	Simple mode	109	0.355529536	-0.2048242	0.573113389	1.202189642	0.814790546	1.77378093	0.44441192
Type 2 diabetes	Disorders of lipoid metabolism	Weighted mode	109	0.188494043	-0.08124901	0.419066443	1.184012054	0.921964085	1.52054138	0.31415674

	β0	β1	β2	Mediating effect	Direct effect
hypothyroidism/myxoedema as the exposure,hypoglycemia as the outcome, and Type 2 diabetes as the mediator	4.359	0.856	0.53	3.731304	0.627696

Mendelian Randomization Analysis of the interaction of 91 inflammatory protein factors with these three metabolic [illegible]

exposure	outcome	TRIT	method	nsnp	b	se	pval	lo_ci	up_ci	or	or_lci95	or_uci95	FDR
ukb-b-19732	90274765	C-C motif chemokine 19 levels	Inverse variance weighted	184	1.06381	0.352249	0.002527	0.37340302	1.7542176	2.89739	1.45267	5.778924	0.0126364
ukb-b-19732	90274773	T-cell surface glycoprotein CD5 levels	Inverse variance weighted	184	0.865211	0.363294	0.017239	0.1531546	1.5772671	2.375507	1.16551	4.841706	0.0430973
ukb-b-19732	90274774	T-cell surface glycoprotein CD6 isoform levels	Inverse variance weighted	184	0.757484	0.321971	0.01864	0.12642014	1.3885472	2.132902	1.13476	4.009022	0.0836268
ukb-b-19732	90274780	C-X-C motif chemokine 10 levels	Inverse variance weighted	184	1.045837	0.328739	0.001466	0.40150851	1.6901652	2.845779	1.49408	5.420376	0.0073293
ukb-b-19732	90274781	C-X-C motif chemokine 11 levels	Inverse variance weighted	184	0.898628	0.335038	0.007315	0.24195373	1.5553023	2.456231	1.27374	4.736518	0.0182867
ukb-b-19732	90274784	C-X-C motif chemokine 9 levels	Inverse variance weighted	184	1.283714	0.296339	1.48E-05	0.70289047	1.864538	3.610023	2.01958	6.452954	7.39E-05
ukb-b-19732	90274795	Interleukin-10 levels	Inverse variance weighted	184	0.971269	0.283676	0.000617	0.41526318	1.527274	2.641293	1.51477	4.605605	0.0029614

ukb-b-19732	90274798	Interleukin-12 subunit beta levels	Inverse variance weighted	184	1.127224	0.520112	0.030214	0.10780421	2.1466436	3.087075	1.11383	8.556093	0.050357
ukb-b-19732	90274800	Interleukin-15 receptor subunit alpha levels	Inverse variance weighted	184	0.65621	0.302686	0.030162	0.06294649	1.249474	1.927474	1.06497	3.488508	0.0754043
ukb-b-19732	90274813	Interleukin-4 levels	Inverse variance weighted	184	0.716942	0.295826	0.015371	0.1371235	1.2967596	2.048159	1.14697	3.657426	0.0768528
ukb-b-19732	90274819	Leukemia inhibitory factor levels	Inverse variance weighted	184	0.633522	0.286374	0.026952	0.07222862	1.1948146	1.884234	1.0749	3.302945	0.1347581
ukb-b-19732	90274825	Macrophage inflammatory protein 1a levels	Inverse variance weighted	184	0.793139	0.308711	0.010193	0.18806578	1.3982125	2.210324	1.20691	4.047958	0.0509672
ukb-b-19732	90274829	Neurotrophin-3 levels	Inverse variance weighted	184	-0.70606	0.267459	0.008293	-1.2302848	-0.181845	0.493583	0.29221	0.833731	0.0414655
ukb-b-19732	90274830	Osteoprotegerin levels	Inverse variance weighted	184	-0.55626	0.254623	0.028915	-1.0553221	-0.057198	0.573349	0.34808	0.944407	0.0515373

ukb-b-19732	90274841	Tumor necrosis factor receptor superfamily member 9 levels	Inverse variance weighted	184	1.119766	0.340238	0.000998	0.45290029	1.7866325	3.064138	1.57287	5.969317	0.0049893
ukb-b-19732	90274844	TNF-related activation-induced cytokine levels	Inverse variance weighted	184	0.707088	0.316026	0.025258	0.08767733	1.3264982	2.028076	1.09164	3.767826	0.1227295
ebi-a-GCST006867	90274759	Adenosine Deaminase levels	Inverse variance weighted	201	-0.03343	0.016284	0.04007	-0.0653464	-0.001515	0.967122	0.93674	0.998486	0.066784
ebi-a-GCST006867	90274763	Caspase 8 levels	Inverse variance weighted	201	-0.0321	0.015719	0.041141	-0.0629095	-0.001291	0.96841	0.93903	0.99871	0.1028521
ebi-a-GCST006867	90274778	Fractalkine levels	Inverse variance weighted	201	-0.05632	0.017284	0.001119	-0.0901989	-0.022447	0.945234	0.91375	0.977803	0.0055947
ebi-a-GCST006867	90274780	C-X-C motif chemokine 10 levels	Inverse variance weighted	201	-0.03796	0.015891	0.016912	-0.0691028	-0.006811	0.962755	0.93323	0.993212	0.0585744
ebi-a-GCST006867	90274784	C-X-C motif chemokine 9 levels	Inverse variance weighted	201	-0.03499	0.016182	0.030595	-0.0667087	-0.003274	0.965614	0.93547	0.996732	0.152975

ebi-a-GCST006867	90274786	Protein S100-A12 levels	Inverse variance weighted	201	-0.03173	0.015752	0.043971	-0.0626047	-0.000856	0.968768	0.93931	0.999144	0.0776349
ebi-a-GCST006867	90274804	interleukin-18 receptor 1 levels	Inverse variance weighted	201	0.044976	0.015599	0.003936	0.01440164	0.075551	1.046003	1.01451	1.078478	0.0196815
ebi-a-GCST006867	90274821	Monocyte chemoattractant protein-1 levels	Inverse variance weighted	201	-0.04789	0.01677	0.004297	-0.0807566	-0.015018	0.953241	0.92242	0.985094	0.0053709
ebi-a-GCST006867	90274822	Monocyte chemoattractant protein 2 levels	Inverse variance weighted	201	-0.03739	0.016878	0.026762	-0.0704671	-0.004304	0.963305	0.93196	0.995706	0.044603
ebi-a-GCST006867	90274831	Oncostatin-M levels	Inverse variance weighted	201	-0.03331	0.016526	0.043845	-0.0656996	-0.000918	0.96724	0.93641	0.999082	0.0897937
ebi-a-GCST006867	90274833	Stem cell factor levels	Inverse variance weighted	201	-0.03386	0.015577	0.029732	-0.0643898	-0.003328	0.966708	0.93764	0.996678	0.1486584
ebi-a-GCST006867	90274846	Tumor necrosis factor ligand superfamily member 12 levels	Inverse variance weighted	201	-0.06994	0.016158	1.50E-05	-0.1016107	-0.038273	0.932448	0.90338	0.96245	7.50E-05

ebi-a-GCST006867	90274847	Urokinase-type plasminogen activator levels	Inverse variance weighted	201	-0.04268	0.017124	0.012696	-0.0762394	-0.009113	0.958222	0.92659	0.990928	0.0585186
finn-b-DM_HYPOGLYC	90274765	C-C motif chemokine 19 levels	Inverse variance weighted	26	-0.10722	0.031031	0.00055	-0.168039	-0.046398	0.898329	0.84532	0.954662	0.0009164
finn-b-DM_HYPOGLYC	90274778	Fractalkine levels	Inverse variance weighted	26	-0.03983	0.017858	0.025716	-0.0748318	-0.00483	0.960952	0.9279	0.995182	0.1285787
finn-b-DM_HYPOGLYC	90274790	Fibroblast growth factor 5 levels	Inverse variance weighted	26	-0.03987	0.017523	0.022881	-0.0742153	-0.005527	0.960913	0.92847	0.994488	0.1144049
finn-b-DM_HYPOGLYC	90274798	Interleukin-12 subunit beta levels	Inverse variance weighted	26	0.045882	0.017989	0.010756	0.01062326	0.0811401	1.046951	1.01068	1.084523	0.0179259
finn-b-DM_HYPOGLYC	90274800	Interleukin-15 receptor subunit alpha levels	Inverse variance weighted	26	0.0444	0.019948	0.026033	0.00530059	0.0834986	1.0454	1.00531	1.087084	0.0325417
finn-b-DM_HYPOGLYC	90274805	Interleukin-1-alpha levels	Inverse variance weighted	26	0.066723	0.025268	0.008276	0.01719721	0.1162492	1.069	1.01735	1.123276	0.0137939

finn-b-DM_HYPOGLYC	90274827	Matrix metalloproteinase-10 levels	Inverse variance weighted	26	0.042682	0.017494	0.014692	0.00839474	0.0769693	1.043606	1.00843	1.080009	0.0367306
finn-b-DM_HYPOGLYC	90274830	Osteoprotegerin levels	Inverse variance weighted	26	-0.0369	0.016644	0.026608	-0.0695274	-0.004281	0.963768	0.93283	0.995728	0.133039
finn-b-DM_HYPOGLYC	90274846	Tumor necrosis factor ligand superfamily member 12 levels	Inverse variance weighted	26	-0.032	0.015995	0.045434	-0.0633501	-0.00065	0.968507	0.93861	0.999351	0.0943806
finn-b-DM_HYPOGLYC	90274847	Urokinase-type plasminogen activator levels	Inverse variance weighted	26	-0.05208	0.021878	0.017294	-0.0949575	-0.009197	0.949255	0.90941	0.990845	0.0864721
90274770	ukb-b-19732	C-C motif chemokine 4 levels	Inverse variance weighted	20	0.002769	0.001136	0.014784	0.00054245	0.0049946	1.002772	1.00054	1.005007	0.3363446
90274767	ukb-b-19732	C-C motif chemokine 23 levels	Inverse variance weighted	26	0.002472	0.000917	0.006998	0.0006755	0.0042686	1.002475	1.00068	1.004278	0.2558731
90274787	ukb-b-19732	Fibroblast growth factor 19 levels	Inverse variance weighted	17	-0.0059	0.002075	0.004486	-0.0099642	-0.00183	0.99412	0.99009	0.998172	0.2267801

90274764	ukb-b-19732	Eotaxin levels	Inverse variance weighted	19	-0.00612	0.002772	0.027177	-0.0115563	-0.00069	0.993896	0.98851	0.99931	0.4108289
90274830	ukb-b-19732	Osteoprotegerin levels	Inverse variance weighted	13	-0.00848	0.002795	0.002421	-0.0139565	-0.002999	0.991558	0.98614	0.997005	0.2025737
90274795	ukb-b-19732	Interleukin-10 levels	Inverse variance weighted	16	-0.00782	0.003106	0.011799	-0.0139081	-0.001733	0.99221	0.98619	0.998268	0.3149386
90274790	ukb-b-19732	Fibroblast growth factor 5 levels	Inverse variance weighted	22	-0.00273	0.000863	0.001529	-0.0044243	-0.001043	0.99727	0.99559	0.998958	0.2025737
90274827	ukb-b-19732	Matrix metalloproteinase-10 levels	Inverse variance weighted	15	0.00198	0.000899	0.027583	0.00021853	0.0037413	1.001982	1.00022	1.003748	0.4108289
90274808	ukb-b-19732	Interleukin-20 receptor subunit alpha levels	Inverse variance weighted	7	-0.00525	0.002571	0.040964	-0.0102934	-0.000216	0.994759	0.98976	0.999784	0.4659655
90274780	ukb-b-19732	C-X-C motif chemokine 10 levels	Inverse variance weighted	20	0.016049	0.008007	0.045027	0.00035554	0.0317429	1.016179	1.00036	1.032252	0.4828569

90274773	ebi-a-GCST006867	T-cell surface glycoprotein CD5 levels	Inverse variance weighted	8	0.097579	0.041637	0.019101	0.01596999	0.1791889	1.102499	1.0161	1.196247	0.6605862
90274787	ebi-a-GCST006867	Fibroblast growth factor 19 levels	Inverse variance weighted	10	0.073901	0.030386	0.015013	0.01434417	0.1334577	1.0767	1.01445	1.142773	0.6605862
90274760	ebi-a-GCST006867	Artemin levels	Inverse variance weighted	7	0.145763	0.046632	0.001773	0.05436483	0.2371605	1.156922	1.05587	1.267645	0.2452637
90274814	ebi-a-GCST006867	Interleukin-5 levels	Inverse variance weighted	5	-0.10838	0.053403	0.042412	-0.2130476	-0.003709	0.897288	0.80812	0.996298	0.835148
90274776	ebi-a-GCST006867	Macrophage colony-stimulating factor 1 levels	Inverse variance weighted	3	0.085671	0.042084	0.041779	0.00318627	0.168155	1.089447	1.00319	1.18312	0.835148
90274788	ebi-a-GCST006867	Fibroblast growth factor 21 levels	Inverse variance weighted	8	-0.23932	0.101056	0.017877	-0.4373871	-0.041247	0.787165	0.64572	0.959593	0.6605862
90274763	ebi-a-GCST006867	Caspase 8 levels	Inverse variance weighted	3	-0.16712	0.082109	0.041811	-0.3280588	-0.006191	0.846094	0.72032	0.993828	0.835148

90274791	ebi-a-GCST006867	Fms-related tyrosine kinase 3 ligand levels	Inverse variance weighted	17	0.111657	0.038711	0.003922	0.03578406	0.1875304	1.11813	1.03643	1.206267	0.3509762
90274784	ebi-a-GCST006867	C-X-C motif chemokine 9 levels	Inverse variance weighted	6	0.137707	0.040309	0.000635	0.05870178	0.2167125	1.147639	1.06046	1.241987	0.1317173
90274780	ebi-a-GCST006867	C-X-C motif chemokine 10 levels	Inverse variance weighted	11	0.07896	0.036461	0.030341	0.00749674	0.1504237	1.082161	1.00752	1.162327	0.7869698
90274807	finn-b-DM_HYPOGLYC	Interleukin-20 levels	Inverse variance weighted	11	-0.23621	0.084003	0.004925	-0.4008527	-0.071563	0.789617	0.66975	0.930938	0.5173233
90274761	finn-b-DM_HYPOGLYC	Axin-1 levels	Inverse variance weighted	8	0.236891	0.096092	0.013692	0.04855041	0.4252322	1.267303	1.04975	1.529946	0.8899708
90274764	finn-b-DM_HYPOGLYC	Eotaxin levels	Inverse variance weighted	21	0.133808	0.059659	0.024904	0.01687642	0.2507388	1.143173	1.01702	1.284974	0.9634204
90274830	finn-b-DM_HYPOGLYC	Osteoprotegerin levels	Inverse variance weighted	17	-0.20814	0.074862	0.005431	-0.3548678	-0.061408	0.812095	0.70127	0.940439	0.5173233

90274842	finn-b-DM_HYPOGLYC	Tumor necrosis factor ligand superfamily member 14 levels	Inverse variance weighted	23	-0.09538	0.042574	0.025067	-0.1788266	-0.011937	0.909026	0.83625	0.988134	0.9634204
90274790	finn-b-DM_HYPOGLYC	Fibroblast growth factor 5 levels	Inverse variance weighted	29	0.067968	0.029672	0.021984	0.00981091	0.1261244	1.070331	1.00986	1.134423	0.9634204
90274826	finn-b-DM_HYPOGLYC	Matrix metalloproteinase-1 levels	Inverse variance weighted	24	-0.08211	0.041662	0.048745	-0.1637671	-0.000451	0.921172	0.84894	0.99955	0.9634204
90274821	finn-b-DM_HYPOGLYC	Monocyte chemoattractant protein-1 levels	Inverse variance weighted	20	0.111824	0.040436	0.005685	0.03256848	0.1910792	1.118316	1.0331	1.210555	0.5173233
90274780	finn-b-DM_HYPOGLYC	C-X-C motif chemokine 10 levels	Inverse variance weighted	21	0.306963	0.140417	0.02881	0.03174546	0.5821808	1.359291	1.03225	1.789938	0.9634204
